# Cognitive decline over time in myotonic dystrophy type 1: a systematic review of longitudinal studies

**DOI:** 10.3389/fneur.2026.1901436

**Published:** 2026-09-10

**Authors:** Ainara Clemente-Fernández, Irati Larranaga, Joana Garmendia, Andone Sistiaga, Garazi Labayru

**Affiliations:** 1 School of Postgraduates, Faculty of Psychology, Universitat Autònoma de Barcelona (UAB), Barcelona, Spain; 2 Department of Clinical and Health Psychology and Research Methodology, Faculty of Psychology, University of the Basque Country (EHU), San Sebastian, Spain; 3 Centro de Investigación Biomédica en Red sobre Enfermedades Neurodegenerativas (CIBERNED), Carlos III Health Institute, Madrid, Spain; 4 Neuroscience Area, Biogipuzkoa Health Research Institue, San Sebastián, Spain

**Keywords:** central nervous system, cognitive decline, longitudinal studies, myotonic dystrophy type 1, Steinert’s disease, systematic review

## Abstract

**Background:**

Myotonic Dystrophy Type 1 (DM1) is a multisystem genetic disorder and the most common muscular dystrophy in adults. Central nervous system involvement is well-documented in DM1, including cognitive impairment. Cross-sectional studies have described a heterogeneous neuropsychological profile; however, the trajectory of cognitive functioning over time remains unclear, highlighting the need to focus on longitudinal studies. This systematic review aimed to examine longitudinal evidence on cognitive progression in DM1 and to analyze patterns of decline across clinical phenotypes.

**Methods:**

Following PRISMA 2020 guidelines, a systematic search was conducted in three databases: PubMed, Web of Science, and Scopus. Eligible studies were original longitudinal investigations assessing cognitive performance in DM1 using standardized measures. Risk of bias was assessed using an adapted Newcastle-Ottawa Scale.

**Results:**

Sixteen studies met the inclusion criteria, with follow-up periods ranging from 1 to 20 years. In pediatric phenotypes, intellectual disability and global cognitive impairment were evident at baseline, with largely stable trajectories over time. In contrast, adult- and late-onset phenotypes reported a pattern of slow, progressive, domain-specific cognitive decline, particularly in visuoconstruction and processing speed. Age and disease duration emerged as the most consistent predictors of decline over time.

**Conclusion:**

The available evidence supports a dual-process model of cognitive dysfunction in DM1, combining early neurodevelopmental impairment with later domain-specific neurodegenerative decline. These findings highlight the importance of longitudinal cognitive monitoring and suggest that specific cognitive domains may serve as sensitive markers for disease progression and potential endpoints in clinical trials.

**Systematic review registration:**

https://www.crd.york.ac.uk/PROSPERO/view/CRD420251148417, identifier (CRD420251148417).

## 1 Introduction

Myotonic Dystrophy Type 1 (DM1), also known as Steinert’s disease, is the most common muscular dystrophy in adults (1), with an estimated global prevalence of 9.97 cases per 100,000 individuals (2). DM1 is a progressive, multisystem, autosomal-dominant disorder (3), caused by an expanded CTG trinucleotide repeat (CTG repeats) in the DMPK gene, with pathogenicity typically defined by more than 50 repeats (4, 5). Given its progressive multisystem involvement, DM1 has recently been proposed as a model of accelerated or premature biological aging (6). Although the clinical course of DM1 is variable and progressive, the most widely used classification is based on age at symptom onset, distinguishing five phenotypes: congenital (birth), childhood-onset (1–10 years), juvenile-onset (10–20 years), adult-onset (20–40 years), and late-onset DM1 (> 40 years) (7).

Both the genotype, reflected in CTG repeat length, and the clinical phenotype of DM1 vary substantially across patients (8), underscoring the complex multisystem nature of the disease. DM1 affects not only the musculoskeletal system but also the ophthalmological, cardiac, and central nervous systems, among others (1, 7, 9). Central nervous system (CNS) involvement is increasingly recognized as a core feature of the disease and includes cognitive or neuropsychological impairments as well as other CNS-related symptoms, such as apathy, fatigue, and hypersomnolence (10, 11). Cognitive deficits in DM1 predominantly affect visuospatial, visuoconstructive, and executive function domains (12–16). Nonetheless, meta-analytic evidence suggests that impairments may extend across all cognitive domains (14).

The neuropathological mechanisms underlying these symptoms remain incompletely understood (17). However, neuroimaging and neuropathology studies have identified both structural (18, 19) and functional brain abnormalities (20–22), including white and grey matter pathology, cellular alterations, and nucleotide deposits (19, 23, 24). Moreover, longitudinal neuroimaging studies suggest that brain abnormalities may worsen over time (25–27).

Two main hypotheses have been proposed to explain the etiopathogenesis underlying CNS involvement: a neurodegenerative process and a neurodevelopmental hypothesis. Evidence of progressive brain changes (25–27) and cognitive decline supports the neurodegenerative hypothesis (12–16). Conversely, studies of congenital, childhood-onset, and juvenile-onset phenotypes have described specific cognitive deficits, global intellectual impairment, and an increased prevalence of neurodevelopmental disorders, supporting a neurodevelopmental explanation (28–32).

Despite the growing body of literature, the trajectory of cognitive functioning over time in DM1 remains unclear, and, to date, no systematic review has specifically examined longitudinal cognitive studies in this population. Understanding cognitive progression is essential for clarifying the etiopathogenic mechanisms of DM1 and for better characterizing the aging process in this population. Moreover, synthesizing evidence on cognitive function in DM1 is crucial for addressing one of the most frequent and impactful concerns reported by patients, given the relevance of cognitive impairment to daily functioning and quality of life (33, 34).

Therefore, this systematic review aimed to synthesize the available longitudinal evidence on cognitive progression in DM1, assess whether sufficient evidence of cognitive decline exists, identify the cognitive domains that show progressive decline, and determine whether a consistent pattern of cognitive deterioration emerges across studies.

## 2 Method

The protocol for this systematic review was registered in the International Prospective Register of Systematic Reviews (PROSPERO; ID: CRD420251148417) and is freely available at: https://www.crd.york.ac.uk/PROSPERO/view/CRD420251148417. No amendments or deviations from the information provided at registration were made during the course of the study.

### 2.1 Search strategy

This systematic review was conducted in accordance with the Preferred Reporting Items for Systematic Reviews and Meta-analyses 2020 statement (PRISMA 2020) (35). The completed PRISMA checklist is included in Table S1 of the Supplementary material. A systematic search was conducted on September 8, 2025, in three databases: Web of Science (WoS; RRID: SCR_022706), Scopus (RRID: SCR_022559), and PubMed (RRID: SCR_004846). The following keywords and their combinations were used: *myotonic dystrophy*, *Steinert*, *DM1*, *DM-1*, *cognitive*, *cognition*, *neurodegeneration*, *neuropsychology*, *neuropsychological*, *longitudinal*, and *follow-up*. The PICO question can be consulted in Table S2 of the Supplementary material. In total, 601 records were retrieved across the three databases. The complete search strategies for each database are provided in Table 1.

**Table 1:** Search strategy for each database.

Database	Advanced search	Records retrieved (*n*)
PubMed^1^	(((myotonic dystrophy) OR (Steinert*) OR (DM1) OR (DM-1)) AND ((cognitive) OR (cognition) OR (neurodegeneration) OR (neuropsychology) OR (neuropsychological))) AND ((longitudinal) OR (follow-up))	86
WoS	((TS = ((myotonic dystrophy) OR (Steinert*) OR (DM1) OR (DM-1))) AND TS = ((cognitive) OR (cognition) OR (neurodegeneration) OR (neuropsychology) OR (neuropsychological))) AND TS = ((longitudinal) OR (follow-up))	119
Scopus	(TITLE ((myotonic AND dystrophy) OR (steinert*) OR (dm1) OR (dm-1))) AND (ALL ((cognitive) OR (cognition) OR (neurodegeneration) OR (neuropsychology) OR (neuropsychological)) AND ((longitudinal) OR (follow-up)))	396

1 For the PubMed search, the keywords and Boolean operators presented above were searched under the term “All fields”.

### 2.2 Eligibility criteria

Records were considered eligible if they met the following criteria: (a) the study was an original article; (b) cognitive performance or impairment was assessed using standardized tests; (c) the study included patients with DM1, (d) the study had a longitudinal design, with cognitive measures collected at baseline and follow-up; and (e) the article was published in English or Spanish.

### 2.3 Screening and data extraction

All 601 retrieved records were independently screened using *Rayyan* (RRID: SCR_017584), a digital tool for systematic review screening (36), by two members of the research team. One reviewer (AC) screened all retrieved records, while a second reviewer (GL) screened 72.7% of the sample. Inter-rater agreement was calculated using Cohen’s Kappa (37) in Microsoft Excel (RRID: SCR_016137), yielding a high level of agreement (*κ* = 0.939), as detailed in the Supplementary material (Table S3). Any disagreements or uncertainties regarding study inclusion were discussed and resolved by consensus with a third reviewer (JG) to reach a decision. Full texts were obtained for all studies that met the inclusion criteria during the initial screening.

For the included studies, data were extracted across 10 categories: (1) author and year of publication; (2) sample size; (3) sex/gender distribution; (4) phenotype; (5) inheritance pattern; (6) time between baseline and follow-up assessments; (7) age of participants at baseline and follow-up; (8) cognitive domains assessed; (9) tests administered; and (10) outcomes regarding longitudinal changes in cognition. Data were extracted by one reviewer (AC) and supervised by three other team members (GL, JG, and IL) to minimize errors. Regarding cognitive outcomes, the primary effect parameters sought for extraction and synthesis were statistical significance values (*p-values*) demonstrating longitudinal change, alongside reported mean scores and standardized test performance over time.

### 2.4 Risk of bias

Risk of bias in the included studies was independently assessed by two authors (AC and IL) using an adapted version of the Newcastle-Ottawa Scale (NOS). A newly modified version of the original NOS was developed to better capture DM1-specific confounders, as well as methodological characteristics of longitudinal studies including clinical samples. The adapted NOS and detailed scoring criteria are provided in the Supplementary material (Data S1). Assessment was fully performed by one reviewer (IL) and partially by a second one (AC), discussing any disagreements with a third member (JG). Strictly following PRISMA guidelines (35) and considering the specific characteristics of the risk of bias of this systematic review, no inter-rater agreement analysis was conducted in this case. The results of the risk-of-bias assessment for each included study are also provided in the Supplementary material (Table S4).

### 2.5 Data synthesis

**Figure 1:**
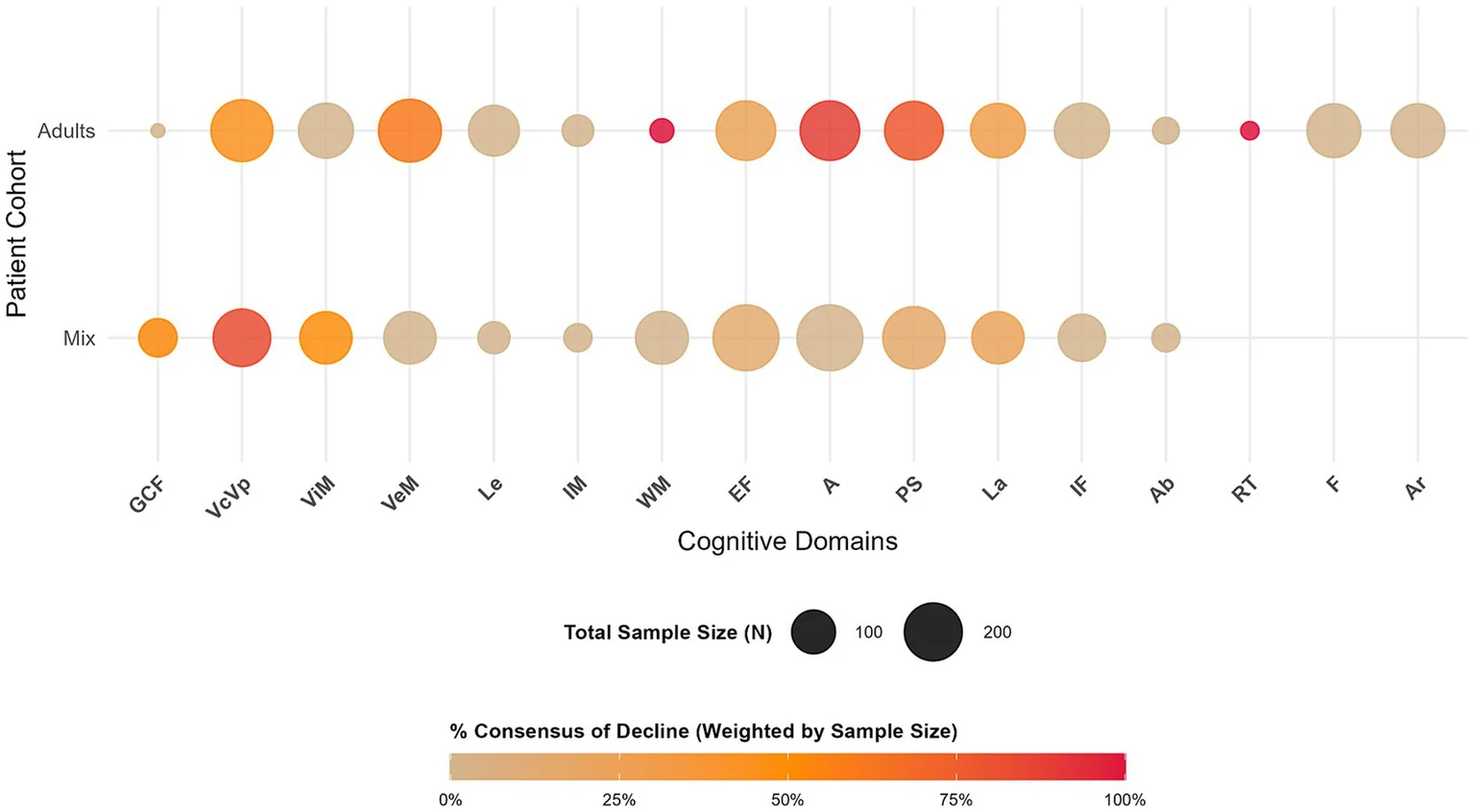
Weighted consensus synthesis of longitudinal cognitive decline. This consensus synthesis explicitly weighs the visual evidence based on the cumulative sample size (total cumulative *N* = 518 unique patients with DM1, excluding overlapping cohorts; *n* = 252 adult- and late-onset and *n* = 266 mixed-group cohorts), prioritizing data from large longitudinal cohorts and minimizing bias from underpowered studies. Bubble size: The diameter of each bubble is directly proportional to the total cumulative patients evaluated for that specific domain-cohort intersection. Larger bubbles indicate more robust evidence. Color intensity: Represents the percentage of this sample size weight derived from studies reporting statistically significant cognitive decline. Deep carmine indicates high consensus across studies; tan indicates absence of significant change across samples. GCF, general cognitive functioning; Vc/Vp, visuoconstruction/perception; ViM, visual memory; VeM, verbal memory; Le, learning; IM, immediate memory; WM, working memory; EF, executive functioning; A, attention; PS, processing speed; La, language; IF, intellectual functioning; Ab, abstract reasoning.

A qualitative synthesis was performed. Included studies were grouped for synthesis according to their phenotypic characteristics and the clinical categories of the participants. This grouping was chosen because of DM1 heterogeneity, where cognitive trajectories differ significantly between early-onset and late-onset forms. A meta-analysis was not conducted due to the high clinical and methodological heterogeneity across the included studies. Consequently, to ensure methodological rigor and transparency, the findings were structured and reported in accordance with the Synthesis Without Meta-analysis (SWiM) reporting guidelines (38) (Detailed in Supplementary Material, Table S5). The metrics used for the synthesis were the statistical significance (*p*-values) and the direction of longitudinal change. These metrics were chosen because of the lack of consistent reporting of effect sizes in primary studies, with only 3 out of 16 studies evaluating the effect size in longitudinal changes (39–41). To integrate these findings qualitatively, a weighted consensus approach was developed (presented in Figure 1). This method synthesizes the evidence by weighting the reported results against the cumulative sample size (N) of the studies contributing to each cognitive domain, prioritizing findings from larger longitudinal cohorts. No data conversions or transformations were conducted; all data was extracted and synthesized strictly as originally reported in the primary studies.

## 3 Results

### 3.1 Literature search and selection

**Figure 2:**
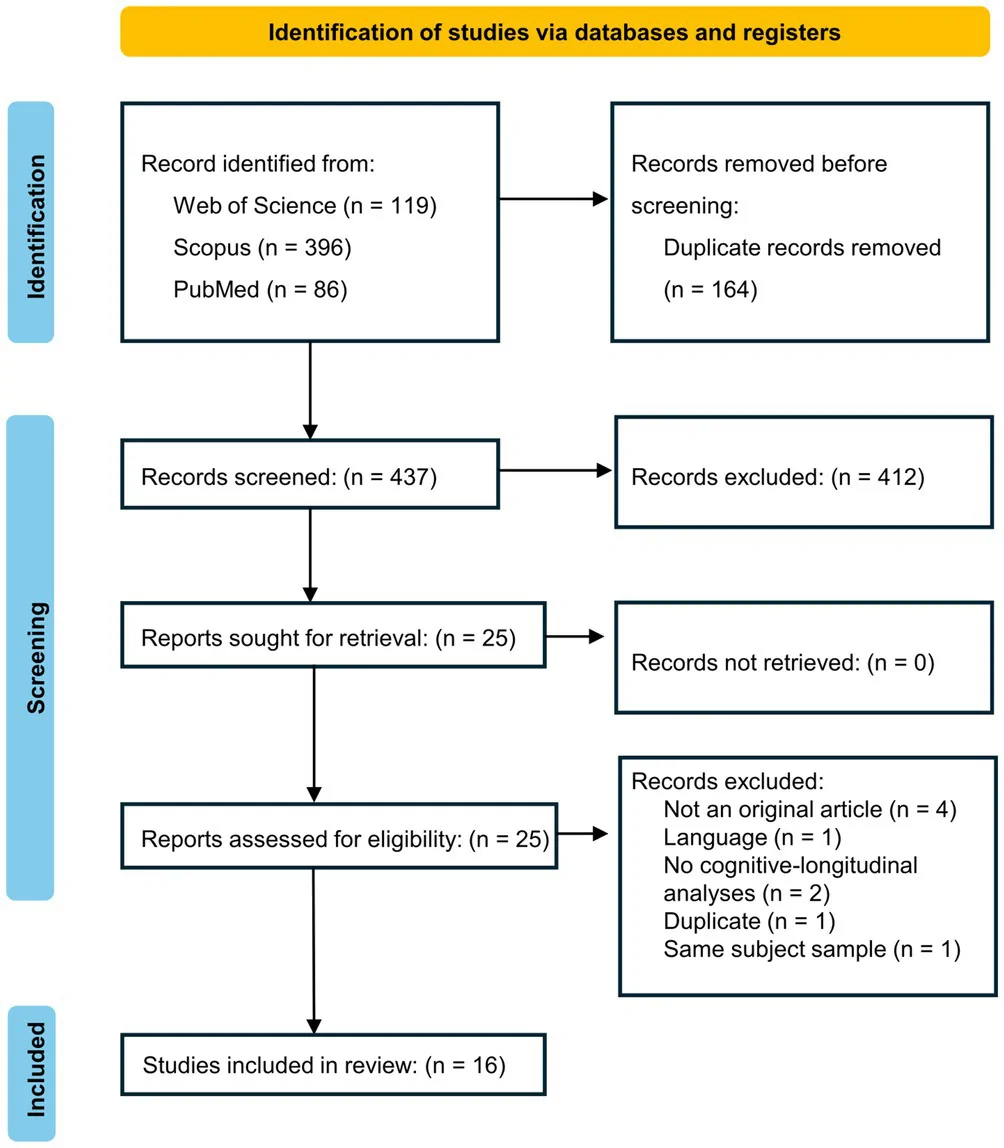
PRISMA flow diagram of the study selection process.

The literature search yielded 601 records. After duplicates were removed, 437 records were screened. Of these, 412 were excluded, leaving 25 potentially eligible studies. A list of excluded studies, including reasons for exclusion, is provided in the Supplementary material (Table S6). A further eight studies were excluded after full-text review: four were not original research articles, two did not include longitudinal cognitive assessments, one was written in a language other than English or Spanish, and one was a duplicate. This resulted in a total of 17 articles. During the initial stages of data extraction, one further study was excluded because two publications reported data from the same sample and included the same neuropsychological analyses; therefore, retaining both would have duplicated the results. As the NOS scores differed by only one point, we selected the study that focused more specifically on neuropsychological outcomes, in line with the objective of this review. Ultimately, 16 articles were included in the systematic review. The complete screening process is illustrated in Figure 2.

### 3.2 Characteristics of included studies

Across the 16 included publications, studies were grouped into three categories based on the participants’ phenotype. The first five of the included articles assessed patients with pediatric phenotype, specifically congenital and/or childhood-onset DM1 (42–46); seven studies evaluated adult or late-onset populations (27, 41, 46–50); and five studies included mixed phenotypes but reported results for DM1 as a single group (39, 40, 51, 52). Note that the total count across categories (17) exceeds the number of unique publications (16) because Tuikka et al. (46) reported results for both congenital and adult groups separately. Several studies originated from the same longitudinal DM1 cohort (27, 40, 51). Although some participant overlap may have occurred across the publications, each study addressed distinct research questions and reported different analyses and outcomes. Potential cohort overlap was considered during the qualitative synthesis and was avoided in any aggregated visual synthesis by retaining only the study with the largest sample size when overlapping cohorts contributed to the same outcome. Sample sizes ranged from 5 to 119 participants, and the interval between baseline and follow-up assessments ranged from 1 to 20 years. The mean or median age at baseline ranged from 37 to 52 years in adult samples and from 9 to 13 years in pediatric samples. The descriptive data are summarized in the Supplementary material (Table S7).

### 3.3 Methodological quality and risk of bias

The methodological quality of the 16 included studies was evaluated using the NOS, with total scores ranging from 7 to 18 points out of a maximum of 19 (see Table S4). In the Selection domain (max 6 points), scores were moderate overall, with most investigations scoring between 1 and 2 points, whereas Labayru et al. (27, 51) achieved the highest rigor, scoring 6 and 5 points, respectively.

The Comparability domain (max 3 points) represented the main methodological constraint across the evidence base, as 9 out of 16 reviewed studies scored 0 points due to a lack of matching or statistical adjustment for confounding variables. This deficit was equally observed in case–control designs and in intra-disease characterization, contrasting different DM1 phenotypes. Conversely, Garmendia et al. (40) and Labayru et al. (27, 51) achieved the maximum 3 points.

Finally, the Outcome domain (max 10 points) yielded consistently high and homogeneous scores across all investigations, reflecting a widespread utilization of standardized and validated neuropsychological tools. Overall, the cumulative evidence base can be characterized as transitioning from fair to good quality.

### 3.4 Synthesis of results

After analysis of the 16 included publications, the results of this systematic review are presented in five categories to enhance clarity: Pediatric DM1 phenotypes, comprising congenital, childhood-, and juvenile-onset DM1; adult- and late-onset DM1; DM1 analyzed as a single group; longitudinal comparisons between DM1 phenotypes; and correlates of cognitive decline over time. The synthesis focuses specifically on longitudinal findings related to cognitive impairment. Within each category, findings are structured by first outlining baseline cognitive status and then examining longitudinal outcomes. A summary of the most relevant findings is provided in Table 2.

**Table 2:** Summary of longitudinal outcomes of the included articles.

References	Sample	Phenotypes	Measured cognitive variables	Outcomes
Deutsch et al. (53)	91 DM1	Juvenile-onset onwards	Simple reaction time and psychomotor function, choice reaction time and attention, working memory, and executive function	Significant improvement in executive functioning on the GMLT at the 3-month follow-up, which remained stable between the 3-month and 1-year follow-up assessments.
Díaz-Leiva et al. (47)	24 DM1	Adult-onset	Working memory, visuospatial and visuoconstructive skills, sustained and alternating attention, verbal memory, verbal IQ, and dysexecutive symptoms	Significant worsening in working memory and visuoconstructive skills. The number of dementia cases increased, as did the number of single-domain mild cognitive impairment cases, while the number of patients with no cognitive deficits decreased.
Echenne et al. (42)	8 DM1	Congenital and post-neonatal onset	Speech, language, and IQ	Cognitive impairment remained stable in congenital forms and remained stable or worsened in post-natal forms. Language and speech delays worsened over time.
Fujino et al. (39)	66 DM1	Pediatric onset, adult onset, and late-onset	General cognitive functioning, visuoconstructive skills, psychomotor speed, attention, and executive functioning	Significant decline in general cognitive functioning, psychomotor speed, and visuoconstructive skills. Older age at assessment was associated with higher delta scores in those domains. Significant longitudinal differences in executive functioning were found between pediatric and adult-onset groups.
Gallais et al. (48)	115 DM1	Adult- and late-onset	Learning ability, verbal short- and long-term memory, visuo-spatial constructional ability, visual memory, verbal fluencies (phonemic and semantic fluencies), visual naming ability, executive cognitive control ability, sustained and selective attention, and intellectual functioning	Significant worsening in mean scores and a significant increase in the percentage of impaired scores^1^ in verbal memory, visual attention, and processing speed. Significant improvement was observed in verbal fluency and global intelligence. The rate of decline over time was significantly higher in the late-onset form than in the adult-onset form.
Garmendia et al. (40)	21 premanifest DM1 25 manifest DM1 72 HC	Premanifest, juvenile-onset, and adult-onset	Visuoconstruction, verbal memory, attention, processing speed, executive functions, language, and intellectual functioning	Premanifest individuals showed significantly poorer scores in attention/processing speed and better scores in visuoconstruction. Manifest patients showed significantly poorer scores in visuoconstruction. Healthy controls showed significantly poorer scores in executive functioning but better scores in attention/processing speed, visual memory, visuoconstruction, and IQ. No significant differences in cognitive trajectories were found between groups over time.
Gliem et al. (49)	16 DM1 16 DM2 17 HC	Adult-onset	Verbal and figural memory, semantic memory, reaction and processing speed, executive functioning, visuoconstructional skills, and phonemic verbal word fluency	Greater susceptibility to interference was observed over time. Significant between-group differences in longitudinal changes were found for reaction time, selective attention, and visuospatial and visuoconstructive skills between healthy controls and patients with DM1.
Labayru et al. (51)	75 DM1 32 HC 12 LGMD2	Childhood-, juvenile-, adult-, and late-onset	IQ, visuoconstructional and visuospatial skills, executive functioning, verbal memory, visual memory, language, attention, and processing speed	Patients with DM1 performed worse than HC in visuospatial and visuoconstructional skills and visual memory. Age and MIRS were significant predictors of progressive decline in DM1. A cut-off point of 31 years was identified for greater decline in DM1.
Labayru et al. (27)	8 DM1 10 HC	Adult- and late-onset	Attention and processing speed, verbal memory, visual memory, visuoconstruction, executive functioning, language, and intellectual functioning	Attention/processing speed showed significant decline over time.
Lindeblad et al. (43)	16 severe congenit DM1 17 mild congenit DM1 18 childhood-onset DM1	Congenital and childhood-onset	Global cognitive functioning and adaptative functioning	Intellectual disability level worsened over time in congenital groups. Deterioration on adaptative behavior was significant for both congenital forms. All groups showed poorer standard scores in the communication domain. Severe congenital groups showed significantly poorer scores in the socialization and daily living domains. Age and intellectual disability had a significant impact on changes in adaptative behavior.
Martinello et al. (44)	3 DM1	Congenital	IQ	At follow-up, the three assessed patients showed no deterioration in intellectual functioning.
Modoni et al. (52)	34 DM1	Congenital, juvenile-, and adult-onset	Visual and verbal memory, executive functioning, working memory, visual attention, and abstract reasoning	Overall change over time indicated poorer performance on verbal attainment tasks, but better performance in memory, visual reasoning, and MMSE. Decline in executive and linguistic tasks was significant, while improvement in visual and verbal memory was reported. Distinct cognitive trajectories over time were observed between groups.
Ricci et al. (45)	3 congenital DM1 3 childhood-onset DM1	Congenital and childhood-onset	IQ, visuospatial functions, attentional functions, and executive functions	Comparisons between baseline and follow-up showed a significant decrease in cognitive level in all patients.
Sansone et al. (50)	14 DM1 11 DM2	Congenital and childhood-onset excluded	Dementia screening, nonverbal reasoning, auditory language comprehension, verbal fluency, verbal and spatial short-term memory, visuoconstructional skills, attention, and executive functions	Significant worsening over time was observed in attention, executive functioning, and language, while improvement in memory was reported. Results for language and memory were significant but remained within the normal range.
Tuikka et al. (46)	5 congenital DM1 30 adult-onset DM1	Congenital and adult-onset	Verbal IQ, performance IQ, full-scale IQ, visuoconstructive IQ, memory, learning, immediate visual memory, organization of perceptual abilities, visuospatial and constructional abilities, and motor speed	Overall cognitive patterns did not change in adult-onset patients, and no selective deterioration was detected during the follow-up period.
Winblad et al. (41)	37 DM1	Adult-onset	Verbal ability, verbal fluency, arithmetical ability, picture completion, visual memory, verbal memory, speed, attention, and executive functions	After controlling for normal cognitive aging, significant worsening was found on tests of verbal memory, verbal ability, speed, attention, and executive functioning. The proportion of participants with impaired scores^1^ increased significantly over time. Cognitive decline was found in 65% of patients, whereas 22% improved.

Sample size reflects participants who completed the neuropsychological assessment at the last time point. DM1: myotonic dystrophy type 1; DM2: myotonic dystrophy type 2; HC: healthy control; LGMD2: limb-girdle muscular dystrophy type 2.

#### 3.4.1 Pediatric DM1 phenotypes (congenital and childhood-onset)

Five articles addressing congenital and/or childhood-onset DM1 were included in this category. It should be noted that while juvenile-onset cases were presented in the literature, they were exclusively reported within mixed-phenotype studies and thus analyzed as a single group. In pediatric DM1 phenotypes, CNS involvement is already evident at baseline and is characterized by a neurodevelopmental profile involving mild to moderate intellectual disability (42–46). Patients with congenital DM1 consistently showed the lowest IQ scores, with reported values ranging from 39 to 79 (42–46). Three of the five studies also examined the childhood-onset phenotype and showed that, although IQ scores tended to be slightly higher than in congenital DM1, most patients still scored below 80 (42, 43, 45).

The baseline neuropsychological profile of pediatric patients was characterized by broadly homogeneous impairment across cognitive domains, with severe delays in language acquisition and persistent difficulties in phonological and syntactic processing (42). Specific deficits were also identified in visuospatial abilities (45) and attentional functions (42, 45), whereas executive function appeared to fall within normal ranges in one study (45).

The longitudinal evolution was assessed over intervals ranging from 2 years (45) up to a maximum of 20 years in congenital cases (42). Some authors reported that intellectual functioning and other cognitive domains remained stable over time (42, 44). However, other studies indicated an increase in the proportion of patients with impaired IQ at follow-up (43, 45). Although functions such as attention and visuospatial abilities tended to remain stable, emotional and behavioral profiles appeared to deteriorate over time in some studies (42, 45).

#### 3.4.2 Adult- and late-onset

The seven longitudinal studies examining adult- and late-onset DM1 phenotypes described a baseline profile characterized by overall intellectual functioning largely within the normal range, with estimated mean IQ scores between 94 and 99 (46, 47), but with prominent deficits in specific cognitive domains. The most affected domains at baseline included visuospatial/visuoconstructive abilities, working memory, and alternating attention (46, 47, 50).

**Figure 3:**
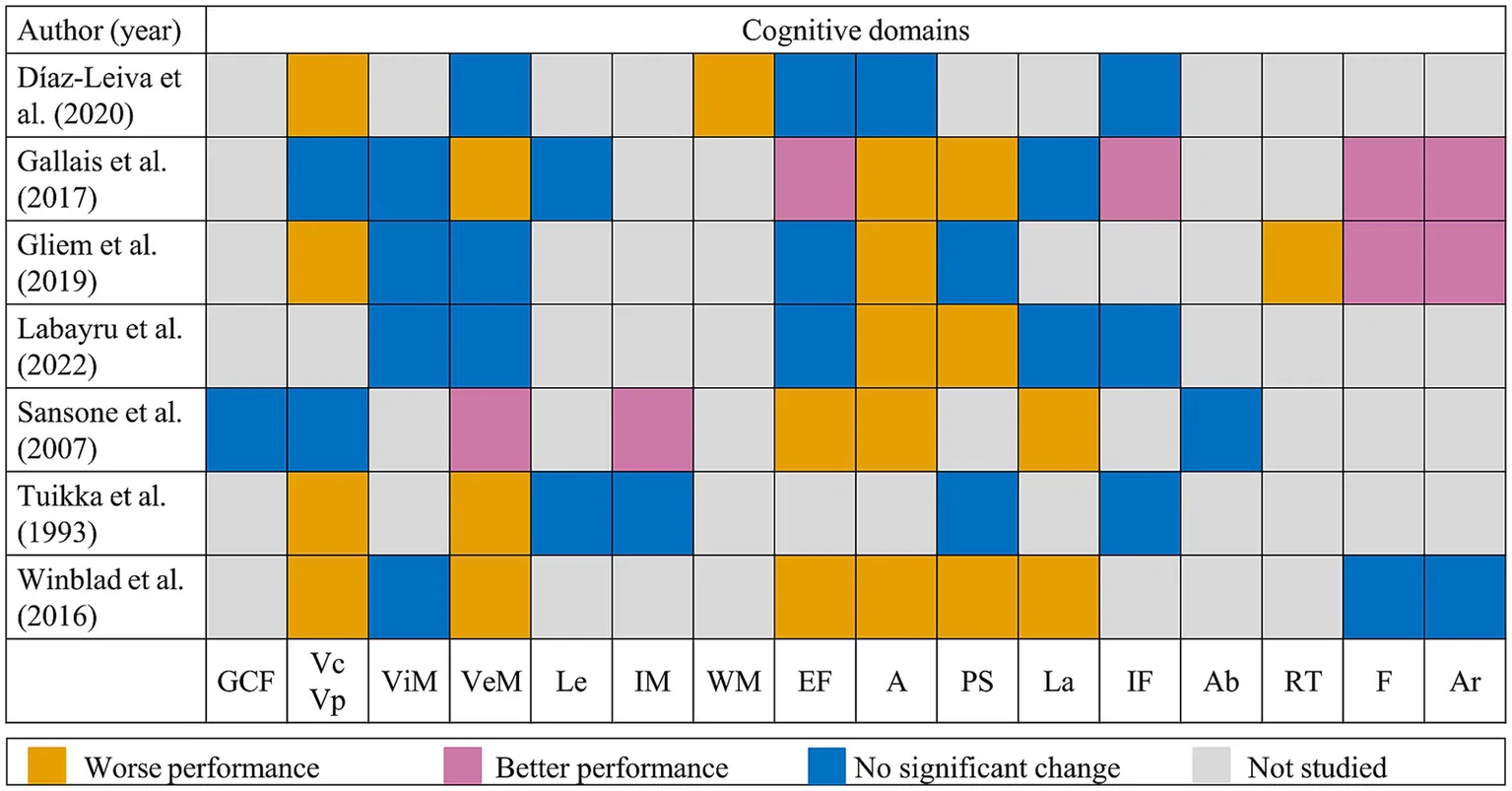
Results of studies reporting cognitive performance over time in adult- and late-onset DM1. “No significant change” indicates that numerical variation did not reach the statistical threshold and should not be interpreted as evidence of clinical or phenotypic stability over time. Furthermore, instances of “better performance” should be interpreted with caution, as longitudinal improvements may reflect retest, familiarity, or practice effects rather than true cognitive recovery. GCF, general cognitive functioning; Vc/Vp, visuoconstruction/perception; ViM, visual memory; VeM, verbal memory; Le, learning; IM, immediate memory; WM, working memory; EF, executive functioning; A, attention; PS, processing speed; La, language; IF, intellectual functioning; Ab, abstract reasoning; RT, reaction time; F, fluency; Ar, arithmetic.

Regarding longitudinal evolution, across follow-up periods ranging from 4 (47) to 12 years (46), most studies converged on a pattern of slow but progressive cognitive decline. Notably, up to 65% of patients showed measurable deterioration over time within some specific samples (41, 48). This progression was reflected in declining performance in attention (27, 41, 46–50), visuospatial/constructive abilities (41, 47, 49), processing speed (27, 41, 48), and verbal memory (41, 46, 48). A particularly relevant finding was the significant increase in the proportion of patients with cognitive impairment over time. At the 9-year follow-up, the percentages of participants performing 1 or 2 standard deviations below normative expectations ranged from 22.6 to 82.6% and from 6.1 to 59.1%, respectively, depending on the subtest (48). By contrast, functions such as global intelligence, visual memory, or language remained stable or even showed slight improvements in some studies (46, 50). The results for each study are presented in Figure 3 and the weighted consensus for cognitive decline is summarized in Figure 1.

#### 3.4.3 DM1 analyzed as a single group

This category included five articles that examined samples comprising different phenotypes but reported results from DM1 as a single group. At baseline, neuropsychological assessment consistently showed lower cognitive performance in patients with DM1 than in healthy controls, although most scores typically fell within normal limits. The most prominent impairments involved visuoconstructive abilities, processing speed, and executive functions (39, 40, 51, 53). According to Fujino et al. (39), a substantial proportion of patients (39–64%) scored two standard deviations below the mean in visuoconstruction and processing speed.

**Figure 4:**
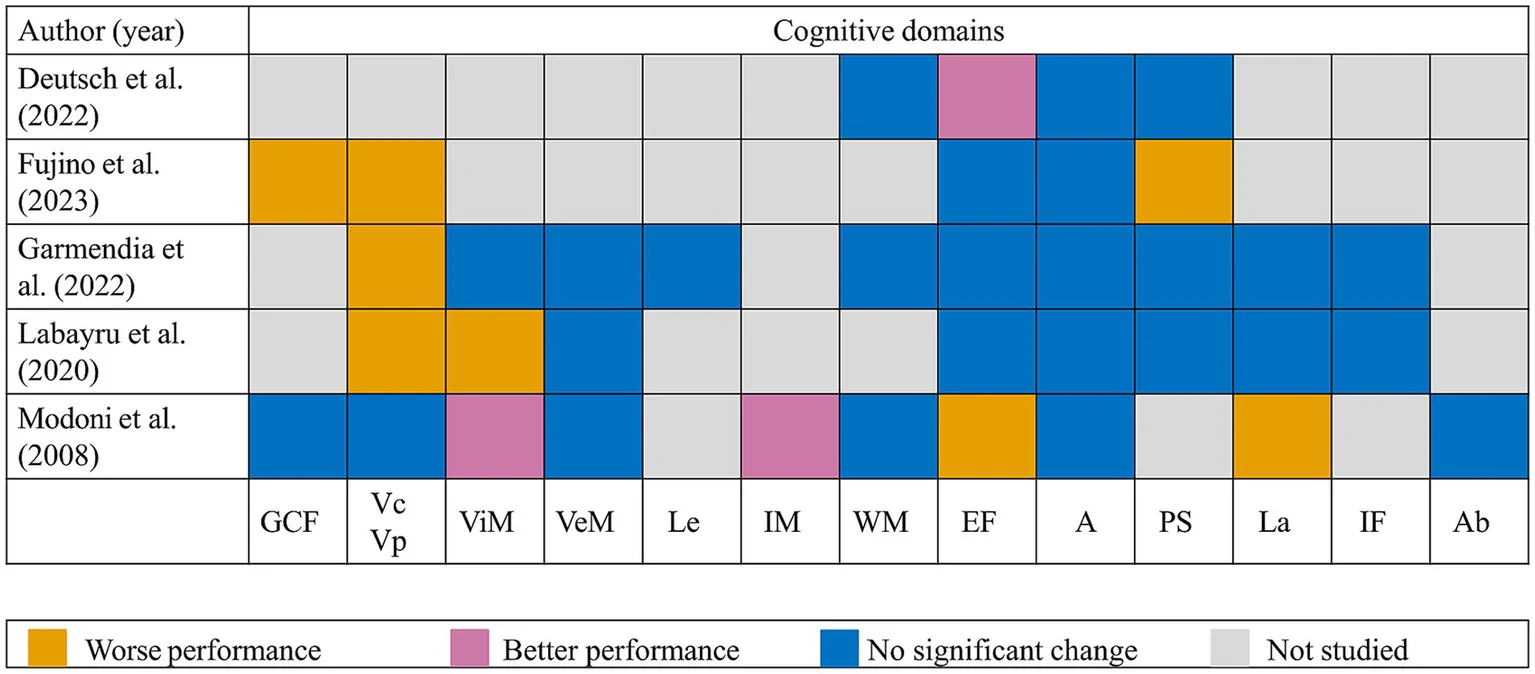
Results of studies reporting cognitive performance over time in DM1 as a single group. “No significant change” indicates that numerical variation did not reach the statistical threshold and should not be interpreted as evidence of clinical or phenotypic stability over time. Furthermore, instances of “better performance” should be interpreted with caution, as longitudinal improvements may reflect retest, familiarity, or practice effects rather than true cognitive recovery. GCF, general cognitive functioning; Vc/Vp, visuoconstruction/perception; ViM, visual memory; VeM, verbal memory; Le, learning; IM, immediate memory; WM, working memory; EF, executive functioning; A, attention; PS, processing speed; La, language; IF, intellectual functioning; Ab, abstract reasoning.

Regarding longitudinal evolution, across follow-up periods ranging from 1 (53) to 11 years (51), DM1 appeared to be characterized by slow, domain-dependent cognitive decline. Four of the five studies reported progressive worsening over time in the visuoconstrutive domain (39, 40, 51, 52). Additional statistically significant declines were observed in processing speed (39, 40), attention (40), visual memory (51), language, and executive functions (52). Notably, these four studies had follow-up periods longer than 4 years. In contrast, the study with the shortest follow-up reported an improvement in executive functions after 3 months, which remained stable at 1 year (53). The results for each study are presented in Figure 4 and weighted consensus for cognitive decline is summarized in Figure 1.

#### 3.4.4 Longitudinal comparisons between DM1 phenotypes

Only four articles analyzed differences in cognitive decline among DM1 phenotypes. Gallais et al. (48) reported that the rate of decline appeared to be greater in late-onset than in adult-onset patients, specifically in semantic categorization, verbal memory, and logical reasoning. However, at follow-up, late-onset patients still obtained higher scores than adult-onset patients (48). Fujino et al. (39) found a greater decline in executive functioning over time in pediatric patients compared with an adult-onset group (*p* = 0.044). Modoni et al. (52), who reported results according to CTG repeat length, found worsening performance in executive and verbal tasks in both comparison groups. However, different cognitive trajectories were observed between groups: the group with 150–500 CTG repeats showed significant overall worsening, except in visual memory, whereas the group with 500–1,000 CTG repeats showed improved performance, except in executive tasks (52). Garmendia, et al. (40) reported significantly poorer performance in executive functions, attention/processing speed, visuoconstructive abilities, and intellectual functioning in manifest patients compared with premanifest individuals. In contrast, no statistically significant differences were found between premanifest individuals, defined as patients in the precursory phase before clinical manifestation of the disease, and healthy controls. However, moderate effect sizes were observed for executive functions and attention/processing speed (40). Longitudinally, however, manifest and premanifest groups showed similar trajectories, with no statistically significant differences in cognitive evolution over time (40).

#### 3.4.5 Correlates of cognitive decline over time

The relationship between cognitive decline and other clinical manifestations of DM1 suggests that CNS involvement follows a trajectory primarily influenced by chronological and disease-related variables. In adult populations, chronological age and disease duration emerged as the most consistent predictors, where advanced age and longer disease duration are directly associated with decline across multiple cognitive domains (39, 41, 47, 48). Moreover, the baseline neuropsychological profile demonstrated consistent prognostic value, as lower initial performance was associated with a higher likelihood of mortality or future functional clinical deterioration (39). Some studies also proposed specific age thresholds at which cognitive performance begins to worsen. Specifically, Fujino et al. (39) reported that executive functioning declines from age 40, whereas Labayru et al. (51) estimated that a more pronounced cognitive decline in DM1 begins around age 31.

Conversely, findings regarding the genetic substrate were inconsistent. Although some studies reported that larger CTG repeat expansions were moderately associated with poorer executive functioning (53), other long-term longitudinal studies found no significant correlations between repeat length and the rate of cognitive decline (48, 52). Likewise, cognitive impairment appeared to be largely independent of motor deterioration (41, 48, 53), except in tasks with high visuomotor demands, where muscular impairment was a significant predictor of decline in complex figure copying (51).

In pediatric populations, cognitive progression followed a different pattern due to its close link with developmental processes. Predictors of negative change in standardized scores included younger age and more severe baseline intellectual disability, as these groups showed the greatest widening of the gap relative to healthy peers over time (43).

Regarding neuroimaging evidence, significant associations were reported between cognitive decline and structural brain correlates. Specifically, reduced white matter integrity (measured using fractional anisotropy) was directly linked to visuoconstructive performance and estimated IQ at long-term follow-up (27). Notably, these structural changes may precede clinical onset, as Garmendia et al. (40) found that premanifest individuals already exhibited early brain vulnerability, including statistically significant white matter lesions and a moderate effect size of reduced gray matter volume. Furthermore, this progressive cognitive deterioration was often accompanied by comorbid clinical manifestations, such as declining sleep quality, increased fatigue, and excessive daytime sleepiness (49).

## 4 Discussion

### 4.1 Longitudinal cognitive profile in DM1

To the best of our knowledge, this systematic review represents the first comprehensive synthesis of longitudinal studies examining cognitive decline in individuals with DM1. Overall, the findings confirm the heterogeneous neuropsychological profile associated with DM1, which varies across phenotypes and is characterized by generally lower cognitive performance, affecting both global intellectual functioning and specific cognitive domains. The longitudinal evidence synthesized in this systematic review provides partial support for the two main hypotheses proposed to explain CNS involvement in DM1. On the one hand, studies including adult- and/or late-onset patients reported findings consistent with domain-specific cognitive decline over time, supporting a selective neurodegenerative hypothesis. On the other hand, findings from pediatric phenotypes, in which symptoms emerge earlier in life, support a neurodevelopmental interpretation. In these patients, most studies did not show further deterioration in cognitive functioning over time, despite more severe cognitive impairment being evident at baseline.

### 4.2 Developmental trajectories in pediatric phenotypes

Congenital and childhood-onset phenotypes are primarily characterized by intellectual disability and global cognitive difficulties, rather than by domain-specific impairment. Regarding developmental trajectories, the evidence suggests that, although affected children do not necessarily lose previously acquired skills, their rate of cognitive development is markedly slower than that of typically developing peers, resulting in a widening developmental gap over time. Although these general patterns suggest relative stability, since no statistically significant changes were found, some included studies reported a specific decline in language abilities over time, indicating that cognitive trajectories in childhood-onset DM1 may not be uniformly stable across all domains. These equivocal findings are consistent with a recent scoping review, which concluded that evidence of cognitive decline in childhood-onset DM1 remains inconclusive. Some studies reported declines in IQ, while others found no statistically significant changes (54). Collectively, these results suggest that the trajectory of cognitive change in congenital and childhood-onset DM1 does not follow the same pattern as that observed in adult and late-onset phenotypes. For juvenile-onset patients, the available data remain scarce, and few studies have provided phenotype-specific information. Accordingly, future research is warranted to clarify whether and how the pattern of cognitive impairment evolves within this subgroup.

### 4.3 Domain-specific decline in adults with DM1

In studies analyzing DM1 as a single group, as well as in those focusing specifically on adult- and late-onset phenotypes, the neuropsychological profile was characterized by globally reduced cognitive performance, with particularly marked involvement of visuoconstructive and visuospatial abilities, and, to a lesser extent, executive function and alternating attention. Although premanifest individuals did not consistently differ from controls at the statistical level, moderate effect sizes and neuroimaging findings suggest subtle early central nervous system (CNS) involvement. Cognitive progression in adult- and late-onset DM1 has been described as a slow but steady decline affecting specific domains, consistent with an accelerated aging-like process. This characterization aligns with prior cross-sectional findings (14, 15). Although some smaller-scale studies suggest no significant changes over time, more extensive longitudinal investigations indicate that most patients exhibit measurable decline over periods of 5 to 11 years.

Visuoconstructive and attentional skills emerged as the domains most susceptible to longitudinal deterioration, corroborating cross-sectional evidence that positions visuoconstruction as a cognitive hallmark of DM1 (12, 13). In contrast, although executive functions appear to be affected in the early stages, several studies indicate that their rate of decline may be slower or plateau over time. Furthermore, one included study reported improvements in executive function at a three-month follow-up, which remained stable at one year. This improvement, however, may be attributable to test–retest learning effects.

Notably, late-onset DM1 appears to follow a faster trajectory of cognitive decline than adult-onset disease. This is particularly relevant given that late-onset patients are generally older at both disease onset and assessment, although this does not necessarily translate into uniformly poorer performance than that observed in adult-onset patients. These findings suggest that late-onset DM1 may be characterized not by greater cognitive severity but by a steeper trajectory of decline, potentially reflecting an interaction between disease progression and advanced age.

### 4.4 Neurodevelopmental vs. neurodegenerative mechanisms

The present findings provide support for both considered hypotheses around cognitive impairment in DM1: the neurodegenerative (12, 14–16) and the neurodevelopmental (28–32, 45). Moreover, the differential profiles observed across phenotypes suggest a plausible possibility of a hybrid model in which early neurodevelopmental vulnerability may interact with later neurodegenerative mechanisms. Accordingly, although methodologically challenging in a rare disease such as DM1, an adequately powered longitudinal study that collects longitudinal data across the lifespan and across all phenotypes would represent the optimal approach to clarifying this issue. It would also be of considerable scientific value to examine cognitive functioning in adult- and late-onset patients during the premanifest stage, before the emergence of classic muscular symptoms, to further elucidate the mechanisms underlying early CNS involvement across the lifespan.

### 4.5 Predictors of longitudinal cognitive changes

Regarding predictors of cognitive deterioration, current age, disease duration, and age of symptom onset emerged as the most robust variables. Baseline characteristics and phenotype also contributed, although to a lesser extent, whereas CTG repeat expansion size did not appear to be a reliable predictor of longitudinal decline. Only one study found an association between physical disability and cognitive dysfunction over time, and this association was limited to a drawing task that involved fine motor skills. Current age was the most consistent and significant predictor of decline across multiple cognitive domains. This may partly explain why studies including younger participants often fail to detect longitudinal cognitive decline.

Although the age of onset and phenotype are relevant predictors of cognitive impairment, and despite the known association between CTG repeat expansion length and disease severity, genetic load does not appear to be as strong an explanatory factor for longitudinal cognitive decline as age at assessment. This dissociation suggests that while genetic burden may determine overall disease severity, epigenetic mechanisms may provide greater explanatory power for the progression of CNS symptomatology in DM1 (55). Furthermore, a deeper understanding of brain involvement and other multisystem manifestations is needed, given that expression of the DMPK mutation secondarily affects the transcriptional processes of other proteins (55).

Some of the reviewed studies support the interpretation that cognitive decline in DM1 is a primary CNS manifestation underpinned by progressive structural brain changes. Similar structural alterations and their association with cognitive decline have been documented in previous studies, which conclude that extensive and progressive involvement of white and grey matter consistently correlates with cognitive deficits (21, 56, 57). Critically, the detection of these brain vulnerabilities in premanifest individuals suggests that neuropathological involvement may precede the onset of classic muscular symptoms. This broader concept that CNS involvement extends beyond the primary muscular phenotype and can follow a distinct clinical trajectory is well-documented across other genetic myopathies. For instance, in Duchenne muscular dystrophy, cognitive deficits remain non-progressive despite ongoing, severe clinical slope (58). This dissociation is further supported by the review of Reimann and Kornblum (17), which highlights that central manifestations occur independently of peripheral motor decline in myotonic dystrophy type 2. According to this review, a similar independence is observed in LAMA2-related muscular dystrophies, where white matter abnormalities typically remain stable during adulthood regardless of the myopathy’s progression. Furthermore, in adult patients with FKRP-related dystroglycanopathies, mild executive and visuospatial deficits have been observed that do not follow a progressive course, while facioscapulohumeral muscular dystrophy, brain white matter changes occur without correlation to muscular disease severity. Expanding beyond primary myopathies, this framework aligns with recent conceptual developments in amyotrophic lateral sclerosis (ALS) (59), where early or subtle cognitive impairment operates on a distinct trajectory that serves as an independent prognostic marker for overall disease outcome.

### 4.6 Limitations

Nevertheless, several limitations should be acknowledged. Characterizing cognitive impairment over time in DM1 is inherently complex, and this posed a challenge throughout this review. Therefore, the conclusions should be interpreted with caution, as the included studies varied in follow-up duration, neuropsychological assessment protocols, and phenotypic composition.

First, it is worth highlighting the significant methodological heterogeneity regarding neuropsychological research in DM1. The included studies varied widely in follow-up duration, ranging from 1 to 20 years. Because cognitive decline in DM1 is typically slow and subtle, shorter follow-ups are inherently more likely to capture stability or practice effects, whereas only long-term cohorts can reliably detect progressive deterioration. This temporal asymmetry limits direct comparisons and precludes the establishment of a unified rate of decline for the disease. Furthermore, the extensive diversity of neuropsychological assessment protocols heavily influences the consistency of the reported patterns. Many utilized tests are not pure-domain measures and intercept multiple cognitive functions simultaneously. For instance, evaluating the same domains with different tests can yield contradictory results due to this interception of other domains. This psychometric variability may explain why some cohorts detect decline while others are not able to capture changes in some domains.

Improvement (48–50, 52, 53) or stability in results of specific domains have been reported in some studies, however, these results may be influenced by familiarity and practice effects that are inherent to longitudinal assessment. Moreover, learning and practice effects have been described and discussed in previous DM1 longitudinal studies (48, 52, 53, 60). Therefore, an actual decline or stability of the cognitive performance could be masked in some cases, as particularly mentioned in some of the included study sample (39, 41), which should be borne in mind when interpreting results.

It should also be noted that methodological quality was primarily constrained by a widespread lack of confounding control and cohort comparability. Specifically, few studies included a healthy control group, limiting the possibility of directly comparing cognitive progression in patients with DM1 with that of the general population. Additionally, given the rarity of DM1, individual study samples were typically small, with substantial heterogeneity in sample size across studies. Nonetheless, by incorporating all available longitudinal studies to date, this systematic review provides a substantially larger aggregate sample than any single primary study.

Additionally, the synthesis of the exact magnitude of cognitive decline was constrained by the widespread lack of effect size reporting in the primary literature. This missing data precluded a rigorous comparison of effect magnitudes across domains, restricting the synthesis primarily to direction and statistical significance. Because statistical significance heavily depends on sample size, small studies with genuine clinical declines may have been undervalued, while findings from larger cohorts naturally dominate the consensus. While an attempt was made to mitigate this imbalance through a visually weighted consensus model, such a qualitative approach cannot fully substitute for a quantitative meta-analysis, which currently remains unfeasible due to the methodological fragmentation of the field.

Furthermore, some research-related limitations must be noted. First, the systematic search was restricted to articles published in English and Spanish. Second, while the search was conducted in three major databases, it did not include other specialized sources such as Embase, nor did it explore grey literature or clinical trial registries. This omission may have limited the identification of additional relevant data or unpublished longitudinal results.

## 5 Conclusion

This systematic review provides evidence of a dual-layered cognitive profile in DM1: an initial baseline impairment, likely neurodevelopmental in origin, upon which subsequent longitudinal decline develops. It remains to be fully elucidated whether this deterioration represents the long-term sequelae of neurodevelopmental vulnerability or an independent, selective neurodegenerative process. Notably, longitudinal decline appears to be domain-specific, predominantly affecting visuoconstructive functions, especially on adult- and late-onset patients. The singularity of this decline suggests that visuoconstructive performance could serve as a sensitive marker of disease evolution. Consequently, standardized tools assessing this domain should be prioritized in clinical monitoring. However, when considering these measures as potential endpoints for tracking CNS efficacy in future clinical trials, a clear distinction must be made regarding motor demands. Because the cognitive deficit in DM1 is specifically constructional rather than perceptual, shifting toward motor-free visuospatial proxies could have sacrificed endpoint sensitivity. To isolate CNS progression while minimizing the peripheral motor confound highlighted in the literature, future trials should prioritize visuoconstructive instruments with low graphomotor demand, rather than drawing-based tasks, or rigorously adjust for baseline motor disability.

Future research should prioritize investigating cognitive decline trajectories across the full spectrum of DM1 phenotypes, using larger sample sizes and longer follow-up periods, as well as establishing a consensus on a standardized neuropsychological assessment battery. Such efforts would facilitate cross-study comparability, strengthen the cumulative evidence base, and meaningfully contribute to characterizing CNS involvement in DM1, ultimately driving the development of targeted interventions to improve patients’ quality of life.

## Data Availability

The original contributions presented in the study are included in the article and its Supplementary material. Specifically, the comprehensive data extracted from all included studies and used for the qualitative synthesis are fully detailed in Table 2 and Supplementary Table S7, the results of studies reporting cognitive performance over time in DM1 are presented in Figures 3, 4, and the weighted consensus synthesis of longitudinal cognitive decline is illustrated in Figure 1, while the list of excluded studies, along with the reasons for exclusion, is available in Table S6. Methodological frameworks, search strategies, and screening protocols are documented via the database search string in Table 1, the study selection process in Figure 2, the SWiM framework in Supplementary Table S5, the PRISMA checklist in Supplementary Table S1, the PICO framework in Supplementary Table S2, and the inter-rater agreement metrics in Supplementary Table S3. Furthermore, the adapted version of the NOS is detailed in Supplementary Data S1, with the complete risk of bias assessments presented in Supplementary Table S4.
