# Supplementary material for: Cognitive decline over time in myotonic dystrophy type 1: a systematic review of longitudinal studies
Source: Front Neurol. 2026 Sep 10;17:1901436. doi: 10.3389/fneur.2026.1901436 (PMC13600866; doi:10.3389/fneur.2026.1901436)
Supplement: Supplementary file 1 [file Supplementary_file_1.docx]

**Supplementary Material**

**Data S1.** *Adaptation of the Newcastle-Ottawa quality assessment scale and detailed scoring criteria.*

Note: The ratings on this adapted version of the NOS are noted next to each subsection. The star system is changed to a point-based rating. Therefore, the maximum score that can be reached is nineteen points (19p.): a maximum of six points (6p.) for Selection, a maximum of three points (3p.) for Comparability, and a maximum of ten points (10p.) for Outcome.

**Selection (6p.)**

1) Representativeness of the DM1 cohort

a) truly representative of the average of DM1 patients in the community **(3p.)**

b) somewhat representative of the average of DM1 patients in the community **(2p.)**

c) selected group of users e.g., one phenotype of DM1 patients **(1p.)**

d) no description of the derivation of the cohort **(0p.)**

2) Selection of the control cohort *

a) drawn from the same community as the DM1 cohort **(3p.)**

b) drawn from a different source **(2p.)**

c) no description of the derivation of the non-DM1 cohort **(1p.)**

d) no control cohort **(0p.)**

**Comparability (3p.)**

1) Comparability of cohorts on the basis of the design or analysis **

a) study controls for AGE AND/OR PHENOTYPES **(2p.)**

b) study controls for any additional factor LEVEL EDUCATION AND/OR SEX **(1p.)**

c) The study does not control for either main or additional factors. **(0p.)**

**Outcome (10p.)**

1) Assessment of outcome******

a) independent blind assessment **(2p.)**

b) standardized tests **(1p.)**

c) no description **(0p.)**

2) Was the follow-up long enough for outcomes to occur

a) yes (3 years) **(1p.)**

b) no **(0p.)**

3) Adequacy of follow-up of cohorts

a) complete follow-up - all subjects accounted for **(3p.)**

b) subjects lost to follow-up unlikely to introduce bias - small number lost - < 20 % (select an   adequate %) follow-up, or description provided of those lost) **(2p.)**

c) follow up rate > 20% (select an adequate %) and no description of those lost **(1p.)**

d) no statement **(0p.)**

4) Assessment of confounders*******

1. Age
2. Education
3. CTG
4. Disease duration
5. Respiratory dysfunction
6. Dysarthria
7. Motor disabilities
8. Depression and/or anxiety
9. Psychopharmacological medication
10. Apathy
11. Fatigue
12. Daytime sleepiness

**Table S1.** *PRISMA checklist completed.*

| **Section and Topic** | **Item #** | **Checklist item** | **Location where item is reported** |
| --- | --- | --- | --- |
| **TITLE** | | |  |
| Title | 1 | Identify the report as a systematic review. | Title |
| **ABSTRACT** | | |  |
| Abstract | 2 | See the PRISMA 2020 for Abstracts checklist. | Abstract |
| **INTRODUCTION** | | |  |
| Rationale | 3 | Describe the rationale for the review in the context of existing knowledge. | Introduction |
| Objectives | 4 | Provide an explicit statement of the objective(s) or question(s) the review addresses. | Introduction |
| **METHODS** | | |  |
| Eligibility criteria | 5 | Specify the inclusion and exclusion criteria for the review and how studies were grouped for the syntheses. | Method – 2.2. Eligibility criteria, and 2.5. Data synthesis |
| Information sources | 6 | Specify all databases, registers, websites, organisations, reference lists and other sources searched or consulted to identify studies. Specify the date when each source was last searched or consulted. | Method – 2.1. Search strategy and Table 1 |
| Search strategy | 7 | Present the full search strategies for all databases, registers and websites, including any filters and limits used. | Method – 2.1. Search strategy and Table 1 |
| Selection process | 8 | Specify the methods used to decide whether a study met the inclusion criteria of the review, including how many reviewers screened each record and each report retrieved, whether they worked independently, and if applicable, details of automation tools used in the process. | Method – 2.3. Screening and data extraction, Figure 2,and Table S6 |
| Data collection process | 9 | Specify the methods used to collect data from reports, including how many reviewers collected data from each report, whether they worked independently, any processes for obtaining or confirming data from study investigators, and if applicable, details of automation tools used in the process. | Method – 2.3. Screening and data extraction. |
| Data items | 10a | List and define all outcomes for which data were sought. Specify whether all results that were compatible with each outcome domain in each study were sought (e.g. for all measures, time points, analyses), and if not, the methods used to decide which results to collect. | Method – 2.3. Screening and data extraction, 2.5. Data synthesis, and Table 2 |
|  | 10b | List and define all other variables for which data were sought (e.g. participant and intervention characteristics, funding sources). Describe any assumptions made about any missing or unclear information. | Method - 2.3. Screening and data extraction, 2.5. Data synthesis, and Table S7 |
| Study risk of bias assessment | 11 | Specify the methods used to assess risk of bias in the included studies, including details of the tool(s) used, how many reviewers assessed each study and whether they worked independently, and if applicable, details of automation tools used in the process. | Method – 2.4. Risk of bias, Data S1 and Table S4 |
| Effect measures | 12 | Specify for each outcome the effect measure(s) (e.g. risk ratio, mean difference) used in the synthesis or presentation of results. | Method – 2.3. Screening and data extraction, 2.5. Data synthesis, Figure 1, Figure 3 and Figure 4 |
| Synthesis methods | 13a | Describe the processes used to decide which studies were eligible for each synthesis (e.g. tabulating the study intervention characteristics and comparing against the planned groups for each synthesis (item #5)). | Method – 2.5. Data synthesis; Results – 3.1. Literature search and selection, 3.2. Characteristics of included studies; Table 2 and Table S7. |
|  | 13b | Describe any methods required to prepare the data for presentation or synthesis, such as handling of missing summary statistics, or data conversions. | Method – 2.5. Data synthesis; and Results – 3.2 Characteristics of included studies |
|  | 13c | Describe any methods used to tabulate or visually display results of individual studies and syntheses. | Methods – 2.5. Data synthesis, Table 2, Figure 1, Figure 3, and Figure 4 |
|  | 13d | Describe any methods used to synthesize results and provide a rationale for the choice(s). If meta-analysis was performed, describe the model(s), method(s) to identify the presence and extent of statistical heterogeneity, and software package(s) used. | Methods – 2.5. Data synthesis, Table S5 and Figure 1 |
|  | 13e | Describe any methods used to explore possible causes of heterogeneity among study results (e.g. subgroup analysis, meta-regression). | Methods – 2.5. Data synthesis; Results – 3.2 Characteristics of included studies |
|  | 13f | Describe any sensitivity analyses conducted to assess robustness of the synthesized results. | Not applicable |
| Reporting bias assessment | 14 | Describe any methods used to assess risk of bias due to missing results in a synthesis (arising from reporting biases). | Not applicable |
| Certainty assessment | 15 | Describe any methods used to assess certainty (or confidence) in the body of evidence for an outcome. | Not applicable |
| **RESULTS** | | |  |
| Study selection | 16a | Describe the results of the search and selection process, from the number of records identified in the search to the number of studies included in the review, ideally using a flow diagram. | Results – 3.1. Literature search and selection, Figure 2, and Table S6 |
|  | 16b | Cite studies that might appear to meet the inclusion criteria, but which were excluded, and explain why they were excluded. | Results – 3.1 Literature search and selection, Figure 2 and Table S6 |
| Study characteristics | 17 | Cite each included study and present its characteristics. | Results – 3.2. Characteristics of included studies, , Table 2, and Table S7 |
| Risk of bias in studies | 18 | Present assessments of risk of bias for each included study. | Results – 3.3. Methodological quality and risk of bias, and Table S4 |
| Results of individual studies | 19 | For all outcomes, present, for each study: (a) summary statistics for each group (where appropriate) and (b) an effect estimate and its precision (e.g. confidence/credible interval), ideally using structured tables or plots. | Results – 3.4. Synthesis of results, Table 2, Figure 3, Figure 4, and Table S7 |
| Results of syntheses | 20a | For each synthesis, briefly summarise the characteristics and risk of bias among contributing studies. | Results – 3.2 Characteristics of included studies, 3.3. Methodological quality and risk of bias, Table 2, Table S4, and Table S7 |
|  | 20b | Present results of all statistical syntheses conducted. If meta-analysis was done, present for each the summary estimate and its precision (e.g. confidence/credible interval) and measures of statistical heterogeneity. If comparing groups, describe the direction of the effect. | Results – 3.4 Synthesis of results, Table 2, Figure 1, Figure 3 and Figure 4 |
|  | 20c | Present results of all investigations of possible causes of heterogeneity among study results. | Results – 3.4.4 Longitudinal comparisons between DM1 phenotype, and 3.4.5. Correlates of cognitive decline over time |
|  | 20d | Present results of all sensitivity analyses conducted to assess the robustness of the synthesized results. | Not applicable |
| Reporting biases | 21 | Present assessments of risk of bias due to missing results (arising from reporting biases) for each synthesis assessed. | Not applicable |
| Certainty of evidence | 22 | Present assessments of certainty (or confidence) in the body of evidence for each outcome assessed. | Not applicable |
| **DISCUSSION** | | |  |
| Discussion | 23a | Provide a general interpretation of the results in the context of other evidence. | Discussion |
|  | 23b | Discuss any limitations of the evidence included in the review. | Discussion – 4.6. Limitations |
|  | 23c | Discuss any limitations of the review processes used. | Discussion – 4.6. Limitations |
|  | 23d | Discuss implications of the results for practice, policy, and future research. | Discussion – 4.4 Neurodevelopmental vs. Neurodegenerative mechanisms, and 4.7. Conclusion |
| **OTHER INFORMATION** | | |  |
| Registration and protocol | 24a | Provide registration information for the review, including register name and registration number, or state that the review was not registered. | Method (Paragraph 1) |
|  | 24b | Indicate where the review protocol can be accessed, or state that a protocol was not prepared. | Method (Paragraph 1) |
|  | 24c | Describe and explain any amendments to information provided at registration or in the protocol. | Method (Paragraph 1) |
| Support | 25 | Describe sources of financial or non-financial support for the review, and the role of the funders or sponsors in the review. | Conflict of interest, Funding and Acknowledgments |
| Competing interests | 26 | Declare any competing interests of review authors. | Conflict of interest |
| Availability of data, code and other materials | 27 | Report which of the following are publicly available and where they can be found: template data collection forms; data extracted from included studies; data used for all analyses; analytic code; any other materials used in the review. | Data availability statement |

*From:* Page MJ, McKenzie JE, Bossuyt PM, Boutron I, Hoffmann TC, Mulrow CD, et al. The PRISMA 2020 statement: an updated guideline for reporting systematic reviews. BMJ 2021;372:n71. doi: 10.1136/bmj.n71. This work is licensed under CC BY 4.0. To view a copy of this license, visit <https://creativecommons.org/licenses/by/4.0/>

**Table S2.** *PICO question.*

| **Population** | **Intervention** | **Comparison** | **Outcome** | **Study design** |
| --- | --- | --- | --- | --- |
| DM1 patients | - | - | Cognitive performance | Longitudinal |
| (myotonic dystrophy) OR (Steinert*) OR (DM1) OR (DM-1) |  |  | (cognitive) OR (cognition) OR (neurodegeneration) OR (neuropsychology) OR (neuropsychological) | (longitudinal) OR (follow-up) |

**Table S3.** *Inter-rater agreement calculated using Cohen’s Kappa.*

|  | Reviewer 2 (GL): Include | Reviewer 2 (GL): Exclude | Total (Reviewer 1) |
| --- | --- | --- | --- |
| Reviewer 1 (AC): Include | 25 | 0 | 25 |
| Reviewer 1 (AC): Exclude | 3 | 409 | 412 |
| Total (Reviewer 2) | 28 | 409 | 437 |

*Note:* Observed Agreement (*p_o_*): 99.31%; Expected Agreement (*p_e_*): 88.60%; Cohen’s Kappa *(kappa*): 0.939

**Table S4.** *Results of the risk of bias assessment for each included study.*

| Article´s  author (year) | Selection | | Comparability | | Outcomes | | Total (points) |
| --- | --- | --- | --- | --- | --- | --- | --- |
|  | Item: option | Score (points) | Item: option | Score (points) | Item: option | Score (points) |  |
| Deutsch et al. (2022) | 1: b; 2: d | 2+0 |  |  | 1: b; 2: b; 3: b;  4: a+b+c+d+e+f+g+h+k+l | 1+0+2+3 | 8 |
| Díaz-Leiva et al. (2020) | 1: c; 2: d | 1+0 |  |  | 1: a; 2: a; 3: b; 4: b | 3+1+2+1 | 8 |
| Echenne et al., (2008) | 1: c; 2: d | 1+0 | c | 0 | 1: b; 2: a; 3:c; 4: c+e+g | 1+1+1+3 | 7 |
| Fujino et al. (2023) | 1: b; 2: d; | 2+0 | a | 2 | 1: b; 2: a; 3: b;  4: b+c+d+e+f+g+h+j+k+l | 1+1+2+3 | 11 |
| Gallais et al. (2017) | 1: b; 2: d | 2+0 | c | 0 | 1: b; 2: a; 3: b;  4: a+b+c+d+g | 1+1+2+3 | 9 |
| Garmendia et al. (2022) | 1: b; 2: c | 2+1 | a+b | 3 | 1: a+b; 2: a; 3: b;  4: a+b+c+d+g+l | 2+1+1+2+3 | 15 |
| Gliem et al. (2019) | 1: c; 2: c | 1+1 | b | 1 | 1: b; 2: a; 3: b;  4: a+b+c+d+g+h+k+l | 1+1+2+3 | 10 |
| Labayru et al. (2020) | 1: a; 2: a+b | 3+3 | a+b | 3 | 1: a+b; 2: a; 3: b;  4: a+b+c+d+g | 3+1+2+3 | 18 |
| Labayru et al. (2022) | 1: b; 2: a+b | 2+3 | a+b | 3 | 1: a+b; 2: a; 3: b;  4: a+b+c+g | 3+1+2+2 | 16 |
| Lindeblad et al. (2019) | 1: b; 2: d | 2+0 | c | 0 | 1: b; 2: a; 3: b; 4: a+c | 1+1+2+2 | 8 |
| Martinello et al. (1999) | 1: b; 2: d | 2+0 | c | 0 | 1: b; 2: a; 3: a; 4: a+c+e+g | 1+1+3+3 | 10 |
| Modoni et al. (2008) | 1: b; 2: d | 2+0 | c | 0 | 1: b; 2: a; 3: b; 4: a+c | 1+1+2+2 | 8 |
| Ricci et al. (2022) | 1: b; 2: d | 2+0 | c | 0 | 1: b; 2: a; 3: b;  4: a+d+g+h | 1+1+2+3 | 9 |
| Sansone et al. (2007) | 1: c; 2: c | 1+1 | a | 2 | 1: b; 2: a; 3: b; 4: a+b+d | 1+1+2+2 | 10 |
| Tuikka et al. (1993) | 1: b; 2: d | 2+0 | c | 0 | 1: b; 2:a; 3: c; 4: a+b+g | 1+1+1+3 | 8 |
| Winbald et al. (2016) | 1: c; 2: d | 1+0 | c | 0 | 1: b; 2: a; 3: a;  4: a+b+c+d+g | 1+1+3+3 | 9 |

**Table S5.** *Synthesis Without Meta-Analysis (SWiM) items*

| **SWiM reporting item** | **Item description** | **Location where item is reported** |
| --- | --- | --- |
| **Methods** |  |  |
| 1 Grouping studies for synthesis | 1a) Provide a description of, and rationale for, the groups used in the synthesis (eg, grouping of populations, interventions, outcomes, study design) | Method – 2.5. Data synthesis; Results – 3.2. Characteristics of included studies |
|  | 1b) Detail and provide rationale for any changes made subsequent to the protocol in the groups used in the synthesis | Method (Paragraph 1) |
| 2 Describe the standardized metric and transformation methods used | Describe the standardized metric for each outcome. Explain why the metric(s) was chosen and describe any methods used to transform the intervention effects, as reported in the study, to the standardized metric, citing any methodological guidance consulted | Method – 2.5. Data synthesis |
| 3 Describe the synthesis methods | Describe and justify the methods used to synthesise the effects for each outcome when it was not possible to undertake a meta-analysis of effect estimates | Method – 2.5 Data synthesis; and Results – 3.2. Characteristics of included studies |
| 4 Criteria used to prioritise results for summary and synthesis | Where applicable, provide the criteria used, with supporting justification, to select the particular studies, or a particular study, for the main synthesis or to draw conclusions from the synthesis (eg, based on study design, risk of bias assessments, directness in relation to the review question) | Method – 2.3. Screening and data extraction, and 2.5. Data synthesis; Results – 3.1. Literature search and selection, and 3.2. Characteristics of included studies |
| 5 Investigation of heterogeneity in reported effects | State the method(s) used to examine heterogeneity in reported effects when it was not possible to undertake a meta-analysis of effect estimates and its extensions to investigate heterogeneity | Method – 2.5. Data synthesis; Results – 3.2. Characteristics of included studies, 3.4.4. Longitudinal comparisons between DM1 phenotypes, and 3.4.5. Correlates of cognitive decline over time |
| 6 Certainty of evidence | Describe the methods used to assess the certainty of the synthesis findings | Method – 2.4. Risk of bias, and 2.5. Data synthesis; Results – 3.3. Methodological quality and risk of bias |
| 7 Data presentation methods | Describe the graphical and tabular methods used to present the effects (eg, tables, forest plots, harvest plots) | Table 2, Figure 1, Figure 3, Figure 4, Table S4 and Table S7 |
|  | Specify key study characteristics (eg, study design, risk of bias) used to order the studies, in the text and any tables or graphs, clearly referencing the studies included |  |
| **Results** |  |  |
| 8 Reporting results | For each comparison and outcome, provide a description of the synthesized findings and the certainty of the findings. Describe the results in language that is consistent with the question the synthesis addresses, and indicate which studies contribute to the synthesis. | Results, and Table 2 |
| **Discussion** |  |  |
| 9 Limitations of the synthesis | Report the limitations of the synthesis methods used and/or the groupings used in the synthesis and how these effect the conclusions that can be drawn in relation to the original review questions. | Discussion – 4.6. Limitations |

**Table S6.** *Summary of rejected articles and reasons for exclusion.*

| Title | DOI | Reference | Exclusion reason |
| --- | --- | --- | --- |
| No title | Not found | Not found. Authors: Bartolomé, A. R., and Santos, L. S. | No tracking possible |
| Follow-up of clinical and MRI changes in juvenile adult onset DM1 | Not found | Baldanzi, S., Angelini, C., Bevilacqua, F. and Siciliano, G. (2018). Follow-up of clinical and mri changes in juvenile adult onset DM1. *Neurology*, *90*. | Wrong publication type |
| Age of onset, cardiovascular pathologies and genotype affect prognosis of type 1 myotonic dystrophy (DM1): Longitudinal experience from 21 years of a neuromuscular clinic | Not found | Galassi, G., Ariatti, A., Mazzoli, M., Garuti, G., Agnoletto, V., Kaleci, S., Meletti, S. and Nichelli, P. (2019). Age of onset, cardiovascular pathologies and genotype affect prognosis of type 1 myotonic dystrophy (DM1): longitudinal experience from 21 years of a neuromuscular clinic. *European Journal of Neurology*, *26*, 248-248. | No cognitive |
| Structural and functional cns changes in myotonic dystrophy types 1 and 2 | Not found | Day, J. W. (2011). Structural and functional CNS changes in myotonic dystrophy types 1 and 2. | Wrong publication type |
| longitudinal assessment of brain structure and function in myotonic dystrophy | Not found | Nopoulos, P. C. (2019). Longitudinal assessment of brain structure and function in myotonic dystrophy. | Wrong publication type |
| Portrait des habiletés cognitives et de la cognition sociale d’adultes atteints du phénotype infantile de la dystrophie myotonique de type 1 | Not found | Dolbec, J. (2022). Portrait des habiletés cognitives et de la cognition sociale d’adultes atteints du phénotype infantile de la dystrophie myotonique de type 1. | Wrong publication type |
| Progressive frontal lobe impairment in myotonic dystrophy type 1 (DM1) and type 2 (DM2): A longitudinal study | Not found | Meola, G., Sansone, V., Gandossini, S., Milanese, S.D., Cattaneo, E., Brescia, A., Cotticelli, B., Zanetti, O., Calabria, M., Cotelli, M. and Binetti, G. (2005). Progressive frontal lobe impairment in myotonic dystrophy type 1 (DM1) and type 2 (DM2): A longitudinal study. *Neurology*, *64*(6), A415-A415. | Wrong publication type |
| Cognitive profile of patients with glycogen storage disease type III: A clinical description of seven cases | 10.1007/s10545-014-9789-1 | Michon, C. and Gargiulo, M. and Hahn-Barma, V. and Petit, F. and Nadaj-Pakleza, A. and Herson, A. and Eymard, B. and Labrune, P. and Laforet, P. (2015). Cognitive profile of patients with glycogen storage disease type III: a clinical description of seven cases. *Journal of Inherited Metabolic Disease*, *38*(3), 573-580. https://doi.org/10.1007/s10545-014-9789-1 | No DM1 |
| Descriptive study of a clinical series of myotonic dystrophy type 1 in uls da guarda | Not found | Gomes, D. N. (2018). Descriptive study of a clinical series of myotonic dystrophy type 1 in uls da guarda. | No cognitive, Wrong publication type |
| Longitudinal assessment of cognitive-behavioral deficits produced by the fragile-x mutation | 10.1016/S0074-7750(08)60281-5 | Fisch, G. S. (1997). Longitudinal assessment of cognitive-behavioral deficits produced by the fragile-x mutation. *International Review of Research in Mental Retardation*, *21*, 221-247. https://doi.org/10.1016/S0074-7750(08)60281-5 | No DM1 |
| Effect of aerobic exercise training and cognitive behavioural therapy on reduction of chronic fatigue in patients with facioscapulohumeral dystrophy: protocol of the facts-2-fshd trial | 10.1186/1471-2377-10-56 | Voet, N. B. M., Bleijenberg, G., Padberg, G. W, van Engelen, B. G. M. and Geurts, A. C. H. (2010). Effect of aerobic exercise training and cognitive behavioural therapy on reduction of chronic fatigue in patients with facioscapulohumeral dystrophy: Protocol of the facts-2-fshd trial. *BMC neurology*, *10*. https://doi.org/10.1186/1471-2377-10-56 | No DM1 |
| Two children with muscular dystrophies ascertained due to referral for diagnosis of autism | 10.1023/A:1022947728569 | Zwaigenbaum, L. and Tarnopolsky, M. (2003). Two children with muscular dystrophies ascertained due to referral for diagnosis of autism. *Journal of Autism and Developmental Disorders*, *33*(2), 193-199. https://doi.org/10.1023/A:1022947728569 | Wrong publication type |
| neuropsychological deficits, structural brain changes and excessive daytime somnolence in myotonic dystrophy type 1 | Not found | Hamilton, M. J. (2019). Neuropsychological deficits, structural brain changes and excessive daytime somnolence in myotonic dystrophy type 1. | Wrong publication type |
| Associations between accelerometer-measured circadian rest-activity rhythm, brain structural and genetic mechanisms, and dementia | 10.1111/pcn.13671 | Liu, Y., Feng, H., Du, J., Yang, L., Xue, H., Zhang, J., Liang, Y. Y. and Liu, Y. (2024). Associations between accelerometer-measured circadian rest-activity rhythm, brain structural and genetic mechanisms, and dementia. *Psychiatry and Clinical Neurosciences*, *78*(7), 393-404. https://doi.org/10.1111/pcn.13671 | No DM1, Wrong publication type |
| Longitudinal evaluation of disease progression in congenital myotonic dystrophy | Not found | Johnson, N., McIntyre, M., Dixon, M., Crockett, B., Bounsanga, J., Hung, M., and Campbell, C. (2018). Longitudinal evaluation of disease progression in congenital myotonic dystrophy. *Neurology*, 90. | Wrong publication type |
| Diabetes mellitus as a model of psychosomatic and somatopsychic interrelationships | 10.1017/S1138741600005990 | Wasserman, L. I. and Trifonova, E. A.(2006). Diabetes mellitus as a model of psychosomatic and somatopsychic interrelationships. *Spanish Journal of Psychology*, *9*(1), 75-85. https://doi.org/10.1017/S1138741600005990 | No DM1, Wrong publication type |
| Brain structure and clinical endpoints in myotonic dystrophy type 2 | Not found | Puwanant, A.(2023). Brain structure and clinical endpoints in myotonic dystrophy type 2. | No DM1 |
| Health endpoints and longitudinal progression in congenital myotonic dystrophy (help-cdm) | Not found | Johnson, N. E.(2019). Health endpoints and longitudinal progression in congenital myotonic dystrophy (help-cdm). | No cognitive |
| Structural and functional cms changes in children with myotonic dystrophy | Not found | Day, J. W.(2011). Structural and functional cms changes in children with myotonic dystrophy. | No cognitive |
| Prospective study in myotonic dystrophy to determine extracellular rna biomarkers | Not found | Wheeler, T. M. (2023). Prospective study in myotonic dystrophy to determine extracellular rna biomarkers. | No cognitive |
| Longitudinal microvascular and neuronal retinal evaluation in patients with diabetes mellitus types 1 and 2 and good glycemic control | 10.1097/IAE.0000000000003880 | Vujosevic, S., Toma, C., Villani, E., Nucci, P., Brambilla, M., Torti, E., Leporati, F., and De Cilla, S. (2023). Longitudinal microvascular and neuronal retinal evaluation in patients with diabetes mellitus types 1 and 2 and good glycemic control. *Retina-The Journal of Retinal and Vitreous Diseases*, *43*(10), 1723-1731. https://doi.org/10.1097/IAE.0000000000003880 | No DM1 |
| Excessive daytime sleepiness and sleep disturbances in patients with neurological diseases - epidemiology and management | 10.2165/00003495-200363240-00003 | Happe, S(2003). Excessive daytime sleepiness and sleep disturbances in patients with neurological diseases - epidemiology and management. *Drugs*, *63*(24), 2725-2737. https://doi.org/10.2165/00003495-200363240-00003 | No DM1, No cognitive |
| Huntington disease-like 2: insight into neurodegeneration from an african disease | 10.1038/s41582-023-00906-y | Krause, A., Anderson, D. G., Ferreira-Correia, A., Dawson, J., Baine-Savanhu, F., and Li, P. P., and Margolis, R. L.(2024). Huntington disease-like 2: Insight into neurodegeneration from an African disease. *Nature Reviews Neurology*, *20*(1), 36-49. https://doi.org/10.1038/s41582-023-00906-y | No DM1 |
| A multi-modal remote monitoring platform for frontotemporal lobar degeneration syndromes | Not found | Alexander Y. P., Vaziri, A., and Anne-Marie, W.(2022). A multi-modal remote monitoring platform for frontotemporal lobar degeneration syndromes. | No DM1 |
| Polysomnographic findings of myotonic dystrophy type 1/type 2: evidence from case–control studies | 10.1093/sleep/zsad280 | Zhang, Y., Ren, R., Yang, L., Jin, H., Nie, Y., Zhang, H., Shi, Y., Sanford, L.D., Vitiello, M.V., and Tang, X.(2024). Polysomnographic findings of myotonic dystrophy type 1/type 2: Evidence from case–control studies. *Sleep*, *47*(1). https://doi.org/10.1093/sleep/zsad280 | No cognitive, Wrong publication type |
| Cognitive function, behaviour and quality of life in children with myotonic dystrophy type 1 in south - eastern norway | 10.1016/j.ejpn.2023.05.004 | Aden, P., Skarbø, A. B., Wallace, S., Ørstavik, K., and Rasmussen, M. (2023). Cognitive function, behaviour and quality of life in children with myotonic dystrophy type 1 in South-Eastern Norway. *European Journal of Paediatric Neurology*, *45*, 1-6. https://doi.org/10.1016/j.ejpn.2023.05.004 | Wrong publication type |
| Different neuropsychological and brain volumetric profiles in a pair of identical twins with myotonic dystrophy type 1 indicate a non-genetic modulation of clinical phenotype | 10.1016/j.nmd.2024.04.007 | Serra, L., Petrucci, A., Bruschini, M., Botta, A., Campisi, C., Caltagirone, C., and Bozzali, M. (2024). Different neuropsychological and brain volumetric profiles in a pair of identical twins with myotonic dystrophy type 1 indicate a non-genetic modulation of clinical phenotype. *Neuromuscular Disorders*, *40*, 24-30. https://doi.org/10.1016/j.nmd.2024.04.007 | Wrong publication type |
| Targeting myotonic dystrophy type 1 with metformin | 10.3390/ijms23052901 | García-Puga, M., Saenz-Antoñanzas, A., Matheu, A., and de Munain, A. L. (2022). Targeting myotonic dystrophy type 1 with metformin. *International Journal of Molecular Sciences*, *23*(5), . https://doi.org/10.3390/ijms23052901 | Wrong publication type |
| Explanatory factors of dynamic balance impairment in myotonic dystrophy type 1 | 10.1002/mus.27527 | Bélair, N., Côté, I., Gagnon, C., Mathieu, J., and Duchesne, E. (2022). Explanatory factors of dynamic balance impairment in myotonic dystrophy type 1. *Muscle and Nerve*, *65*(6), 683-687. https://doi.org/10.1002/mus.27527 | No cognitive |
| Myotonic dystrophy type 1: a comparison between the adult- and late-onset subtype | 10.1002/mus.27766 | Joosten, I.B.T., Horlings, C.G.C., Vosse, B.A.H., Wagner, A., Bovenkerk, D.S.H., Evertz, R., Vernooy, K., van Engelen, B.G.M., and Faber, C.G.(2023). Myotonic dystrophy type 1: A comparison between the adult- and late-onset subtype. *Muscle and Nerve*, *67*(2), 130-137. https://doi.org/10.1002/mus.27766 | No cognitive |
| Quality of life and subjective symptom impact in japanese patients with myotonic dystrophy type 1 | 10.1186/s12883-022-02581-w | Fujino, H., Saito, T., Takahashi, M.P., Takada, H., Nakayama, T., Imura, O., and Matsumura, T. (2022). Quality of life and subjective symptom impact in Japanese patients with myotonic dystrophy type 1. *BMC Neurology*, *22*(1). https://doi.org/10.1186/s12883-022-02581-w | No cognitive, Wrong publication type |
| Speech and language abnormalities in myotonic dystrophy: an overview | 10.1016/j.jocn.2021.10.031 | Hanoun, S., Sun, Y., Ebrahimi, F., and Ghasemi, M. (2022). Speech and language abnormalities in myotonic dystrophy: an overview. *Journal of Clinical Neuroscience*, *96*, 212-220. https://doi.org/10.1016/j.jocn.2021.10.031 | Wrong publication type |
| Overview of the complex relationship between epigenetics markers, CTG repeat instability and symptoms in myotonic dystrophy type 1 | 10.3390/ijms23073477 | de Pontual, L., and Tomé, S. (2022). Overview of the complex relationship between epigenetics markers, CTG repeat instability and symptoms in myotonic dystrophy type 1. *International Journal of Molecular Sciences*, *23*(7). https://doi.org/10.3390/ijms23073477 | No cognitive, Wrong population |
| Phase I study and cell-free dna analysis of t-DM1 and metronomic temozolomide for secondary prevention of her2-positive breast cancer brain metastases | 10.1158/1078-0432.CCR-22-0855 | Jenkins, S., Zhang, W., Steinberg, S.M., Nousome, D., Houston, N., Wu, X., Armstrong, T.S., Burton, E., Smart, D.D., Shah, R., Peer, C.J., Mozarsky, B., Arisa, O., Figg, W.D., Mendoza, T.R., Vera, E., Brastianos, P., Carter, S., Gilbert, M.R., Anders, C.K., Connolly, R.M., Tweed, C., Smith, K.L., Khan, I., Lipkowitz, S., Steeg, P.S., and Zimmer, A.S. (2023). Phase I study and cell-free DNA analysis of t-DM1 and metronomic temozolomide for secondary prevention of her2-positive breast cancer brain metastases. *Clinical Cancer Research*, *29*(8), 1450-1459. https://doi.org/10.1158/1078-0432.CCR-22-0855 | No cognitive, Wrong publication type |
| Clinical characteristics of different forms of myotonic dystrophy type 1 | 10.17650/2073-8803-2023-18-1-22-37 | Erokhina, E.K., Melnik, E.A., and Vlodavets, D.V. (2023). Clinical characteristics of different forms of myotonic dystrophy type 1. *Russkii Zhunal Detskoi Nevrologii*, *18*(1), 22-37. https://doi.org/10.17650/2073-8803-2023-18-1-22-37 | Wrong publication type |
| neurobehavioral phenotype of children with congenital myotonic dystrophy | 10.1212/WNL.0000000000208115 | Patel, N., Berggren, K.N., Hung, M., Bates, K., Dixon, M.M., Bax, K., Adams, H., Butterfield, R.J., Campbell, C., and Johnson, N.E. (2024). Neurobehavioral phenotype of children with congenital myotonic dystrophy. *Neurology*, *102*(5), . https://doi.org/10.1212/WNL.0000000000208115 | Wrong study design |
| A retrospective study of needs and interventions among patients with myotonic dystrophy type 1 in Denmark referred to rehabilitation in the period 2000–2019 | 10.1111/jep.13706 | Allergodt, K., Handberg, C., Frost, A., and Werlauff, U.(2023). A retrospective study of needs and interventions among patients with myotonic dystrophy type 1 in Denmark referred to rehabilitation in the period 2000–2019. *Journal of Evaluation in Clinical Practice*, *29*(1), 39-48. https://doi.org/10.1111/jep.13706 | No cognitive |
| pharmacotherapy alleviates pathological changes in human direct reprogrammed neuronal cell model of myotonic dystrophy type 1 | 10.1371/journal.pone.0269683 | Eltahir, M.K., Nakamori, M., Hattori, S., Kimura, T., Mochizuki, H. and Nagano, S. (2022). Pharmacotherapy alleviates pathological changes in human direct reprogrammed neuronal cell model of myotonic dystrophy type 1. *Plos ONE*, *17*(7). https://doi.org/10.1371/journal.pone.0269683 | No cognitive, Wrong study design |
| Cognitive and emotional disturbances in adult patients with myotonic dystrophy type 1 | 10.17650/2222-8721-2024-14-2-12-24 | Erokhina, E.K., Shamtieva, K.V., Melnik, E.A., Kazakov, D.O., Kurbatov, S.A., Pavlikova, E.P., Tikhonova, O.A., Mershina, E.A., Sinitsyn, V.E., and Vlodavets, D.V. (2024). Cognitive and emotional disturbances in adult patients with myotonic dystrophy type 1. *Nervno-Myshechnye Bolezni*, *14*(2), 12-24. https://doi.org/10.17650/2222-8721-2024-14-2-12-24 | Wrong study design |
| Gender effect on onset, prevalence and surgical treatment of cataract in patients with myotonic dystrophy type 1 | 10.36185/2532-1900-N75 | Scutifero, M., Lanza, M., Petillo, R., De Bernardo, M., Passamano, L., Rosa, N., and Politano, L. (2022). Gender effect on onset, prevalence and surgical treatment of cataract in patients with myotonic dystrophy type 1. *Acta Myologica*, *41*(3), 105-110. https://doi.org/10.36185/2532-1900-N75 | No cognitive |
| The current clinical perception of myotonic dystrophy type 2 | 10.1097/WCO.0000000000001186 | Kleefeld, F. and Schoser, B.(2023). The current clinical perception of myotonic dystrophy type 2. *Current Opinion in Neurology*, *36*(5), 474-478. https://doi.org/10.1097/WCO.0000000000001186 | No DM1 |
| Greater cortical thinning and microstructural integrity loss in myotonic dystrophy type 1 compared to myotonic dystrophy type 2 | 10.1007/s00415-024-12511-0 | Krieger, B., Schneider-Gold, C., Genç, E., Güntürkün, O., Prehn, C., Bellenberg, B., and Lukas, C.(2024). Greater cortical thinning and microstructural integrity loss in myotonic dystrophy type 1 compared to myotonic dystrophy type 2. *Journal of Neurology*, *271*(8), 5525-5540. https://doi.org/10.1007/s00415-024-12511-0 | No cognitive, Wrong study design |
| External ophthalmoplegia and brainstem white matter lesions: Presentation of myotonic dystrophy type 1 | 10.1097/NRL.0000000000000438 | Igreja, L., Ribeiro, L., Cardoso, M., Vasconcelos, C. and Santos, E. (2023). External ophthalmoplegia and brainstem white matter lesions: presentation of myotonic dystrophy type 1. *Neurologist*, *28*(1), 54-56. https://doi.org/10.1097/NRL.0000000000000438 | No cognitive, Wrong study design |
| Dynamics and variability of transcriptomic dysregulation in congenital myotonic dystrophy during pediatric development | 10.1093/hmg/ddac254 | Hale, M.A., Bates, K., Provenzano, M., and Johnson, N.E. (2023). Dynamics and variability of transcriptomic dysregulation in congenital myotonic dystrophy during pediatric development. *Human Molecular Genetics*, *32*(9), 1413-1428. https://doi.org/10.1093/hmg/ddac254 | No cognitive |
| Cognitive impairment, neuroimaging abnormalities, and their correlations in myotonic dystrophy: A comprehensive review | 10.3389/fncel.2024.1369332 | Wu, Y., Wei, Q., Lin, J., Shang, H. and Ou, R. (2024). Cognitive impairment, neuroimaging abnormalities, and their correlations in myotonic dystrophy: a comprehensive review. *Frontiers in Cellular Neuroscience*, *18*. https://doi.org/10.3389/fncel.2024.1369332 | Wrong publication type |
| An integrative analysis of dna methylation pattern in myotonic dystrophy type 1 samples reveals a distinct dna methylation profile between tissues and a novel muscle-associated epigenetic dysregulation | 10.3390/biomedicines10061372 | Koehorst, E., Odria, R., Capó, J., Núñez-Manchón, J., Arbex, A., Almendrote, M., Linares-Pardo, I., Benito, D.N., Saez, V., Nascimento, A., Ortez, C., Rubio, M.Á., Díaz-Manera, J., Alonso-Pérez, J., Lucente, G., Rodriguez-Palmero, A., Ramos-Fransi, A., Martínez-Piñeiro, A., Nogales-Gadea, G. and Suelves, M.(2022). An integrative analysis of dna methylation pattern in myotonic dystrophy type 1 samples reveals a distinct dna methylation profile between tissues and a novel muscle-associated epigenetic dysregulation. *Biomedicines*, *10*(6), . https://doi.org/10.3390/biomedicines10061372 | No DM1, Wrong publication type |
| Outcome measures frequently used to assess muscle strength in patients with myotonic dystrophy type 1: a systematic review | 10.1016/j.nmd.2021.09.014 | Mateus, T., Costa, A., Viegas, D., Marques, A., Herdeiro, M.T. and Rebelo, S. (2022). Outcome measures frequently used to assess muscle strength in patients with myotonic dystrophy type 1: A systematic review. *Neuromuscular Disorders*, *32*(2), 99-115. https://doi.org/10.1016/j.nmd.2021.09.014 | No cognitive, Wrong publication type |
| In vivo parieto-occipital white matter metabolism is correlated with visuospatial deficits in adult DM1 patients | 10.3390/diagnostics12102305 | Evangelisti, S., Gramegna, L.L., De Pasqua, S., Rochat, M.J., Morandi, L., Mitolo, M., Bianchini, C., Vornetti, G., Testa, C., Avoni, P., Liguori, R., Lodi, R. and Tonon, C. (2022). In vivo parieto-occipital white matter metabolism is correlated with visuospatial deficits in adult DM1 patients. *Diagnostics*, *12*(10). https://doi.org/10.3390/diagnostics12102305 | Wrong publication type |
| Respiratory function and sleep in children with myotonic dystrophy type 1 | 10.1016/j.nmd.2023.01.008 | Cheminelle, M., Nougues, M.-C., Isapof, A., Aubertin, G., Corvol, H., Beydon, N. and Taytard, J.(2023). Respiratory function and sleep in children with myotonic dystrophy type 1. *Neuromuscular Disorders*, *33*(3), 263-269. https://doi.org/10.1016/j.nmd.2023.01.008 | No cognitive, Wrong publication type |
| In CIS effect of DMPK expanded alleles in myotonic dystrophy type 1 patients carrying variant repeats at 5′ and 3′ ends of the CTG array | 10.3390/ijms241210129 | Visconti, V.V., Macrì, E., D’Apice, M.R., Centofanti, F., Massa, R., Novelli, G. and Botta, A. (2023). In CIS effect of DMPK expanded alleles in myotonic dystrophy type 1 patients carrying variant repeats at 5′ and 3′ ends of the CTG array. *International Journal of Molecular Sciences*, *24*(12), . https://doi.org/10.3390/ijms241210129 | No cognitive, Wrong publication type |
| Sleep disorders and fatigue in patients with different forms of myotonic dystrophy type 1 | 10.1007/s11055-024-01565-2 | Erokhina, E.K., Melnik, E.A., Lebedeva, D.D., Shamtieva, K.V., Peters, T.V., Pavlikova, E.P., Gepard, V.V. and Vlodavets, D.V. (2024). Sleep disorders and fatigue in patients with different forms of myotonic dystrophy type 1. *Neuroscience and Behavioral Physiology*, *54*(1), 35-40. https://doi.org/10.1007/s11055-024-01565-2 | No cognitive |
| Cognitive impairment in myotonic dystrophy type 1 (steinert's disease) | 10.1016/j.semerg.2022.01.013 | Rosado Bartolomé, A., Puertas Martín, V., Domínguez González, C. and Ramos Miranda, M.(2022). Cognitive impairment in myotonic dystrophy type 1 (steinert's disease). *Semergen*, *48*(3), 208-213. https://doi.org/10.1016/j.semerg.2022.01.013 | Wrong publication type |
| Excessive rest time during active phase is reliably detected in a mouse model of myotonic dystrophy type 1 using home cage monitoring | 10.3389/fnbeh.2023.1130055 | Golini, E., Rigamonti, M., Raspa, M., Scavizzi, F., Falcone, G., Gourdon, G. and Mandillo, S.(2023). Excessive rest time during active phase is reliably detected in a mouse model of myotonic dystrophy type 1 using home cage monitoring. *Frontiers in Behavioral Neuroscience*, *17*. https://doi.org/10.3389/fnbeh.2023.1130055 | No cognitive, Wrong publication type |
| Living with adult-onset myotonic dystrophy type 1: A scoping review | 10.1080/09638288.2024.2396063 | Allergodt, K., Dreyer, P., Werlauff, U. and Handberg, C. (2024). Living with adult-onset myotonic dystrophy type 1: A scoping review. *Disability and Rehabilitation*. https://doi.org/10.1080/09638288.2024.2396063 | No cognitive, Wrong publication type |
| A meta-analysis of the prevalence of neuropsychiatric disorders and their association with disease onset in myotonic dystrophy | 10.1017/neu.2024.27 | Pascual-Morena, C., Martínez-Vizcaíno, V., Cavero-Redondo, I., Álvarez-Bueno, C., Lucerón-Lucas-Torres, M., Saz-Lara, A. and Martínez-García, I.(2024). A meta-analysis of the prevalence of neuropsychiatric disorders and their association with disease onset in myotonic dystrophy. *Acta Neuropsychiatrica*, *36*(6), 390-401. https://doi.org/10.1017/neu.2024.27 | No cognitive, Wrong publication type |
| Latent factors underlying the symptoms of adult-onset myotonic dystrophy type 1 during the clinical course | 10.1186/s13023-024-03359-8 | Zhang, Y., Wallace, B., Cai, B., Johnson, N., Ciafaloni, E., Venkatesh, Y.S., Westfield, C. and McDermott, S. (2024). Latent factors underlying the symptoms of adult-onset myotonic dystrophy type 1 during the clinical course. *Orphanet Journal of Rare Diseases*, *19*(1). https://doi.org/10.1186/s13023-024-03359-8 | No cognitive, Wrong publication type |
| Myotonic dystrophy type 1: 13 years of experience at a tertiary hospital. clinical and epidemiological study and genotype-phenotype correlation | 10.1016/j.nrl.2021.02.012 | Sánchez Marín, J.P., Sienes Bailo, P., Lahoz Alonso, R., Capablo Liesa, J.L., Gazulla Abio, J., Giménez Muñoz, J.A., Modrego Pardo, P.J., Pardiñas Barón, B. and Izquierdo Álvarez, S. (2023). Myotonic dystrophy type 1: 13 years of experience at a tertiary hospital. clinical and epidemiological study and genotype-phenotype correlation. *Neurologia*, *38*(8), 530-540. https://doi.org/10.1016/j.nrl.2021.02.012 | No cognitive, Wrong publication type |
| Do classical and computerized cognitive tests have equal intrarater reliability in myotonic dystrophy type 1? | 10.1016/j.nmd.2023.04.008 | Fortin, J., Côté, I., Gagnon, C., and Gallais, B.(2023). Do classical and computerized cognitive tests have equal intrarater reliability in myotonic dystrophy type 1?. *Neuromuscular Disorders*, *33*(6), 490-497. https://doi.org/10.1016/j.nmd.2023.04.008 | No cognitive, Wrong publication type |
| Loss of mbnl1-mediated retrograde BDNF signaling in the myotonic dystrophy brain | 10.1186/s40478-023-01540-x | Wang, P.-Y., Kuo, T.-Y., Wang, L.-H., Liang, W.-H. and Wang, G.-S.(2023). Loss of mbnl1-mediated retrograde BDNF signaling in the myotonic dystrophy brain. *Acta Neuropathologica Communications*, *11*(1), . https://doi.org/10.1186/s40478-023-01540-x | No cognitive, Wrong publication type |
| Gait changes induced by a 6-min walking exercise in individuals with myotonic dystrophy type 1: Relationship with muscle strength | 10.1016/j.clinbiomech.2025.106446 | Hoerter, B., Cherni, Y., Comtois, A.S., Vocos, M., Veilleux, L.-N. and Ballaz, L.(2025). Gait changes induced by a 6-min walking exercise in individuals with myotonic dystrophy type 1: Relationship with muscle strength. *Clinical Biomechanics*, *122*. https://doi.org/10.1016/j.clinbiomech.2025.106446 | No cognitive, Wrong publication type |
| Pharmacological and exercise-induced activation of AMPK as emerging therapies for myotonic dystrophy type 1 patients | 10.1113/JP282725 | Ravel-Chapuis, A., Duchesne, E. and Jasmin, B.J. (2022). Pharmacological and exercise-induced activation of AMPK as emerging therapies for myotonic dystrophy type 1 patients. *Journal of Physiology*, *600*(14), 3249-3264. https://doi.org/10.1113/JP282725 | No cognitive, Wrong publication type |
| Neuropathology of classic myotonic dystrophy type 1 is characterized by both early initiation of primary age-related tauopathy of the hippocampus and unique 3-repeat tauopathy of the brainstem | 10.1093/jnen/nlac097 | Hamasaki, H., Maeda, N., Sasagasako, N., Honda, H., Shijo, M., Mori, S.-I., Yagita, K., Arahata, H. and Iwaki, T. (2023). Neuropathology of classic myotonic dystrophy type 1 is characterized by both early initiation of primary age-related tauopathy of the hippocampus and unique 3-repeat tauopathy of the brainstem. *Journal of Neuropathology and Experimental Neurology*, *82*(1), 29-37. https://doi.org/10.1093/jnen/nlac097 | No cognitive, Wrong publication type |
| Executive dysfunction, social cognition impairment, and gray matter pathology in myotonic dystrophy type 2: A pilot study | 10.1097/WNN.0000000000000314 | Theodosiou, T., Christidi, F., Xirou, S., Karavasilis, E., Bede, P., Papadopoulos, C., Argyropoulos, G.D., Kourtesis, P., Pantolewn, V., Ferentinos, P., Kararizou, E., Velonakis, G., Zalonis, I. and Papadimas, G. (2022). Executive dysfunction, social cognition impairment, and gray matter pathology in myotonic dystrophy type 2: a pilot study. *Cognitive and Behavioral Neurology*, *35*(3), 204-211. https://doi.org/10.1097/WNN.0000000000000314 | No DM1 |
| Transcranial brain parenchyma sonographic findings in patients with myotonic dystrophy type 1 and 2 | 10.1016/j.heliyon.2024.e26856 | Mijajlovic, M., Bozovic, I., Pavlovic, A., Rakocevic-Stojanovic, V., Gluscevic, S., Stojanovic, A., Basta, I., Meola, G. and Peric, S.(2024). Transcranial brain parenchyma sonographic findings in patients with myotonic dystrophy type 1 and 2. *Heliyon*, *10*(5), . https://doi.org/10.1016/j.heliyon.2024.e26856 | No cognitive, Wrong publication type |
| Differences in diffusion tensor imaging parameters of brain white matter tracts between patients with myotonic dystrophy type 1 and type 2 — a retrospective single-centre study | 10.5603/pjnns.95587 | Maj, E., Wolak, T., de Meulder, J., Janiszewska, K., Wojtaszek, M., Kostera-Pruszczyk, A., Gołębiowski, M. and Łusakowska, A.(2023). Differences in diffusion tensor imaging parameters of brain white matter tracts between patients with myotonic dystrophy type 1 and type 2 — a retrospective single-centre study. *Neurologia i Neurochirurgia Polska*, *57*(5), 430-437. https://doi.org/10.5603/pjnns.95587 | No cognitive, Wrong publication type |
| Cardiac manifestations of 6 cases of myotonic dystrophy and literature review | 10.3969/j.issn.1000-3614.2022.07.012 | Xu, L., Wang, F., Feng, X., Zhang, Y., Tang, Y.(2022). Cardiac manifestations of 6 cases of myotonic dystrophy and literature review. *Chinese Circulation Journal*, *37*(7), 726-730. https://doi.org/10.3969/j.issn.1000-3614.2022.07.012 | No cognitive, Wrong publication type |
| The Covid-19 pandemic and its influence on patients with myotonic dystrophy type 1: Lessons learned | 10.1002/brb3.70162 | Kleinveld, V.E.A., Bruijnes, J.E., Labrecque, S., Jeurissen-Bekkering, D.E.G., Faber, C.G. and Horlings, C.G.C. (2024). The covid-19 pandemic and its influence on patients with myotonic dystrophy type 1: Lessons learned. *Brain and Behavior*, *14*(11), . https://doi.org/10.1002/brb3.70162 | No cognitive |
| Cellular senescence and aging in myotonic dystrophy | 10.3390/ijms23042339 | Hasuike, Y., Mochizuki, H. and Nakamori, M. (2022). Cellular senescence and aging in myotonic dystrophy. *International Journal of Molecular Sciences*, *23*(4). https://doi.org/10.3390/ijms23042339 | Wrong publication type |
| Intellectual profile in myotonic dystrophy type 1 and its association with its onset: A systematic review and meta-analysis | 10.1016/j.pediatrneurol.2024.08.002 | Pascual-Morena, C., Cavero-Redondo, I., Saz-Lara, A., Martínez-García, I., Visier-Alfonso, M.E. and Martínez-Vizcaíno, V. (2024). Intellectual profile in myotonic dystrophy type 1 and its association with its onset: A systematic review and meta-analysis. *Pediatric Neurology*, *161*, 9-17. https://doi.org/10.1016/j.pediatrneurol.2024.08.002 | Wrong publication type |
| Prevalence of myotonic dystrophy type 1 in Pinar del Río province, Cuba | 10.56294/sctconf2023493 | Otero, D.L., Castillo, M.O., Barbeito, T.O.T., Padrón, L.S. and Licourt, M.T.(2023). Prevalence of myotonic dystrophy type 1 in Pinar del Río province, Cuba. *Salud, Ciencia y Tecnología - Serie de Conferencias*, 2. https://doi.org/10.56294/sctconf2023493 | No cognitive, Wrong publication type |
| Heterogeneity of cognitive impairments in myotonic dystrophy type 1 explained by three distinct cognitive profiles | 10.1007/s00415-024-12404-2 | Davion, J.-B., Tard, C., Fragoso, L., Wilu-Wilu, A., Skrobala, E., Defebvre, L. and Delbeuck, X. (2024). Heterogeneity of cognitive impairments in myotonic dystrophy type 1 explained by three distinct cognitive profiles. *Journal of Neurology*, *271*(7), 4529-4539. https://doi.org/10.1007/s00415-024-12404-2 | Wrong study design |
| Resistance exercise training rescues mitochondrial dysfunction in skeletal muscle of patients with myotonic dystrophy type 1 | 10.3233/JND-230099 | Di Leo, V., Lawless, C., Roussel, M.-P., Gomes, T.B., Gorman, G.S., Russell, O.M., Tuppen, H.A.L., Duchesne, E. and Vincent, A.E. (2023). Resistance exercise training rescues mitochondrial dysfunction in skeletal muscle of patients with myotonic dystrophy type 1. *Journal of Neuromuscular Diseases*, *10*(6), 1111-1126. https://doi.org/10.3233/JND-230099 | No cognitive |
| New horizons in myotonic dystrophy type 1: Cellular senescence as a therapeutic target | 10.1002/bies.202400216 | Légaré, C., Berglund, J.A., Duchesne, E. and Dumont, N.A.(2025). New horizons in myotonic dystrophy type 1: Cellular senescence as a therapeutic target. *Bioessays*, *47*(3). https://doi.org/10.1002/bies.202400216 | No cognitive |
| Balance impairment in myotonic dystrophy type 1: Dynamic posturography suggests the coexistence of a proprioceptive and vestibular deficit | 10.3389/fnhum.2022.925299 | Scarano, S., Sansone, V.A., Ferrari Aggradi, C.R., Carraro, E., Tesio, L., Amadei, M., Rota, V., Zanolini, A. and Caronni, A. (2022). Balance impairment in myotonic dystrophy type 1: dynamic posturography suggests the coexistence of a proprioceptive and vestibular deficit. *Frontiers in Human Neuroscience*, *16*. https://doi.org/10.3389/fnhum.2022.925299 | No cognitive |
| Molecular pathology of myotonic dystrophy type 1 in iceland | 10.1002/mgg3.70013 | Hallgrímsdóttir, E.G., Svansson, H., Stefánsdóttir, V.F., Sveinsson, Ó.Á., Ólafsdóttir, H., Briem, E., Sveinbjörnsdóttir, S. and Jónsson, J.J. (2024). Molecular pathology of myotonic dystrophy type 1 in iceland. *Molecular Genetics and Genomic Medicine*, *12*(10), . https://doi.org/10.1002/mgg3.70013 | No cognitive |
| Visuoconstructional impairment in DM1: Exploring underlying cognitive processes through the Rey Complex Figure | 10.1080/13803395.2023.2274623 | Garmendia, J., Sistiaga, A., and Labayru, G.(2023). Visuoconstructional impairment in DM1: Exploring underlying cognitive processes through the Rey Complex Figure. *Journal of Clinical and Experimental Neuropsychology*, *45*(6), 597-605. https://doi.org/10.1080/13803395.2023.2274623 | Wrong study design |
| Masseter muscle volume as a disease marker in adult-onset myotonic dystrophy type 1 | 10.1016/j.nmd.2022.09.005 | Oliwa, A., Hocking, C., Hamilton, M.J., McLean, J., Cumming, S., Ballantyne, B., Jampana, R., Longman, C., Monckton, D.G. and Farrugia, M.E. (2022). Masseter muscle volume as a disease marker in adult-onset myotonic dystrophy type 1. *Neuromuscular Disorders*, *32*(11), 893-902. https://doi.org/10.1016/j.nmd.2022.09.005 | No cognitive |
| High resolution analysis of DMPK hypermethylation and repeat interruptions in myotonic dystrophy type 1 | 10.3390/genes13060970 | Rasmussen, A., Hildonen, M., Vissing, J., Duno, M., Tümer, Z. and Birkedal, U. (2022). High resolution analysis of DMPK hypermethylation and repeat interruptions in myotonic dystrophy type 1. *Genes*, *13*(6). https://doi.org/10.3390/genes13060970 | No cognitive |
| In myotonic dystrophy type 1 head repositioning errors suggest impaired cervical proprioception | 10.3390/jcm13164685 | Scarano, S., Caronni, A., Carraro, E., Ferrari Aggradi, C.R., Rota, V., Malloggi, C., Tesio, L. and Sansone, V.A. (2024). In myotonic dystrophy type 1 head repositioning errors suggest impaired cervical proprioception. *Journal of Clinical Medicine*, *13*(16). https://doi.org/10.3390/jcm13164685 | No cognitive |
| Alterations in degree centrality and functional connectivity associated with cognitive impairment in myotonic dystrophy type 1:a preliminary functional mri study | 10.1016/j.neuroscience.2025.02.040 | Qian, S., Di, H., Pei, H., Zeqi, H., Jiaxi, Z., Jun, L., Xize, J., Xiaomeng, X. and Haiyan, Z. (2025). Alterations in degree centrality and functional connectivity associated with cognitive impairment in myotonic dystrophy type 1:a preliminary functional mri study. *Neuroscience*, *572*, 49-55. https://doi.org/10.1016/j.neuroscience.2025.02.040 | Wrong study design |
| Investigating phenotypic variability patterns in myotonic dystrophy type 2 in a neuromuscular referral center retrospective cohort | 10.1016/j.nmd.2024.105255 | Picher-Martel, V., Locascio, J.J., Chuang, K., David, W.S., Amato, A.A. and Gonzalez-Perez, P.(2025). Investigating phenotypic variability patterns in myotonic dystrophy type 2 in a neuromuscular referral center retrospective cohort. *Neuromuscular Disorders*, 47. https://doi.org/10.1016/j.nmd.2024.105255 | No DM1 |
| Instrumental activities of daily living in adults with the DM1 childhood phenotype: Going beyond motor impairments | 10.1016/j.nmd.2022.02.004 | Muslemani, S., Gagnon, C. and Gallais, B. (2022). Instrumental activities of daily living in adults with the DM1 childhood phenotype: Going beyond motor impairments. *Neuromuscular Disorders*, *32*(4), 313-320. https://doi.org/10.1016/j.nmd.2022.02.004 | Wrong study design |
| Use of HSALR female mice as a model for the study of myotonic dystrophy type I | 10.1038/s41684-025-01506-7 | Carrascosa-Sàez, M., Colom-Rodrigo, A., González-Martínez, I., Pérez-Gómez, R., García-Rey, A., Piqueras-Losilla, D., Ballestar, A., Llamusí, B., Cerro-Herreros, E. and Artero, R.(2025). Use of HSALR female mice as a model for the study of myotonic dystrophy type i. *Lab Animal*, *54*(4), 92-102. https://doi.org/10.1038/s41684-025-01506-7 | No cognitive, Wrong study design |
| A validated wais-iv short-form to estimate intellectual functioning in myotonic dystrophy type 1 | 10.1016/j.nmd.2022.06.012 | Garmendia, J., Labayru, G., Aliri, J., Winblad, S., Angeard, N., López de Munain, A. and Sistiaga, A. (2022). A validated WAIS-IV short-form to estimate intellectual functioning in myotonic dystrophy type 1. *Neuromuscular Disorders*, *32*(9), 749-753. https://doi.org/10.1016/j.nmd.2022.06.012 | No cognitive |
| Fluid biomarkers of central nervous system (CNS) involvement in myotonic dystrophy type 1 (DM1) | 10.3390/ijms24032204 | Rossi, S. and Silvestri, G.(2023). Fluid biomarkers of central nervous system (cns) involvement in myotonic dystrophy type 1 (DM1). *International Journal of Molecular Sciences*, *24*(3), . https://doi.org/10.3390/ijms24032204 |  |
| Resistance training in women with myotonic dystrophy type 1: A multisystemic therapeutic avenue | 10.1016/j.nmd.2024.05.009 | Girard-Côté, L., Gallais, B., Gagnon, C., Roussel, M.-P., Morin, M., Hébert, L.J., Monckton, D., Leduc-Gaudet, J.-P., Gouspillou, G., Marcangeli, V. and Duchesne, E. (2024). Resistance training in women with myotonic dystrophy type 1: A multisystemic therapeutic avenue. *Neuromuscular Disorders*, *40*, 38-51. https://doi.org/10.1016/j.nmd.2024.05.009 | No cognitive, Wrong publication type |
| Periostin as a blood biomarker of muscle cell fibrosis, cardiomyopathy and disease severity in myotonic dystrophy type 1 | 10.1007/s00415-023-11633-1 | Nguyen, C.D.L., Jimenez-Moreno, A.C., Merker, M., Bowers, C.J., Nikolenko, N., Hentschel, A., Müntefering, T., Isham, A., Ruck, T., Vorgerd, M., Dobelmann, V., Gourdon, G., Schara-Schmidt, U., Gangfuss, A., Schröder, C., Sickmann, A., Gross, C., Gorman, G., Stenzel, W., Kollipara, L., Hathazi, D., Spendiff, S., Gagnon, C., Preusse, C., Duchesne, E., Lochmüller, H. and Roos, A.(2023). Periostin as a blood biomarker of muscle cell fibrosis, cardiomyopathy and disease severity in myotonic dystrophy type 1. *Journal of Neurology*, *270*(6), 3138-3158. https://doi.org/10.1007/s00415-023-11633-1 | No cognitive, Wrong publication type |
| Myotonic dystrophies | 10.1007/978-3-031-44009-0_3 | Silvestri, G. and Modoni, A.(2023). Myotonic dystrophies. *Current clinical neurology*, 37-61. https://doi.org/10.1007/978-3-031-44009-0_3 | Wrong publication type |
| Cognitive assessment in patients with myotonic dystrophy type 2 | 10.1016/j.nmd.2022.06.011 | Peric, S., Gunjic, I., Delic, N., Stojiljkovic Tamas, O., Salak-Djokic, B., Pesovic, J., Petrovic Djordjevic, I., Ivanovic, V., Savic-Pavicevic, D., Meola, G. and Rakocevic-Stojanovic, V.(2022). Cognitive assessment in patients with myotonic dystrophy type 2. *Neuromuscular Disorders*, *32*(9), 743-748. https://doi.org/10.1016/j.nmd.2022.06.011 | No DM1 |
| Blood-based markers of neuronal injury in adult-onset myotonic dystrophy type 1 | 10.3389/fneur.2021.791065 | van der Plas, E., Long, J.D., Koscik, T.R., Magnotta, V., Monckton, D.G., Cumming, S.A., Gottschalk, A.C., Hefti, M., Gutmann, L. and Nopoulos, P.C. (2022). Blood-based markers of neuronal injury in adult-onset myotonic dystrophy type 1. *Frontiers in Neurology*, 12. https://doi.org/10.3389/fneur.2021.791065 | No cognitive, Wrong publication type |
| Child neurology: Maternal transmission of congenital myotonic dystrophy type 2: case report | 10.1212/WNL.0000000000201427 | Tieleman, A.A., Damen, M.J., Verrips, A., Roelofs, M., Kamsteeg, E.-J. and Voermans, N.C.(2022). Child neurology: Maternal transmission of congenital myotonic dystrophy type 2: Case report. *Neurology*, *99*(24), 1112-1114. https://doi.org/10.1212/WNL.0000000000201427 | No cognitive, Wrong publication type |
| A rare case of a patient with myotonic dystrophy type 1 and multiple sclerosis – a case report | 10.21860/medflum2024_316227 | Prenc, M., Labinac, D.J., Prenc, L.R. and Bradić, M.F. (2024). A rare case of a patient with myotonic dystrophy type 1 and multiple sclerosis – a case report. *Medicina Fluminensis*, *60*(2), 214-219. https://doi.org/10.21860/medflum2024_316227 | Wrong publication type |
| Myotonic dystrophy type 1 presenting with dyspnea: A case report | 10.12998/wjcc.v10.i20.7060 | Jia, Y.-X., Dong, C.-L., Xue, J.-W., Duan, X.-Q., Xu, M.-Y., Su, X.-M. and Li, P.(2022). Myotonic dystrophy type 1 presenting with dyspnea: A case report. *World Journal of Clinical Cases*, *10*(20), 7060-7067. https://doi.org/10.12998/wjcc.v10.i20.7060 | Wrong publication type |
| Association between reported sleep disorders and behavioral issues in children with myotonic dystrophy type 1—results from a retrospective analysis in italy | 10.3390/jcm13185459 | Trucco, F., Lizio, A., Roma, E., di Bari, A., Salmin, F., Albamonte, E., Casiraghi, J., Pozzi, S., Becchiati, S., Antonaci, L., Salvalaggio, A., Catteruccia, M., Tosi, M., Marinella, G., Danti, F.R., Bruschi, F., Veneruso, M., Parravicini, S., Fiorillo, C., Berardinelli, A., Pini, A., Moroni, I., Astrea, G., Battini, R., D’Amico, A., Ricci, F., Pane, M., Mercuri, E.M., Johnson, N.E. and Sansone, V.A.(2024). Association between reported sleep disorders and behavioral issues in children with myotonic dystrophy type 1—results from a retrospective analysis in Italy. *Journal of Clinical Medicine*, *13*(18). https://doi.org/10.3390/jcm13185459 | No cognitive |
| New multidisciplinary approaches to avoid refusal of treatment in steinert disease | 10.33588/rn.7403.2021426 | Ramos-Salvachúa, Á.(2022). New multidisciplinary approaches to avoid refusal of treatment in steinert disease. *Revista de Neurologia*, *74*(3), 105. https://doi.org/10.33588/rn.7403.2021426 | Wrong publication type |
| Gray matter abnormalities in myotonic dystrophy type 1: a voxel-wise meta-analysis | 10.3389/fneur.2022.891789 | Jiang, Q., Lin, J., Li, C., Hou, Y. and Shang, H.(2022). Gray matter abnormalities in myotonic dystrophy type 1: A voxel-wise meta-analysis. *Frontiers in neurology*, 13. https://doi.org/10.3389/fneur.2022.891789 | Wrong publication type |
| Tau positron emission tomography, cerebrospinal fluid and plasma biomarkers of neurodegeneration, and neurocognitive testing: an exploratory study of participants with myotonic dystrophy type 1 | 10.1007/s00415-022-10970-x | Laforce, R.J., Dallaire-Théroux, C., Racine, A.M., Dent, G., Salinas-Valenzuela, C., Poulin, E., Cayer, A.-M., Bédard-Tremblay, D., Rouleau-Bonenfant, T., St-Onge, F., Schraen-Maschke, S., Beauregard, J.-M., Sergeant, N., Puymirat, J.(2022). Tau positron emission tomography, cerebrospinal fluid and plasma biomarkers of neurodegeneration, and neurocognitive testing: An exploratory study of participants with myotonic dystrophy type 1. *Journal of Neurology*, *269*(7), 3579-3587. https://doi.org/10.1007/s00415-022-10970-x | Wrong study design |
| Recent progress and challenges in the development of antisense therapies for myotonic dystrophy type 1 | 10.3390/ijms232113359 | De Serres-Bérard, T., Ait Benichou, S., Jauvin, D., Boutjdir, M., Puymirat, J. and Chahine, M.(2022). Recent progress and challenges in the development of antisense therapies for myotonic dystrophy type 1. *International Journal of Molecular Sciences*, *23*(21), . https://doi.org/10.3390/ijms232113359 | No cognitive, Wrong study design |
| Rest-activity disturbances and light exposure in myotonic dystrophy type 1 patients with apathy: An exploratory study using actigraphy | 10.1016/j.jadr.2023.100637 | Laberge, L., Maltais, A., Turcotte, O., Gagnon, C., Duchesne, E. and Gallais, B. (2023). Rest-activity disturbances and light exposure in myotonic dystrophy type 1 patients with apathy: An exploratory study using actigraphy. *Journal of Affective Disorders Reports*, *14*. https://doi.org/10.1016/j.jadr.2023.100637 | No cognitive, Wrong study design |
| The myotonic dystrophies | 10.1016/B978-0-443-19176-3.00029-7 | Moxley, R.T. and Hilbert, J.E. and Meola, G.(2024). The myotonic dystrophies. Rosenberg's molecular and genetic basis of neurological and psychiatric disease, seventh edition: volume 2, 2, 613-634. https://doi.org/10.1016/B978-0-443-19176-3.00029-7 | No cognitive |
| A novel class of fkbp12 ligands rescues premature aging phenotypes associated with myotonic dystrophy type 1 | 10.3390/cells13231939 | García-Puga, M., Gerenu, G., Bargiela, A., Espinosa-Espinosa, J., Mosqueira-Martín, L., Sagartzazu-Aizpurua, M., Aizpurua, J.M., Vallejo-Illarramendi, A., Artero, R., López de Munain, A. and Matheu, A. (2024). A novel class of fkbp12 ligands rescues premature aging phenotypes associated with myotonic dystrophy type 1. *Cells*, *13*(23). https://doi.org/10.3390/cells13231939 | No cognitive |
| Update on therapy for myotonic dystrophy type 1 | 10.1007/s11940-023-00760-9 | Ivanovic, V., Meola, G., Vukojevic, Z. and Peric, S. (2023). Update on therapy for myotonic dystrophy type 1. *Current Treatment Options in Neurology*, *25*(8), 261-279. https://doi.org/10.1007/s11940-023-00760-9 | No cognitive, Wrong publication type |
| Cognitive phenotype of childhood myotonic dystrophy type 1: A multicenter pooled analysis | 10.1002/mus.27836 | Sweere, D.J.J., Moelands, S.V.L., Klinkenberg, S., Leenen, L., Hendriksen, J.G.M. and Braakman, H.M.H.(2023). Cognitive phenotype of childhood myotonic dystrophy type 1: A multicenter pooled analysis. *Muscle and Nerve*, *68*(1), 57-64. https://doi.org/10.1002/mus.27836 | Wrong study design |
| Clearance of defective muscle stem cells by senolytics restores myogenesis in myotonic dystrophy type 1 | 10.1038/s41467-023-39663-3 | Conte, T.C., Duran-Bishop, G., Orfi, Z., Mokhtari, I., Deprez, A., Côté, I., Molina, T., Kim, T.-Y., Tellier, L., Roussel, M.-P., Maggiorani, D., Benabdallah, B., Leclerc, S., Feulner, L., Pellerito, O., Mathieu, J., Andelfinger, G., Gagnon, C., Beauséjour, C., McGraw, S., Duchesne, E. and Dumont, N.A. (2023). Clearance of defective muscle stem cells by senolytics restores myogenesis in myotonic dystrophy type 1. *Nature Communications*, *14*(1). https://doi.org/10.1038/s41467-023-39663-3 | No cognitive, Wrong study design |
| Natural antioxidants reduce oxidative stress and the toxic effects of RNA-CUG(exp) in an inducible glial myotonic dystrophy type 1 cell model | 10.3390/antiox14030260 | Morales, F., Vargas, D., Palma-Jiménez, M., Rodríguez, E.J., Azofeifa, G. and Hernández-Hernández, O. (2025). Natural antioxidants reduce oxidative stress and the toxic effects of RNA-CUG(exp) in an inducible glial myotonic dystrophy type 1 cell model. *Antioxidants*, *14*(3). https://doi.org/10.3390/antiox14030260 | No cognitive |
| A case of myotonic dystrophy type 1 with severe dilated cardiomyopathy: an unusual presenting manifestation of the most common muscular dystrophy in adults | 10.1007/s10072-024-07535-3 | Ghosh, R., León-Ruiz, M., Mondal, A.S., Dubey, S. and Benito-León, J. (2024). A case of myotonic dystrophy type 1 with severe dilated cardiomyopathy: an unusual presenting manifestation of the most common muscular dystrophy in adults. *Neurological Sciences*, *45*(9), 4629-4631. https://doi.org/10.1007/s10072-024-07535-3 | Wrong publication type |
| Fatigue in myotonic dystrophy type 1: a seven-year prospective study | Not found | Peric, S., Bjelica, B., Bozovic, I., Pesovic, J., Paunic, T., Banovic, M., Brkusanin, M., Aleksic, K., Basta, I., Pavicevic, D.S. and Stojanovic, V.R. (2019). Fatigue in myotonic dystrophy type 1: a seven-year prospective study. *Acta Myologica*, *38*(4), 239-244. | No cognitive |
| Myotonic dystrophy and primary care | 10.1016/j.semerg.2020.09.001 | Domínguez-González, C. (2020). Myotonic dystrophy and primary care. *Semergen*, *46*(7), 439-440. https://doi.org/10.1016/j.semerg.2020.09.001 | No cognitive |
| Myotonic dystrophy type 1 accompanied with normal pressure hydrocephalus: a case report and literature review | 10.1186/s12883-020-01636-0 | Wang, J., Liu, M., Shang, W., Chen, Z. and Peng, G. (2020). Myotonic dystrophy type 1 accompanied with normal pressure hydrocephalus: A case report and literature review. *BMC Neurology*, *20*(1). https://doi.org/10.1186/s12883-020-01636-0 | Wrong study design |
| Analysis of the functional capacity outcome measures for myotonic dystrophy | 10.1002/acn3.50845 | Jimenez-Moreno, A.C., Nikolenko, N., Kierkegaard, M., Blain, A.P., Newman, J., Massey, C., Moat, D., Sodhi, J., Atalaia, A., Gorman, G.S., Turner, C. and Lochmüller, H.(2019). Analysis of the functional capacity outcome measures for myotonic dystrophy. *Annals of Clinical and Translational Neurology*, *6*(8), 1487-1497. https://doi.org/10.1002/acn3.50845 | No cognitive |
| Myotonic dystrophy: an RNA toxic gain of function tauopathy? | 10.1007/978-981-32-9358-8_17 | Fernandez-Gomez, F., Tran, H., Dhaenens, C.-M., Caillet-Boudin, M.-L., Schraen-Maschke, S., Blum, D., Sablonnière, B., Buée-Scherrer, V., Buee, L. and Sergeant, N. (2019). Myotonic dystrophy: An RNA toxic gain of function tauopathy? *Advances in Experimental edicine and Biology*, *1184*, 207-216. https://doi.org/10.1007/978-981-32-9358-8_17 | No cognitive, Wrong publication type |
| Encoding of facial expressions in individuals with adult-onset myotonic dystrophy type 1 | 10.1080/13803395.2020.1826410 | Johnson, C., Langbehn, K.E., Long, J.D., Moser, D., Cross, S., Gutmann, L., Nopoulos, P.C. and van der Plas, E. (2020). Encoding of facial expressions in individuals with adult-onset myotonic dystrophy type 1. *Journal of Clinical and Experimental Neuropsychology*, *42*(9), 932-940. https://doi.org/10.1080/13803395.2020.1826410 | Wrong study design |
| Sleep disorders in four patients with myotonic dystrophy type 1 | 10.3389/fneur.2020.00012 | Urata, Y., Nakamura, M., Shiokawa, N., Yasuniwa, A., Takamori, N., Imamura, K., Hayashi, T., Ishizuka, T., Kasugai, M. and Sano, A. (2020). Sleep disorders in four patients with myotonic dystrophy type 1. *Frontiers in Neurology*, *11*. https://doi.org/10.3389/fneur.2020.00012 | No cognitive |
| MSH3 modifies somatic instability and disease severity in Huntington's and myotonic dystrophy type 1 | 10.1093/brain/awz115 | Flower, M., Lomeikaite, V., Ciosi, M., Cumming, S., Morales, F., Lo, K., Hensman Moss, D., Jones, L., Holmans, P., Monckton, D.G., Tabrizi, S.J., Kraus, P., Hoffman, R., Tobin, A., Borowsky, B., Keenan, S., Whitlock, K.B., Queller, S., Campbell, C., Wang, C., Langbehn, D., Axelson, E., Johnson, H., Acharya, T., Cash, D.M., Frost, C., Jones, R., Jurgens, C., Hart, E.P.T., Van Der Grond, J., Witjes- Ane, M.-N.N., Roos, R.A.C., Dumas, E.M., Van Den Bogaard, S.J.A., Stopford, C., Craufurd, D., Callaghan, J., Arran, N., Rosas, D.D., Lee, S., Monaco, W., O'Regan, A., Milchman, C., Frajman, E., Labuschagne, I., Stout, J., Campbell, M., Andrews, S.C., Bechtel, N., Reilmann, R., Bohlen, S., Kennard, C., Berna, C., Hicks, S., Durr, A., Pourchot, C., Bardinet, E., Nigaud, K., Valabrègue, R., Lehericy, S., Marelli, C., Jauffret, C., Justo, D., Leavitt, B., Decolongon, J., Sturrock, A., Coleman, A., Dar Santos, R., Patel, A., Gibbard, C., Whitehead, D., Wild, E., Owen, G., Crawford, H., Malone, I., Lahiri, N., Fox, N.C., Hobbs, N.Z., Scahill, R.I., Ordidge, R., Pepple, T., Read, J., Say, M.J., Landwehrmeyer, B., Daidj, F., Bassez, G., Lignier, B., Couppey, F., Delmas, S., Deux, J.-F., Hankiewicz, K., Dogan, C., Minier, L., Chevalier, P., Hamadouche, A., Catt, M., Van Hees, V., Catt, S., Schwalber, A., Dittrich, J., Kierkegaard, M., Wenninger, S., Schoser, B., Schüller, A., Stahl, K., Künzel, H., Wolff, M., Jellinek, A., Moreno, C.J., Gorman, G., Lochmüller, H., Trenell, M., Van Laar, S., Wood, L., Cassidy, S., Newman, J., Charman, S., Steffaneti, R., Taylor, L., Brownrigg, A., Day, S., Atalaia, A., Raaphorst, J., Okkersen, K., Van Engelen, B., Nikolaus, S., Cornelissen, Y., Van Nimwegen, M., Maas, D., Klerks, E., Bouman, S., Knoop, H., Heskamp, L., Heerschap, A., Rahmadi, R., Groot, P., Heskes, T., Kapusta, K., Glennon, J., Abghari, S., Aschrafi, A., Poelmans, G., Treweek, S., Hogarth, F., Littleford, R., Donnan, P., Hapca, A., Hannah, M., Mckenzie, E., Rauchhaus, P., Adam, B., Faber, C. and Merkies, I. (2019). MSH3 modifies somatic instability and disease severity in huntington's and myotonic dystrophy type 1. *Brain*, *142*(7), 1876-1886. https://doi.org/10.1093/brain/awz115 | No DM1,No cognitive |
| Three-dimensional assessment of pharyngeal airway in individuals with myotonic dystrophy type 1 | 10.3906/sag-2105-106 | Evlice, B., Koç, F., Duyan, H. and Çabuk, D.S.(2021). Three-dimensional assessment of pharyngeal airway in individuals with myotonic dystrophy type 1. *Turkish Journal of Medical Sciences*, *51*(6), 3022-3029. https://doi.org/10.3906/sag-2105-106 | No cognitive |
| Predicting daytime sleepiness and fatigue: a 9-year prospective study in myotonic dystrophy type 1 | 10.1007/s00415-019-09592-7 | Laberge, L., Gallais, B., Auclair, J., Dauvilliers, Y., Mathieu, J. and Gagnon, C.(2020). Predicting daytime sleepiness and fatigue: A 9-year prospective study in myotonic dystrophy type 1. *Journal of Neurology*, *267*(2), 461-468. https://doi.org/10.1007/s00415-019-09592-7 | No cognitive |
| Altered REM sleep architecture in patients with myotonic dystrophy type 1: Is related to sleep apnea? | 10.1016/j.sleep.2020.12.036 | Seshagiri, D.V., Huddar, A., Nashi, S., Ray, S., Ramaswamy, P., Oommen, A.T., Chawla, T., Yadav, S., Annapureddy, J., Jankar, R., Polavarapu, K., Vengalil, S., Preethish-Kumar, V., Warrier, M., Thomas, P.T., Shingavi, L., Arunachal, G., Yadav, R., and Nalini, A.(2021). Altered REM sleep architecture in patients with myotonic dystrophy type 1: Is related to sleep apnea? *Sleep Medicine*, *79*, 48-54. https://doi.org/10.1016/j.sleep.2020.12.036 | No cognitive, Wrong study design |
| Associations between variant repeat interruptions and clinical outcomes in myotonic dystrophy type 1 | 10.1212/NXG.0000000000000572 | Wenninger, S., Cumming, S.A., Gutschmidt, K., Okkersen, K., Jimenez-Moreno, A.C., Daidj, F., Lochmüller, H., Hogarth, F., Knoop, H., Bassez, G., Monckton, D.G., Van Engelen, B.G.M. and Schoser, B.(2021). Associations between variant repeat interruptions and clinical outcomes in myotonic dystrophy type 1. *Neurology: Genetics*, *7*(2), e572. https://doi.org/10.1212/NXG.0000000000000572 | No cognitive, Wrong study design |
| Meet and eat, an interdisciplinary group intervention for patients with myotonic dystrophy about healthy nutrition, meal preparation, and consumption: a feasibility study | 10.1080/09638288.2018.1528305 | van Hees, S.G.M., Knuijt, S., Dicke, H., Groothuis, J.T., Raaphorst, J., Satink, T. and Cup, E.H.C.(2020). Meet and eat, an interdisciplinary group intervention for patients with myotonic dystrophy about healthy nutrition, meal preparation, and consumption: a feasibility study. *Disability and Rehabilitation*, *42*(11), 1561-1568. https://doi.org/10.1080/09638288.2018.1528305 | No cognitive |
| Reply to the letter entitled “predictors of respiratory impairment in patients with myotonic dystrophy type 1” | 10.1016/j.jns.2019.05.025 | Rossi, S., Della Marca, G., Ricci, M., Perna, A., Nicoletti, T.F., Brunetti, V., Meleo, E., Calvello, M., Petrucci, A., Antonini, G., Bucci, E., Licchelli, L., Sancricca, C., Massa, R., Rastelli, E., Botta, A., Di Muzio, A., Romano, S., Garibaldi, M. and Silvestri, G. (2019). Reply to the letter entitled “predictors of respiratory impairment in patients with myotonic dystrophy type 1”. *Journal of the Neurological Sciences*, *403*, 166-167. https://doi.org/10.1016/j.jns.2019.05.025 | No cognitive, Wrong publication type |
| A large multicenter study of pediatric myotonic dystrophy type 1 for evidence-based management | 10.1212/WNL.0000000000006948 | Lagrue, E., Dogan, C., De Antonio, M., Audic, F., Bach, N., Barnerias, C., Bellance, R., Cances, C., Chabrol, B., Cuisset, J.-M., Desguerre, I., Durigneux, J., Espil, C., Fradin, M., Héron, D., Isapof, A., Jacquin-Piques, A., Journel, H., Laroche-Raynaud, C., Laugel, V., Magot, A., Manel, V., Mayer, M., Péréon, Y., Perrier-Boeswillald, J., Peudenier, S., Quijano-Roy, S., Ragot-Mandry, S., Richelme, C., Rivier, F., Sabouraud, P., Sarret, C., Testard, H., Vanhulle, C., Walther-Louvier, U., Gherardi, R., Hamroun, D. and Bassez, G.(2019). A large multicenter study of pediatric myotonic dystrophy type 1 for evidence-based management. *Neurology*, *92*(8), E852-E865. https://doi.org/10.1212/WNL.0000000000006948 | Wrong publication type |
| Change over time in ability to perform activities of daily living in myotonic dystrophy type 1 | 10.1007/s00415-020-09970-6 | Landfeldt, E., Nikolenko, N., Jimenez-Moreno, C., Cumming, S., Monckton, D.G., Faber, C.G., Merkies, I.S.J., Gorman, G., Turner, C. and Lochmüller, H. (2020). Change over time in ability to perform activities of daily living in myotonic dystrophy type 1. *Journal of neurology*, *267*(11), 3235-3242. https://doi.org/10.1007/s00415-020-09970-6 | No cognitive |
| Electrocardiographic predictors of infrahissian conduction disturbances in myotonic dystrophy type 1 | 10.1093/europace/euaa256 | Joosten, I.B.T., Van Lohuizen, R., Den Uijl, D.W., Evertz, R., De Greef, B.T.A., Van Engelen, B.G.M., Faber, C.G. and Vernooy, K.(2021). Electrocardiographic predictors of infrahissian conduction disturbances in myotonic dystrophy type 1. *Europace*, *23*(2), 298-304. https://doi.org/10.1093/europace/euaa256 | No cognitive |
| Acti-DM1: Monitoring the activity level of people with myotonic dystrophy type 1 through activity and exercise recognition | 10.1109/ACCESS.2021.3068877 | Chapron, K., Lapointe, P., Lessard, I., Darsmstadt-Bélanger, H., Bouchard, K., Gagnon, C., Lavoie, M., Duchesne, E. and Gaboury, S.(2021). Acti-DM1: monitoring the activity level of people with myotonic dystrophy type 1 through activity and exercise recognition. *IEEE Access*, *9*, 49960-49973. https://doi.org/10.1109/ACCESS.2021.3068877 | No cognitive, Wrong study design |
| Validation of motor outcome measures in myotonic dystrophy type 2 | 10.3389/fneur.2020.00306 | Montagnese, F., Rastelli, E., Khizanishvili, N., Massa, R., Stahl, K. and Schoser, B. (2020). Validation of motor outcome measures in myotonic dystrophy type 2. *Frontiers in Neurology*, *11*. https://doi.org/10.3389/fneur.2020.00306 | No DM1 |
| Multisystemic impairments in 93 chinese patients with myotonic dystrophy type 1 | 10.3389/fneur.2020.00277 | Li, M., Wang, Z., Cui, F., Yang, F., Wang, H. and Huang, X. (2020). Multisystemic impairments in 93 Chinese patients with myotonic dystrophy type 1. *Frontiers in neurology*, *11*. https://doi.org/10.3389/fneur.2020.00277 | No cognitive |
| High incidence of falls in patients with myotonic dystrophy type 1 and 2: A prospective study | 10.1016/j.nmd.2019.08.012 | Berends, J., Tieleman, A.A., Horlings, C.G.C., Smulders, F.H.P., Voermans, N.C., van Engelen, B.G.M. and Raaphorst, J. (2019). High incidence of falls in patients with myotonic dystrophy type 1 and 2: A prospective study. *Neuromuscular Disorders*, *29*(10), 758-765. https://doi.org/10.1016/j.nmd.2019.08.012 | No cognitive |
| Multiple case study of changes in participation of adults with myotonic dystrophy type 1: Importance of redesigning accomplishment and resilience | 10.3233/JND-210780 | Raymond, K., Gagnon, C. and Levasseur, M. (2022). Multiple case study of changes in participation of adults with myotonic dystrophy type 1: importance of redesigning accomplishment and resilience. *Journal of Neuromuscular Diseases*, *9*(6), 731-755. https://doi.org/10.3233/JND-210780 | Wrong publication type |
| Myotonic dystrophy type 1 with anterior temporal white matter changes mimicking cerebral autosomal dominant arteriopathy with subcortical infarcts and leukoencephalopathy | Not found | Kim, H.S., Kim, Y.E., Lee, J. and Ma, H.-I.(2020). Myotonic dystrophy type 1 with anterior temporal white matter changes mimicking cerebral autosomal dominant arteriopathy with subcortical infarcts and leukoencephalopathy. *Neurology Asia*, *25*(3), 395-397. | Wrong publication type |
| Health-related quality of life in patients with adult-onset myotonic dystrophy type 1: A systematic review | 10.1007/s40271-019-00357-y | Landfeldt, E., Edström, J., Jimenez-Moreno, C., van Engelen, B.G.M., Kirschner, J. and Lochmüller, H. (2019). Health-related quality of life in patients with adult-onset myotonic dystrophy type 1: A systematic review. *Patient*, *12*(4), 365-373. https://doi.org/10.1007/s40271-019-00357-y | Wrong publication type |
| Perceived fatigue in myotonic dystrophy type 1: A case-control study | 10.1186/s12883-019-1280-z | Winblad, S. and Lindberg, C.(2019). Perceived fatigue in myotonic dystrophy type 1: A case-control study. *BMC Neurology*, *19*(1). https://doi.org/10.1186/s12883-019-1280-z | Wrong publication type |
| Brain pathogenesis and potential therapeutic strategies in myotonic dystrophy type 1 | 10.3389/fnagi.2021.755392 | Liu, J., Guo, Z.-N., Yan, X.-L., Yang, Y. and Huang, S. (2021). Brain pathogenesis and potential therapeutic strategies in myotonic dystrophy type 1. *Frontiers in Aging Neuroscience*, *13*. https://doi.org/10.3389/fnagi.2021.755392 | Wrong study design |
| Neurocognitive features of motor premanifest individuals with myotonic dystrophy type 1 | 10.1212/NXG.0000000000000577 | Van Der Plas, E., Koscik, T.R., Magnotta, V., Cumming, S.A., Monckton, D., Gutmann, L. and Nopoulos, P. (2021). Neurocognitive features of motor premanifest individuals with myotonic dystrophy type 1. *Neurology: genetics*, *7*(2). https://doi.org/10.1212/NXG.0000000000000577 | Wrong study design |
| Two adult siblings with myotonic dystrophy type 1 with different phenotypes presenting with chronic respiratory insufficiency and sleep apnea syndrome | 10.3389/fneur.2019.00681 | Kohno, G., Ogawa, K., Kushimoto, M., Tanaka, S., Ikeda, J., Nishizawa, T., Mitsuke, K., Nakayama, T., Suzuki, Y., Ishihara, H. and Fujishiro, M. (2019). Two adult siblings with myotonic dystrophy type 1 with different phenotypes presenting with chronic respiratory insufficiency and sleep apnea syndrome. *Frontiers in Neurology*, *10*. https://doi.org/10.3389/fneur.2019.00681 | No cognitive, Wrong study design, Wrong publication type |
| Lower extremity muscle pathology in myotonic dystrophy type 1 assessed by quantitative MRI | 10.1212/WNL.0000000000007648 | Heskamp, L., Van Nimwegen, M., Ploegmakers, M.J., Bassez, G., Deux, J.-F., Cumming, S.A., Monckton, D.G., Van Engelen, B.G.M. and Heerschap, A. (2019). Lower extremity muscle pathology in myotonic dystrophy type 1 assessed by quantitative mri. *Neurology*, *92*(24), E2803-E2814. https://doi.org/10.1212/WNL.0000000000007648 | No cognitive |
| Progressive decline in daily and social activities: A 9-year longitudinal study of participation in myotonic dystrophy type 1 | 10.1016/j.apmr.2019.01.022 | Raymond, K., Levasseur, M., Mathieu, J. and Gagnon, C. (2019). Progressive decline in daily and social activities: a 9-year longitudinal study of participation in myotonic dystrophy type 1. *Archives of Physical Medicine and Rehabilitation*, *100*(9), 1629-1639. https://doi.org/10.1016/j.apmr.2019.01.022 | No cognitive |
| Protective effects of mirtazapine in mice lacking the MBNL2 gene in forebrain glutamatergic neurons: Relevance for myotonic dystrophy 1 | 10.1016/j.neuropharm.2020.108030 | Ramon-Duaso, C., Rodríguez-Morató, J., Selma-Soriano, E., Fernández-Avilés, C., Artero, R., de la Torre, R., Pozo, Ó.J. and Robledo, P. (2020). Protective effects of mirtazapine in mice lacking the mbnl2 gene in forebrain glutamatergic neurons: relevance for myotonic dystrophy 1. *Neuropharmacology*, *170*. https://doi.org/10.1016/j.neuropharm.2020.108030 | No cognitive, Wrong study design |
| Diffusion tensor imaging and voxel-based morphometry reveal corticospinal tract involvement in the motor dysfunction of adult-onset myotonic dystrophy type 1 | 10.1038/s41598-018-34048-9 | Park, J.-S., Song, H., Jang, K.E., Cha, H., Lee, S.-H., Hwang, S.-K., Park, D., Lee, H.J., Kim, J.-Y. and Chang, Y.(2018). Diffusion tensor imaging and voxel-based morphometry reveal corticospinal tract involvement in the motor dysfunction of adult-onset myotonic dystrophy type 1. *Scientific reports*, *8*(1). https://doi.org/10.1038/s41598-018-34048-9 | No cognitive, Wrong study design |
| Molecular and clinical implications of variant repeats in myotonic dystrophy type 1 | 10.3390/ijms23010354 | Peric, S., Pesovic, J., Savic-Pavicevic, D., Stojanovic, V.R., and Meola, G.(2022). Molecular and clinical implications of variant repeats in myotonic dystrophy type 1. *International Journal of Molecular Sciences*, *23*(1), . https://doi.org/10.3390/ijms23010354 | No cognitive, Wrong publication type |
| Regional brain atrophy in gray and white matter is associated with cognitive impairment in myotonic dystrophy type 1 | 10.1016/j.nicl.2019.102078 | Labayru, G., Diez, I., Sepulcre, J., Fernández, E., Zulaica, M., Cortés, J.M., López de Munain, A. and Sistiaga, A.(2019). Regional brain atrophy in gray and white matter is associated with cognitive impairment in myotonic dystrophy type 1. *Neuroimage: Clinical*, *24*. https://doi.org/10.1016/j.nicl.2019.102078 | Wrong study design |
| Impact of a 12-week strength training program on fatigue, daytime sleepiness, and apathy in men with myotonic dystrophy type 1 | 10.3233/JND-221503 | Gallais, B., Roussel, M.-P., Laberge, L., Hébert, L.J., and Duchesne, E.(2022). Impact of a 12-week strength training program on fatigue, daytime sleepiness, and apathy in men with myotonic dystrophy type 1. *Journal of Neuromuscular Diseases*, *9*(5), 629-639. https://doi.org/10.3233/JND-221503 | No cognitive |
| Responsiveness of outcome measures in myotonic dystrophy type 1 | 10.1002/acn3.51129 | Knak, K.L., Sheikh, A.M., Witting, N. and Vissing, J. (2020). Responsiveness of outcome measures in myotonic dystrophy type 1. *Annals of Clinical and Translational Neurology*, *7*(8), 1382-1391. https://doi.org/10.1002/acn3.51129 | No cognitive |
| Myotonic dystrophy | 10.1007/978-3-031-11136-5_36 | Dubon, M.E., Martin, E.N. and Paulson, A.(2022). Myotonic dystrophy. *Orthopaedics for the Newborn and Young Child: A Practical Clinical Guide*. 371-387. https://doi.org/10.1007/978-3-031-11136-5_36 | No cognitive, Wrong publication type |
| Epigenetics of myotonic dystrophies: a minireview | 10.3390/ijms222212594 | Visconti, V.V., Centofanti, F., Fittipaldi, S., Macrì, E., Novelli, G. and Botta, A.(2021). Epigenetics of myotonic dystrophies: A minireview. *International Journal of Molecular Sciences*, *22*(22), . https://doi.org/10.3390/ijms222212594 | No cognitive, Wrong publication type |
| Cerebral involvement and related aspects in myotonic dystrophy type 2 | 10.1016/j.nmd.2021.06.002 | Peric, S., Rakocevic-Stojanovic, V. and Meola, G.(2021). Cerebral involvement and related aspects in myotonic dystrophy type 2. *Neuromuscular Disorders*, *31*(8), 681-694. https://doi.org/10.1016/j.nmd.2021.06.002 | No DM1, Wrong publication type |
| Predictors of participation restriction over a 9-year period in adults with myotonic dystrophy type 1 | 10.1080/09638288.2020.1837264 | Raymond, K., Levasseur, M., Gallais, B., Richer, L., Laberge, L., Petitclerc, É., Mathieu, J. and Gagnon, C. (2022). Predictors of participation restriction over a 9-year period in adults with myotonic dystrophy type 1. *Disability and Rehabilitation*, *44*(12), 2615-2631. https://doi.org/10.1080/09638288.2020.1837264 | No cognitive |
| Unravelling the impact of frontal lobe impairment for social dysfunction in myotonic dystrophy type 1 | 10.1093/braincomms/fcac111 | Morin, A., Funkiewiez, A., Routier, A., Le Bouc, R., Borderies, N., Galanaud, D., Levy, R., Pessiglione, M., Dubois, B., Eymard, B., Michon, C.-C., Angeard, N., Behin, A., Laforet, P., Stojkovic, T. and Azuar, C.(2022). Unravelling the impact of frontal lobe impairment for social dysfunction in myotonic dystrophy type 1. *Brain communications*, *4*(3). https://doi.org/10.1093/braincomms/fcac111 | Wrong study design |
| Myotonic dystrophy type 1 (DM1) clinical subtypes and CTCF site methylation status flanking the CTG expansion are mutant allele length-dependent | 10.1093/hmg/ddab243 | Morales, F., Corrales, E., Zhang, B., Vásquez, M., Santamaría-Ulloa, C., Quesada, H., Sirito, M., Estecio, M.R., Monckton, D.G. and Krahe, R.(2022). Myotonic dystrophy type 1 (DM1) clinical subtypes and CTCF site methylation status flanking the CTG expansion are mutant allele length-dependent. *Human Molecular Genetics*, *31*(2), 262-274. https://doi.org/10.1093/hmg/ddab243 | No cognitive, Wrong study design |
| Central nervous system involvement as outcome measure for clinical trials efficacy in myotonic dystrophy type 1 | 10.3389/fneur.2020.00624 | Simoncini, C., Spadoni, G., Lai, E., Santoni, L., Angelini, C., Ricci, G. and Siciliano, G.(2020). Central nervous system involvement as outcome measure for clinical trials efficacy in myotonic dystrophy type 1. *Frontiers in Neurology*, 11. https://doi.org/10.3389/fneur.2020.00624 | Wrong publication type |
| Physical activity in myotonic dystrophy type 1 | 10.1007/s00415-020-09758-8 | Knak, K.L., Sheikh, A.M., Witting, N. and Vissing, J. (2020). Physical activity in myotonic dystrophy type 1. *Journal of Neurology*, *267*(6), 1679-1686. https://doi.org/10.1007/s00415-020-09758-8 | No cognitive |
| Differences in the pattern of cognitive impairments between juvenile and adult onset myotonic dystrophy type 1 | 10.1016/j.jocn.2019.07.029 | Woo, J., Lee, H.-W. and Park, J.-S. (2019). Differences in the pattern of cognitive impairments between juvenile and adult onset myotonic dystrophy type 1. *Journal of Clinical Neuroscience*, *68*, 92-96. https://doi.org/10.1016/j.jocn.2019.07.029 | Wrong study design |
| Characteristics of myotonic dystrophy patients in the national registry of japan | 10.1016/j.jns.2021.120080 | Sugimoto, M., Kuru, S., Takada, H., Horie, R., Yamauchi, K., Kubota, T., Matsumura, T., Nakamura, H., Kimura, E. and Takahashi, M.P.(2022). Characteristics of myotonic dystrophy patients in the national registry of Japan. *Journal of the Neurological Sciences*, *432*. https://doi.org/10.1016/j.jns.2021.120080 | No cognitive, Wrong study design |
| Clinical and genetic characteristics of childhood-onset myotonic dystrophy | 10.1002/mus.26716 | Stokes, M., Varughese, N., Iannaccone, S., and Castro, D.(2019). Clinical and genetic characteristics of childhood-onset myotonic dystrophy. *Muscle and Nerve*, *60*(6), 732-738. https://doi.org/10.1002/mus.26716 | No cognitive, Wrong publication type |
| Characterization of ran translation and antisense transcription in primary cell cultures of patients with myotonic dystrophy type 1 | 10.3390/jcm10235520 | Koehorst, E., Núñez‐manchón, J., Ballester‐López, A., Almendrote, M., Lucente, G., Arbex, A., Chojnacki, J., Vázquez‐manrique, R.P., Gómez‐escribano, A.P., Pintos‐morell, G., Coll‐cantí, J., Ramos‐fransi, A., Martínez‐piñeiro, A., Suelves, M. and Nogales‐gadea, G. (2021). Characterization of ran translation and antisense transcription in primary cell cultures of patients with myotonic dystrophy type 1. *Journal of Clinical Medicine*, *10*(23). https://doi.org/10.3390/jcm10235520 | No cognitive, Wrong study design |
| Recognition of emotions conveyed by facial expression and body postures in myotonic dystrophy (dm) | 10.1016/j.cortex.2020.02.005 | Lenzoni, S., Bozzoni, V., Burgio, F., de Gelder, B., Wennberg, A., Botta, A., Pegoraro, E. and Semenza, C. (2020). Recognition of emotions conveyed by facial expression and body postures in myotonic dystrophy (dm). *Cortex*, *127*, 58-66. https://doi.org/10.1016/j.cortex.2020.02.005 | Wrong study design |
| Transcriptome alterations in myotonic dystrophy frontal cortex | 10.1016/j.celrep.2020.108634 | Otero, B.A., Poukalov, K., Hildebrandt, R.P., Thornton, C.A., Jinnai, K., Fujimura, H., Kimura, T., Hagerman, K.A., Sampson, J.B., Day, J.W. and Wang, E.T. (2021). Transcriptome alterations in myotonic dystrophy frontal cortex. *Cell reports*, *34*(3). https://doi.org/10.1016/j.celrep.2020.108634 | No cognitive |
| Current treatment options for patients with myotonic dystrophy type 2 | 10.1007/s11940-021-00686-0 | Montagnese, F.(2021). Current treatment options for patients with myotonic dystrophy type 2. *Current Treatment Options in Neurology*, *23*(9). https://doi.org/10.1007/s11940-021-00686-0 | No cognitive, Wrong publication type |
| Transcriptional signatures of synaptic vesicle genes define myotonic dystrophy type i neurodegeneration | 10.1111/nan.12725 | Jimenez-Marin, A., Diez, I., Labayru, G., Sistiaga, A., Caballero, M.C., Andres-Benito, P., Sepulcre, J., Ferrer, I., Lopez de Munain, A. and Cortes, J.M. (2021). Transcriptional signatures of synaptic vesicle genes define myotonic dystrophy type i neurodegeneration. *Neuropathology and Applied Neurobiology*, *47*(7), 1092-1108. https://doi.org/10.1111/nan.12725 | No cognitive, Wrong study design |
| Ventral tegmental area dysfunction affects decision-making in patients with myotonic dystrophy type-1 | 10.1016/j.cortex.2020.03.022 | Serra, L., Scocchia, M., Meola, G., D'Amelio, M., Bruschini, M., Silvestri, G., Petrucci, A., Di Domenico, C., Caltagirone, C., Koch, G., Cercignani, M., Petrosini, L. and Bozzali, M. (2020). Ventral tegmental area dysfunction affects decision-making in patients with myotonic dystrophy type-1. *Cortex*, *128*, 192-202. https://doi.org/10.1016/j.cortex.2020.03.022 | Wrong study design |
| Lesion distribution and substrate of white matter damage in myotonic dystrophy type 1: Comparison with multiple sclerosis | 10.1016/j.nicl.2021.102562 | Leddy, S., Serra, L., Esposito, D., Vizzotto, C., Giulietti, G., Silvestri, G., Petrucci, A., Meola, G., Lopiano, L., Cercignani, M., and Bozzali, M. (2021). Lesion distribution and substrate of white matter damage in myotonic dystrophy type 1: Comparison with multiple sclerosis. *Neuroimage: Clinical*, *29*. https://doi.org/10.1016/j.nicl.2021.102562 | No cognitive |
| Swallow-related quality of life and oropharyngeal dysphagia in myotonic dystrophy | 10.1007/s00405-020-05964-2 | Pilz, W., Passos, V.L., Verdonschot, R.J., Meijers, J., Roodenburg, N., Halmans, Y., Faber, C.G., Kremer, B. and Baijens, L.W.J. (2020). Swallow-related quality of life and oropharyngeal dysphagia in myotonic dystrophy. *European Archives of Oto-rhino-laryngology*, *277*(8), 2357-2362. https://doi.org/10.1007/s00405-020-05964-2 | No cognitive |
| Altered local brain amplitude of fluctuations in patients with myotonic dystrophy type 1 | 10.3389/fnagi.2021.790632 | Huang, P., Luan, X.-H., Xie, Z., Li, M.-T., Chen, S.-D., Liu, J., Jia, X.-Z., Cao, L. and Zhou, H.-Y. (2021). Altered local brain amplitude of fluctuations in patients with myotonic dystrophy type 1. *Frontiers in Aging Neuroscience*, *13*. https://doi.org/10.3389/fnagi.2021.790632 | No cognitive |
| Subjective-objective sleepiness discrepancy in adult-onset myotonic dystrophy type 1 | 10.5664/jcsm.9722 | Gorantla, S., Blume, G. and Grigg-Damberger, M. (2021). Subjective-objective sleepiness discrepancy in adult-onset myotonic dystrophy type 1. *Journal of Clinical Sleep Medicine*, *17*(12), 2351-2352. https://doi.org/10.5664/jcsm.9722 | No cognitive |
| Accomplishment of instrumental activities of daily living and its relationship with cognitive functions in adults with myotonic dystrophy type 1 childhood phenotype: An exploratory study | 10.1186/s40359-021-00562-1 | Tremblay, M., Muslemani, S., Côté, I., Gagnon, C., Fortin, J. and Gallais, B.(2021). Accomplishment of instrumental activities of daily living and its relationship with cognitive functions in adults with myotonic dystrophy type 1 childhood phenotype: an exploratory study. *BMC Psychology*, *9*(1). https://doi.org/10.1186/s40359-021-00562-1 | Wrong study design |
| Dyslexia and cognitive impairment in adult patients with myotonic dystrophy type 1: A clinical prospective analysis | 10.1007/s00415-020-10161-6 | Gutschmidt, K., Wenninger, S., Montagnese, F., and Schoser, B. (2021). Dyslexia and cognitive impairment in adult patients with myotonic dystrophy type 1: A clinical prospective analysis. *Journal of Neurology*, *268*(2), 484-492. https://doi.org/10.1007/s00415-020-10161-6 | Wrong publication type |
| How to capture activities of daily living in myotonic dystrophy type 2? | 10.1016/j.nmd.2020.07.011 | Montagnese, F., Rastelli, E., Stahl, K., Massa, R. and Schoser, B. (2020). How to capture activities of daily living in myotonic dystrophy type 2? *Neuromuscular Disorders*, *30*(10), 796-806. https://doi.org/10.1016/j.nmd.2020.07.011 | No DM1 |
| Cerebral ventriculomegaly in myotonic dystrophy type 1: Normal pressure hydrocephalus-like appearances on magnetic resonance imaging | 10.1186/s12868-021-00667-8 | Iida, S., Seino, H., Nagahata, F., Tatsuo, S., Maruyama, S., Kon, S., Takada, H., Matsuzaka, M., Sugimoto, K. and Kakeda, S. (2021). Cerebral ventriculomegaly in myotonic dystrophy type 1: Normal pressure hydrocephalus-like appearances on magnetic resonance imaging. *BMC Neuroscience*, *22*(1). https://doi.org/10.1186/s12868-021-00667-8 | No cognitive |
| Strength-training effectively alleviates skeletal muscle impairments in myotonic dystrophy type 1 | 10.1016/j.nmd.2020.02.015 | Roussel, M.-P., Hébert, L.J. and Duchesne, E. (2020). Strength-training effectively alleviates skeletal muscle impairments in myotonic dystrophy type 1. *Neuromuscular Disorders*, *30*(4), 283-293. https://doi.org/10.1016/j.nmd.2020.02.015 | No cognitive |
| Myotonic muscular dystrophies | 10.1212/CON.0000000000000793 | Johnson, N.E.(2019). Myotonic muscular dystrophies. *Continuum Lifelong Learning in Neurology*, *25*(6), 1682-1695. https://doi.org/10.1212/CON.0000000000000793 | Wrong publication type |
| Deciphering the mechanisms underlying brain alterations and cognitive impairment in congenital myotonic dystrophy | 10.1016/j.nbd.2021.105532 | De Serres-Bérard, T., Pierre, M., Chahine, M. and Puymirat, J. (2021). Deciphering the mechanisms underlying brain alterations and cognitive impairment in congenital myotonic dystrophy. *Neurobiology of Disease*, *160*. https://doi.org/10.1016/j.nbd.2021.105532 | Wrong publication type |
| Diagnosis of myotonic dystrophy based on resting state fMRI using convolutional neural networks | 10.1109/EMBC44109.2020.9176455 | Kamali, T., Hagerman, K.A., Day, J.W., Sampson, J., Lim, K.O., Mueller, B.A. and Wozniak, J. (2020). Diagnosis of myotonic dystrophy based on resting state fmri using convolutional neural networks. *Proceedings of the Annual International Conference of the IEEE Engineering in Medicine and Biology Society*, *2020*, 1714-1717. https://doi.org/10.1109/EMBC44109.2020.9176455 | No cognitive, Wrong study design |
| Brain structural features of myotonic dystrophy type 1 and their relationship with CTG repeats | 10.3233/JND-190397 | Van Der Plas, E., Hamilton, M.J., Miller, J.N., Koscik, T.R., Long, J.D., Cumming, S., Povilaikaite, J., Farrugia, M.E., McLean, J., Jampana, R., Magnotta, V.A., Gutmann, L., Monckton, D.G. and Nopoulos, P.C.(2019). Brain structural features of myotonic dystrophy type 1 and their relationship with CTG repeats. *Journal of Neuromuscular Diseases*, *6*(3), 321-332. https://doi.org/10.3233/JND-190397 | Wrong publication type |
| Clinical guide for the diagnosis and follow-up of myotonic dystrophy type 1, MD1 or steinert's disease | 10.1016/j.medcli.2018.10.028 | Gutiérrez Gutiérrez, G., Díaz-Manera, J., Almendrote, M., Azriel, S., Eulalio Bárcena, J., Cabezudo García, P., Camacho Salas, A., Casanova Rodríguez, C., Cobo, A.M., Díaz Guardiola, P., Fernández-Torrón, R., Gallano Petit, M.P., García Pavía, P., Gómez Gallego, M., Gutiérrez Martínez, A.J., Jericó, I., Kapetanovic García, S., López de Munaín Arregui, A., Martorell, L., Morís de la Tassa, G., Moreno Zabaleta, R., Muñoz-Blanco, J.L., Olivar Roldán, J., Pascual Pascual, S.I., Peinado Peinado, R., Pérez, H., Poza Aldea, J.J., Rabasa, M., Ramos, A., Rosado Bartolomé, A., Rubio Pérez, M.Á., Urtizberea, J.A., Zapata-Wainberg, G. and Gutiérrez-Rivas, E. (2019). Clinical guide for the diagnosis and follow-up of myotonic dystrophy type 1, md1 or steinert's disease. *Medicina Clinica*, *153*(2), 82.e1-82.e17. https://doi.org/10.1016/j.medcli.2018.10.028 | Wrong publication type |
| Utility and results from a patient-reported online survey in myotonic dystrophies types 1 and 2 | 10.1159/000511237 | Wenninger, S., Stahl, K., Montagnese, F. and Schoser, B. (2020). Utility and results from a patient-reported online survey in myotonic dystrophies types 1 and 2. *European Neurology*, *83*(5), 523-533. https://doi.org/10.1159/000511237 | No cognitive, Wrong study design |
| Look at the cognitive deficits in patients with myotonic dystrophy type 1: An exploratory research on the effects of virtual reality | 10.1097/MRR.0000000000000384 | Maresca, G., Portaro, S., Naro, A., Scarcella, I., Bramanti, P., Militi, D., Accorinti, M., de Luca, R. and Calabrò, R.S. (2020). Look at the cognitive deficits in patients with myotonic dystrophy type 1: An exploratory research on the effects of virtual reality. *International Journal of Rehabilitation Research*, *43*(1), 90-94. https://doi.org/10.1097/MRR.0000000000000384 | Wrong study design |
| 248th ENMC international workshop: Myotonic dystrophies: molecular approaches for clinical purposes, framing a european molecular research network, hoofddorp, the netherlands, 11–13 october 2019 | 10.1016/j.nmd.2020.03.011 | Wansink, D.G., Gourdon, G., van Engelen, B.G.M. and Schoser, B. (2020). 248th ENMC international workshop: Myotonic dystrophies: Molecular approaches for clinical purposes, framing a European molecular research network, hoofddorp, the Netherlands, 11–13 october 2019. *Neuromuscular Disorders*, *30*(6), 521-531. https://doi.org/10.1016/j.nmd.2020.03.011 | Wrong publication type |
| Prevalence and predictor factors of respiratory impairment in a large cohort of patients with myotonic dystrophy type 1 (DM1): A retrospective, cross sectional study | 10.1016/j.jns.2019.02.012 | Rossi, S., Della Marca, G., Ricci, M., Perna, A., Nicoletti, T.F., Brunetti, V., Meleo, E., Calvello, M., Petrucci, A., Antonini, G., Bucci, E., Licchelli, L., Sancricca, C., Massa, R., Rastelli, E., Botta, A., Di Muzio, A., Romano, S., Garibaldi, M. and Silvestri, G. (2019). Prevalence and predictor factors of respiratory impairment in a large cohort of patients with myotonic dystrophy type 1 (DM1): a retrospective, cross sectional study. *Journal of the Neurological Sciences*, 399, 118-124. https://doi.org/10.1016/j.jns.2019.02.012 | No cognitive |
| Cognitive function and its relationship with brain structure in myotonic dystrophy type 1 | 10.1002/jnr.24595 | Langbehn, K.E., van der Plas, E., Moser, D.J., Long, J.D., Gutmann, L. and Nopoulos, P.C. (2021). Cognitive function and its relationship with brain structure in myotonic dystrophy type 1. *Journal of Neuroscience Research*, *99*(1), 190-199. https://doi.org/10.1002/jnr.24595 | Wrong study design |
| Characterization of eeg-based functional brain networks in myotonic dystrophy type 1 | 10.1016/j.clinph.2020.05.014 | Biere, J., Okkersen, K., van Alfen, N., Kessels, R.P.C., Gouw, A.A., van Dorst, M., van Engelen, B., Stam, C.J. and Raaphorst, J. (2020). Characterization of eeg-based functional brain networks in myotonic dystrophy type 1. *Clinical Neurophysiology*, *131*(8), 1886-1895. https://doi.org/10.1016/j.clinph.2020.05.014 | Wrong study design |
| Intra-rater reliability and concurrent validity of quantified muscle testing for maximal knee extensors strength in men with myotonic dystrophy type 1 | 10.3233/JND-190388 | Roussel, M.-P., Hébert, L.J. and Duchesne, E. (2019). Intra-rater reliability and concurrent validity of quantified muscle testing for maximal knee extensors strength in men with myotonic dystrophy type 1. *Journal of Neuromuscular Diseases*, *6*(2), 233-240. https://doi.org/10.3233/JND-190388 | No cognitive |
| DNA methylation at the DMPK gene locus is associated with cognitive functions in myotonic dystrophy type 1 | 10.2217/epi-2020-0328 | Breton, E., Légaré, C., Overend, G., Guay, S.-P., Monckton, D., Mathieu, J., Gagnon, C., Richer, L., Gallais, B. and Bouchard, L.(2020). DNA methylation at the DMPK gene locus is associated with cognitive functions in myotonic dystrophy type 1. *Epigenomics*, *12*(23), 2051-2064. https://doi.org/10.2217/epi-2020-0328 | Wrong study design |
| Daily activity performance in congenital and childhood forms of myotonic dystrophy type 1: A population-based study | 10.1111/dmcn.14395 | Eriksson, B.-M., Ekström, A.-B. and Peny-Dahlstrand, M. (2020). Daily activity performance in congenital and childhood forms of myotonic dystrophy type 1: a population-based study. *Developmental Medicine and Child Neurology*, *62*(6), 723-728. https://doi.org/10.1111/dmcn.14395 | No cognitive, Wrong study design |
| Characterization of iron accumulation in deep gray matter in myotonic dystrophy type 1 and 2 using quantitative susceptibility mapping and r2* relaxometry: A magnetic resonance imaging study at 3 tesla | 10.3389/fneur.2019.01320 | Ates, S., Deistung, A., Schneider, R., Prehn, C., Lukas, C., Reichenbach, J.R., Schneider-Gold, C. and Bellenberg, B. (2019). Characterization of iron accumulation in deep gray matter in myotonic dystrophy type 1 and 2 using quantitative susceptibility mapping and r2* relaxometry: A magnetic resonance imaging study at 3 tesla. *Frontiers in Neurology*, *10*. https://doi.org/10.3389/fneur.2019.01320 | Wrong study design |
| Pain in adult myotonic dystrophy type 1: Relation to function and gender | 10.1186/s12883-021-02124-9 | Solbakken, G., Løseth, S., Froholdt, A., Eikeland, T.D., Nærland, T., Frich, J.C., Dietrichs, E. and Ørstavik, K. (2021). Pain in adult myotonic dystrophy type 1: relation to function and gender. *BMC Neurology*, *21*(1). https://doi.org/10.1186/s12883-021-02124-9 | No cognitive |
| Hormonal and metabolic gender differences in a cohort of myotonic dystrophy type 1 subjects: a Retrospective, case–control study | 10.1007/s40618-019-01156-w | Spaziani, M., Semeraro, A., Bucci, E., Rossi, F., Garibaldi, M., Papassifachis, M.A., Pozza, C., Anzuini, A., Lenzi, A., Antonini, G. and Radicioni, A.F.(2020). Hormonal and metabolic gender differences in a cohort of myotonic dystrophy type 1 subjects: a retrospective, case–control study. *Journal of Endocrinological Investigation*, *43*(5), 663-675. https://doi.org/10.1007/s40618-019-01156-w | Wrong publication type |
| New developments in myotonic dystrophies from a multisystemic perspective | 10.1097/WCO.0000000000000964 | Montagnese, F. and Schoser, B.(2021). New developments in myotonic dystrophies from a multisystemic perspective. *Current Opinion in Neurology*, *34*(5), 738-747. https://doi.org/10.1097/WCO.0000000000000964 | No cognitive, Wrong publication type |
| Clinical guide for the diagnosis and follow-up of myotonic dystrophy type 1, MD1 or steinert's disease | 10.1016/j.nrl.2019.01.001 | Gutiérrez Gutiérrez, G., Díaz-Manera, J., Almendrote, M., Azriel, S., Eulalio Bárcena, J., Cabezudo García, P., Camacho Salas, A., Casanova Rodríguez, C., Cobo, A.M., Díaz Guardiola, P., Fernández-Torrón, R., Gallano Petit, M.P., García Pavía, P., Gómez Gallego, M., Gutiérrez Martínez, A.J., Jericó, I., Kapetanovic García, S., López de Munaín Arregui, A., Martorell, L., Morís de la Tassa, G., Moreno Zabaleta, R., Muñoz-Blanco, J.L., Olivar Roldán, J., Pascual Pascual, S.I., Peinado Peinado, R., Pérez, H., Poza Aldea, J.J., Rabasa, M., Ramos, A., Rosado Bartolomé, A., Rubio Pérez, M.Á., Urtizberea, J.A., Zapata-Wainberg, G. and Gutiérrez-Rivas, E. (2020). Clinical guide for the diagnosis and follow-up of myotonic dystrophy type 1, MD1 or Steinert's disease. *Neurologia*, *35*(3), 185-206. https://doi.org/10.1016/j.nrl.2019.01.001 | No cognitive, Wrong publication type |
| The myotonic dystrophy experience: a north american cross-sectional study | 10.1002/mus.26420 | Hagerman, K.A., Howe, S.J. and Heatwole, C.R. (2019). The myotonic dystrophy experience: a north american cross-sectional study. *Muscle and Nerve*, *59*(4), 457-464. https://doi.org/10.1002/mus.26420 | Wrong study design |
| Validity of the mini-bestest in adults with myotonic dystrophy type 1 | 10.1002/mus.26893 | Duchesne, E., Hébert, L.J., Mathieu, J., Côté, I., Roussel, M.-P. and Gagnon, C. (2020). Validity of the mini-bestest in adults with myotonic dystrophy type 1. *Muscle and Nerve*, *62*(1), 95-102. https://doi.org/10.1002/mus.26893 | No cognitive |
| Prevalence of sexual function alterations in Mexican subjects with type 1 (DM1) and type 2 (DM2) diabetes mellitus attending in a high specialty medical unit (UMAE) | Not found | Coronado Cordero, I.A., Escalante Pulido, J.M. and de la Rosa, A.J.C. (2014). Prevalence of sexual function alterations in mexican subjects with type 1 (DM1) and type 2 (DM2) diabetes mellitus attending in a high specialty medical unit (UMAE). *Gaceta Medica de México*, *150*, 107-113. | No DM1 |
| the heart and cardiac pacing in steinert disease |  | Nigro, G., Papa, A.A. and Politano, L. (2012). The heart and cardiac pacing in steinert disease. *Acta Myologica*, *31*, 110-116. | No cognitive, Wrong publication type |
| Coexistence of myotonic dystrophy and normal pressure hydrocephaly: Causative or incidental? | 10.4274/Tnd.29591 | Barcin, E., Gürbüz, M., Kizilay, F., Karaali, K. and Uysal, H. (2013). Coexistence of myotonic dystrophy and normal pressure hydrocephaly: causative or incidental?. *Turk Noroloji Dergisi*, *19*(2), 56-59. https://doi.org/10.4274/Tnd.29591 | No cognitive, Wrong study design |
| Fnirs evaluation during a phonemic verbal task reveals prefrontal hypometabolism in patients affected by myotonic dystrophy type 1 | 10.1016/j.clinph.2013.05.010 | Caliandro, P., Silvestri, G., Padua, L., Bianchi, M.L.E., Simbolotti, C., Russo, G., Masciullo, M. and Rossini, P.M. (2013). Fnirs evaluation during a phonemic verbal task reveals prefrontal hypometabolism in patients affected by myotonic dystrophy type 1. *Clinical Neurophysiology*, *124*(11), 2269-2276. https://doi.org/10.1016/j.clinph.2013.05.010 | No cognitive, Wrong study design |
| Steinert myotonic dystrophy: a Family case report and literature review | Not found | Flores-López, E.N., Tovilla-Ruiz, C.K., García-Padilla, E., Sandoval-Gutiérrez, R.B. and Álvarez-Torrecilla, L.C. (2014). Steinert myotonic dystrophy: A family case report and literature review. *Medicina Interna de Mexico*, *30*(2), 195-203. | No cognitive, Wrong publication type |
| "I know that you know that i know": neural substrates associated with social cognition deficits in DM1 patients | 10.1371/journal.pone.0156901 | Serra, L., Cercignani, M., Bruschini, M., Cipolotti, L., Mancini, M., Silvestri, G., Petrucci, A., Bucci, E., Antonini, G., Licchelli, L., Spanò, B., Giacanelli, M., Caltagirone, C., Meola, G. and Bozzali, M.(2016). "I know that you know that I know": Neural substrates associated with social cognition deficits in DM1 patients. *Plos ONE*, *11*(6). https://doi.org/10.1371/journal.pone.0156901 | Wrong study design |
| MRC centre for neuromuscular diseases 1st (1st december 2010), and 2nd (2nd may 2012) myotonic dystrophy workshops, london, uk and the myotonic dystrophy standards of care and national registry meeting, newcastle, uk july 2011 | 10.1016/j.nmd.2013.07.006 | Turner, C., Hilton-Jones, D., Lochmüller, H. and Hanna, M.G. (2013). Mrc centre for neuromuscular diseases 1st (1st december 2010), and 2nd (2nd may 2012) myotonic dystrophy workshops, London, UK and the myotonic dystrophy standards of care and national registry meeting, Newcastle, UK July 2011. *Neuromuscular Disorders*, *23*(12), 1069-1080. https://doi.org/10.1016/j.nmd.2013.07.006 | No cognitive, Wrong publication type |
| Feasibility of lung volume recruitment in early neuromuscular weakness: A comparison between amyotrophic lateral sclerosis, myotonic dystrophy, and postpolio syndrome | 10.1016/j.pmrj.2015.04.001 | Kaminska, M., Browman, F., Trojan, D.A., Genge, A., Benedetti, A. and Petrof, B.J. (2015). Feasibility of lung volume recruitment in early neuromuscular weakness: A comparison between amyotrophic lateral sclerosis, myotonic dystrophy, and postpolio syndrome. *PM and R*, *7*(7), 677-684. https://doi.org/10.1016/j.pmrj.2015.04.001 | No cognitive |
| Reduced cytoplasmic mbnl1 is an early event in a brain-specific mouse model of myotonic dystrophy | 10.1093/hmg/ddx115 | Wang, P.-Y., Lin, Y.-M., Wang, L.-H., Kuo, T.-Y., Cheng, S.-J. and Wang, G.-S. (2017). Reduced cytoplasmic mbnl1 is an early event in a brain-specific mouse model of myotonic dystrophy. *Human Molecular Genetics*, *26*(12), 2247-2257. https://doi.org/10.1093/hmg/ddx115 | Wrong study design |
| Myotonic dystrophies: Clinical presentation, pathogenesis, diagnostics and therapy | 10.1055/s-0034-1385734 | Finsterer, J. and Rudnik-Schöneborn, S. (2015). Myotonic dystrophies: clinical presentation, pathogenesis, diagnostics and therapy. *Fortschritte der Neurologie Psychiatrie*, *83*(1), 9-17. https://doi.org/10.1055/s-0034-1385734 | No cognitive |
| The proposal of a clinical protocol to assess central and peripheral fatigue in myotonic dystrophy type 1 | 10.12871/000398292017125 | Baldanzi, S., Ricci, G., Bottari, M., Chico, L., Simoncini, C. and Gabriele, S. (2017). The proposal of a clinical protocol to assess central and peripheral fatigue in myotonic dystrophy type 1. *Archives Italiennes de Biologie*, *155*(1), 43-53. https://doi.org/10.12871/000398292017125 | Wrong study design |
| Cortical and subcortical grey and white matter atrophy in myotonic dystrophies type 1 and 2 is associated with cognitive impairment, depression and daytime sleepiness | 10.1371/journal.pone.0130352 | Schneider-Gold, C., Bellenberg, B., Prehn, C., Krogias, C., Schneider, R., Klein, J., Gold, R. and Lukas, C.(2015). Cortical and subcortical grey and white matter atrophy in myotonic dystrophies type 1 and 2 is associated with cognitive impairment, depression and daytime sleepiness. *Plos ONE*, *10*(6). https://doi.org/10.1371/journal.pone.0130352 | Wrong study design |
| Effects of a balance exercise programme in myotonic dystrophy type 1: A pilot study | 10.3109/21679169.2015.1049204 | Hammarén, E., Lindberg, C. and Kjellby-Wendt, G. (2015). Effects of a balance exercise programme in myotonic dystrophy type 1: A pilot study. *European Journal of Physiotherapy*, *17*(3), 123-131. https://doi.org/10.3109/21679169.2015.1049204 | No cognitive |
| Prospective measurement of quality of life in myotonic dystrophy type 1 | 10.1111/ane.12788 | Peric, S., Heatwole, C., Durovic, E., Kacar, A., Nikolic, A., Basta, I., Marjanovic, A., Stevic, Z., Lavrnic, D. and Rakocevic Stojanovic, V. (2017). Prospective measurement of quality of life in myotonic dystrophy type 1. *Acta Neurologica Scandinavica*, *136*(6), 694-697. https://doi.org/10.1111/ane.12788 | No cognitive |
| Reliability of the apathy evaluation scale in myotonic dystrophy type 1 | 10.3233/JND-170274 | Gallais, B., Gagnon, C., Côté, I., Forgues, G. and Laberge, L. (2018). Reliability of the apathy evaluation scale in myotonic dystrophy type 1. *Journal of Neuromuscular Diseases*, *5*(1), 39-46. https://doi.org/10.3233/JND-170274 | No cognitive |
| Myotonic dystrophy: From bench to bedside | 10.1055/s-0032-1329202 | Johnson, N.E. and Heatwole, C.R. (2012). Myotonic dystrophy: from bench to bedside. *Seminars in Neurology*, *32*(3), 246-254. https://doi.org/10.1055/s-0032-1329202 | Wrong publication type |
| Medication adherence in patients with myotonic dystrophy and facioscapulohumeral muscular dystrophy | 10.1007/s00415-016-8300-3 | Fitzgerald, B.P., Conn, K.M., Smith, J., Walker, A., Parkhill, A.L., Hilbert, J.E., Luebbe, E.A. and Moxley, R.T. (2016). Medication adherence in patients with myotonic dystrophy and facioscapulohumeral muscular dystrophy. *Journal of Neurology*, *263*(12), 2528-2537. https://doi.org/10.1007/s00415-016-8300-3 | No cognitive |
| Report of the second outcome measures in myotonic dystrophy type 1 (ommyd-2) international workshop san sebastian, spain, october 16, 2013 | 10.1016/j.nmd.2015.01.008 | Gagnon, C., Meola, G., Hébert, L.J., Laberge, L., Leone, M. and Heatwole, C. (2015). Report of the second outcome measures in myotonic dystrophy type 1 (ommyd-2) international workshop san sebastian, spain, october 16, 2013. *Neuromuscular Disorders*, *25*(7), 603-616. https://doi.org/10.1016/j.nmd.2015.01.008 | Wrong publication type |
| Surgical treatment of obesity in DM1 - a case report and a review of the literature | 10.1016/j.nmd.2015.02.002 | Håkansson, K., Kostic, S. and Lindberg, C. (2015). Surgical treatment of obesity in DM1 - a case report and a review of the literature. *Neuromuscular Disorders*, *25*(5), 414-417. https://doi.org/10.1016/j.nmd.2015.02.002 | No cognitive, Wrong publication type |
| Report of the first outcome measures in myotonic dystrophy type 1 (ommyd-1) international workshop. clearwater, florida, november 30, 2011. | 10.1016/j.nmd.2013.07.004 | Gagnon, C., Meola, G., Hébert, L.J., Puymirat, J., Laberge, L. and Leone, M. (2013). Report of the first outcome measures in myotonic dystrophy type 1 (ommyd-1) international workshop. clearwater, florida, november 30, 2011. *Neuromuscular Disorders*, *23*(12), 1056-1068. https://doi.org/10.1016/j.nmd.2013.07.004 | Wrong publication type |
| Disease burden and functional outcomes in congenital myotonic dystrophy: a cross-sectional study | 10.1212/WNL.0000000000002845 | Johnson, N.E., Butterfield, R., Berggren, K., Hung, M., Chen, W., Dibella, D., Dixon, M., Hayes, H., Pucillo, E., Bounsanga, J., Heatwole, C. and Campbell, C.(2016). Disease burden and functional outcomes in congenital myotonic dystrophy: a cross-sectional study. *Neurology*, *87*(2), 160-167. https://doi.org/10.1212/WNL.0000000000002845 | Wrong publication type |
| Compound heterozygous myotonic dystrophy type 1 | 10.1002/ajmg.a.36798 | Finsterer, J. and Zarrouk-Mahjoub, S. (2015). Compound heterozygous myotonic dystrophy type 1. *American Journal of Medical Genetics*, *167*(8), 1952-1953. https://doi.org/10.1002/ajmg.a.36798 | No cognitive, Wrong study design |
| Abnormal functional brain connectivity and personality traits in myotonic dystrophy type 1 | 10.1001/jamaneurol.2014.130 | Serra, L., Silvestri, G., Petrucci, A., Basile, B., Masciullo, M., Makovac, E., Torso, M., Spanò, B., Mastropasqua, C., Harrison, N.A., Bianchi, M.L.E., Giacanelli, M., Caltagirone, C., Cercignani, M. and Bozzali, M. (2014). Abnormal functional brain connectivity and personality traits in myotonic dystrophy type 1. *JAMA Neurology*, *71*(5), 603-611. https://doi.org/10.1001/jamaneurol.2014.130 | Wrong study design |
| Clusters of cognitive impairment among different phenotypes of myotonic dystrophy type 1 and type 2 | 10.1007/s10072-016-2778-4 | Peric, S., Rakocevic Stojanovic, V., Mandic Stojmenovic, G., Ilic, V., Kovacevic, M., Parojcic, A., Pesovic, J., Mijajlovic, M., Savic-Pavicevic, D. and Meola, G. (2017). Clusters of cognitive impairment among different phenotypes of myotonic dystrophy type 1 and type 2. *Neurological Sciences*, *38*(3), 415-423. https://doi.org/10.1007/s10072-016-2778-4 | Wrong study design |
| Respiratory feature in myotonic dystrophy | 10.1007/978-981-13-0508-5_8 | Kuru, S.(2018). Respiratory feature in myotonic dystrophy. *Myotonic dystrophy: disease mechanism, current management and therapeutic development*, 133-147. https://doi.org/10.1007/978-981-13-0508-5_8 | No cognitive |
| Mtor-dependent proliferation defect in human es-derived neural stem cells affected by myotonic dystrophy type 1 | 10.1242/jcs.116285 | Denis, J.A., Gauthier, M., Rachdi, L., Aubert, S., Giraud-Triboult, K., Poydenot, P., Benchoua, A., Champon, B., Maury, Y., Baldeschi, C., Scharfmann, R., Piétu, G., Peschanski, M. and Martinat, C. (2013). Mtor-dependent proliferation defect in human es-derived neural stem cells affected by myotonic dystrophy type 1. *Journal of Cell Science*, *126*(8), 1763-1772. https://doi.org/10.1242/jcs.116285 | No cognitive |
| Brain positron emission tomography in patients with myotonic dystrophy type 1 and type 2 | 10.1016/j.jns.2017.05.013 | Peric, S., Brajkovic, L., Belanovic, B., Ilic, V., Salak-Djokic, B., Basta, I. and Rakocevic Stojanovic, V .(2017). Brain positron emission tomography in patients with myotonic dystrophy type 1 and type 2. *Journal of the Neurological Sciences*, *378*, 187-192. https://doi.org/10.1016/j.jns.2017.05.013 | No cognitive, Wrong study design |
| Current progress in cns imaging of myotonic dystrophy | 10.3389/fneur.2018.00646 | Minnerop, M., Gliem, C. and Kornblum, C. (2018). Current progress in cns imaging of myotonic dystrophy. *Frontiers in Neurology*, *9*. https://doi.org/10.3389/fneur.2018.00646 | No cognitive, Wrong publication type |
| Relationship between neuropsychological impairment and grey and white matter changes in adult-onset myotonic dystrophy type 1 | 10.1016/j.nicl.2016.06.011 | Baldanzi, S., Cecchi, P., Fabbri, S., Pesaresi, I., Simoncini, C., Angelini, C., Bonuccelli, U., Cosottini, M. and Siciliano, G. (2016). Relationship between neuropsychological impairment and grey and white matter changes in adult-onset myotonic dystrophy type 1. *Neuroimage: Clinical*, *12*, 190-197. https://doi.org/10.1016/j.nicl.2016.06.011 | Wrong study design |
| Cognitive behavioural therapy with optional graded exercise therapy in patients with severe fatigue with myotonic dystrophy type 1: a multicentre, single-blind, randomised trial | 10.1016/s1474-4422(18)30203-5 | Okkersen, K., Jimenez-Moreno, C., Wenninger, S., Daidj, F., Glennon, J., Cumming, S., Littleford, R., Monckton, D.G., Lochmüller, H., Catt, M., Faber, C.G., Hapca, A., Donnan, P.T., Gorman, G., Bassez, G., Schoser, B., Knoop, H., Treweek, S., van Engelen, B.G.M., Kierkegaard, M., Wenninger, S., Lochmüller, H., Schoser, B., Treweek, S., Maas, D., Nikolaus, S., Cornelissen, Y., van Nimwegen, M., Klerks, E., Bouman, S., Heskamp, L., Heerschap, A., Rahmadi, R., Groot, P., Heskes, T., Kapusta, K., Abghari, S., Aschrafi, A., Poelmans, G., Raaphorst, J., Trenell, M., van Laar, S., Wood, L., Cassidy, S., Newman, J., Charman, S., Steffaneti, R., Taylor, L., Brownrigg, A., Day, S., Atalaya, A., Hogarth, F., Schüller, A., Stahl, K., Künzel, H., Wolf, M., Jelinek, A., Lignier, B., Couppey, F., Delmas, S., Deux, J.-F., Hankiewicz, K., Dogan, C., Minier, L., Chevalier, P., Hamadouche, A., Adam, B., Hannah, M., McKenzie, E., Rauchhaus, P., Van Hees, V., Catt, S., Schwalber, A., Merkies, I. and Dittrich, J.(2018). Cognitive behavioural therapy with optional graded exercise therapy in patients with severe fatigue with myotonic dystrophy type 1: A multicentre, single-blind, randomised trial. *The Lancet Neurology*, *17*(8), 671-680. https://doi.org/10.1016/s1474-4422(18)30203-5 | No cognitive |
| Myotonic dystrophies: state of the art of new therapeutic developments for the cns | 10.3389/fncel.2017.00101 | Gourdon, G. and Meola, G. (2017). Myotonic dystrophies: state of the art of new therapeutic developments for the cns. *Frontiers in Cellular Neuroscience*, 11. https://doi.org/10.3389/fncel.2017.00101 | No cognitive, Wrong publication type |
| Review of patient-reported outcome measures for use in myotonic dystrophy type 1 patients | 10.1002/mus.25469 | Symonds, T., Randall, J.A. and Campbell, P. (2017). Review of patient-reported outcome measures for use in myotonic dystrophy type 1 patients. *Muscle and Nerve*, *56*(1), 86-92. https://doi.org/10.1002/mus.25469 | Wrong publication type |
| Muscle force, balance and falls in muscular impaired individuals with Myotonic dystrophy type 1: A five-year prospective cohort study | 10.1016/j.nmd.2014.11.004 | Hammarén, E., Kjellby-Wendt, G. and Lindberg, C. (2015). Muscle force, balance and falls in muscular impaired individuals with myotonic dystrophy type 1: a five-year prospective cohort study. *Neuromuscular Disorders*, *25*(2), 141-148. https://doi.org/10.1016/j.nmd.2014.11.004 | No cognitive |
| How genetics affects the brain to produce higher-level dysfunctions in myotonic dystrophy type 1 | 10.11138/FNeur/2015.30.1.021 | Serra, L., Petrucci, A., Spanò, B., Torso, M., Olivito, G., Lispi, L., Costanzi-Porrini, S., Giulietti, G., Koch, G., Giacanelli, M., Caltagirone, C., Cercignani, M. and Bozzali, M. (2015). How genetics affects the brain to produce higher-level dysfunctions in myotonic dystrophy type 1. *Functional neurology*, *30*(1), 21-31. https://doi.org/10.11138/FNeur/2015.30.1.021 | Wrong study design |
| Brain connectomics' modification to clarify motor and nonmotor features of myotonic dystrophy type 1 | 10.1155/2016/2696085 | Serra, L., Mancini, M., Silvestri, G., Petrucci, A., Masciullo, M., Spanò, B., Torso, M., Mastropasqua, C., Giacanelli, M., Caltagirone, C., Cercignani, M., Meola, G. and Bozzali, M. (2016). Brain connectomics' modification to clarify motor and nonmotor features of myotonic dystrophy type 1. *Neural Plasticity*. https://doi.org/10.1155/2016/2696085 | Wrong publication type |
| Significant impact of behavioral and cognitive impairment on quality of life in patients with myotonic dystrophy type 1 | 10.1016/j.clineuro.2014.08.021 | Rakocevic-Stojanovic, V., Peric, S., Madzarevic, R., Dobricic, V., Ralic, V., Ilic, V., Basta, I., Nikolic, A. and Stefanova, E.(2014). Significant impact of behavioral and cognitive impairment on quality of life in patients with myotonic dystrophy type 1. *Clinical Neurology and Neurosurgery*, *126*, 76-81. https://doi.org/10.1016/j.clineuro.2014.08.021 | Wrong publication type |
| Molecular, clinical, and muscle studies in myotonic dystrophy type 1 (DM1) associated with novel variant CCG expansions | 10.1007/s00415-012-6779-9 | Santoro, M., Masciullo, M., Pietrobono, R., Conte, G., Modoni, A., Bianchi, M.L.E., Rizzo, V., Pomponi, M.G., Tasca, G., Neri, G. and Silvestri, G.(2013). Molecular, clinical, and muscle studies in myotonic dystrophy type 1 (DM1) associated with novel variant CCG expansions. *Journal of Neurology*, *260*(5), 1245-1257. https://doi.org/10.1007/s00415-012-6779-9 | No cognitive, Wrong publication type |
| Myotonic dystrophy | 10.1016/B978-0-12-417044-5.00037-8 | Moxley, R.T., Ciafaloni, E. and Guntrum, D. (2015). Myotonic dystrophy. *Neuromuscular Disorders of Infancy, Childhood, and Adolescence: A Clinician's Approach*, 697-718. https://doi.org/10.1016/B978-0-12-417044-5.00037-8 | Wrong publication type |
| Prevalence and correlates of apathy in myotonic dystrophy type 1 | 10.1186/s12883-015-0401-6 | Gallais, B., Montreuil, M., Gargiulo, M., Eymard, B., Gagnon, C. and Laberge, L. (2015). Prevalence and correlates of apathy in myotonic dystrophy type 1. *BMC Neurology*, *15*(1). https://doi.org/10.1186/s12883-015-0401-6 | Wrong study design |
| Assessing upper extremity capacity as a potential indicator of needs related to household activities for rehabilitation services in people with myotonic dystrophy type 1 | 10.1016/j.nmd.2015.03.015 | Raymond, K., Auger, L.-P., Cormier, M.-F., Vachon, C., St-Onge, S., Mathieu, J., Noreau, L. and Gagnon, C. (2015). Assessing upper extremity capacity as a potential indicator of needs related to household activities for rehabilitation services in people with myotonic dystrophy type 1. *Neuromuscular Disorders*, *25*(6), 522-529. https://doi.org/10.1016/j.nmd.2015.03.015 | No cognitive, Wrong study design |
| Ocular findings of myotonic dystrophy type 1 in the Korean population | 10.1007/s00417-016-3266-5 | Choi, S.H., Yang, H.K., Hwang, J.-M. and Park, K.S. (2016). Ocular findings of myotonic dystrophy type 1 in the korean population. *Graefe's Archive for Clinical and Experimental Ophthalmology*, *254*(6), 1189-1193. https://doi.org/10.1007/s00417-016-3266-5 | No cognitive |
| Myotonic dystrophy - unity in diversity | 10.14735/amcsnn2017255 | Voháňka, S.(2017). Myotonic dystrophy - unity in diversity. *Ceska a Slovenska Neurologie a Neurochirurgie*, *80*(3), 255-265. https://doi.org/10.14735/amcsnn2017255 | No cognitive, Wrong publication type |
| Responsiveness of performance-based outcome measures for mobility, balance, muscle strength and manual dexterity in adults with myotonic dystrophy type 1 | 10.2340/16501977-2304 | Kierkegaard, M., Petitclerc, É., Hébert, L.J., Mathieu, J. and Gagnon, C.(2018). Responsiveness of performance-based outcome measures for mobility, balance, muscle strength and manual dexterity in adults with myotonic dystrophy type 1. *Journal of Rehabilitation Medicine*, *50*(3), 269-277. https://doi.org/10.2340/16501977-2304 | No cognitive |
| Mitochondrial dysfunction in myotonic dystrophy type 1 | 10.1016/j.nmd.2017.10.007 | Gramegna, L.L., Giannoccaro, M.P., Manners, D.N., Testa, C., Zanigni, S., Evangelisti, S., Bianchini, C., Oppi, F., Poda, R., Avoni, P., Lodi, R., Liguori, R. and Tonon, C. (2018). Mitochondrial dysfunction in myotonic dystrophy type 1. *Neuromuscular Disorders*, *28*(2), 144-149. https://doi.org/10.1016/j.nmd.2017.10.007 | No cognitive |
| Altered power spectral density in the resting-state sensorimotor network in patients with myotonic dystrophy type | 10.1038/s41598-018-19217-0 | Park, J.-S., Seo, J., Cha, H., Song, H.-J., Lee, S.-H., Jang, K.E., Lee, H.J., Park, J., Lee, H.-W. and Chang, Y.(2018). Altered power spectral density in the resting-state sensorimotor network in patients with myotonic dystrophy type. *Scientific Reports*, 8(1). https://doi.org/10.1038/s41598-018-19217-0 | No cognitive |
| Is one trial enough for repeated testing? Same-day assessments of walking, mobility and fine hand use in people with myotonic dystrophy type 1 | 10.1016/j.nmd.2016.11.018 | Kierkegaard, M., Petitclerc, E., Hébert, L.J. and Gagnon, C. (2017). Is one trial enough for repeated testing? Same-day assessments of walking, mobility and fine hand use in people with myotonic dystrophy type 1. *Neuromuscular Disorders*, 27(2), 153-158. https://doi.org/10.1016/j.nmd.2016.11.018 | No cognitive |
| Delusional and psychotic disorders in juvenile myotonic dystrophy type-1 | 10.1002/ajmg.b.32524 | Jacobs, D., Willekens, D., de Die-Smulders, C., Frijns, J.-P. and Steyaert, J.(2017). Delusional and psychotic disorders in juvenile myotonic dystrophy type-1. *American Journal of Medical Genetics, part b: Neuropsychiatric Genetics*, *174*(4), 359-366. https://doi.org/10.1002/ajmg.b.32524 | No cognitive |
| Frontostriatal dysexecutive syndrome: a core cognitive feature of myotonic dystrophy type 2 | 10.1007/s00415-014-7545-y | Peric, S., Mandic-Stojmenovic, G., Stefanova, E., Savic-Pavicevic, D., Pesovic, J., Ilic, V., Dobricic, V., Basta, I., Lavrnic, D. and Rakocevic-Stojanovic, V. (2015). Frontostriatal dysexecutive syndrome: a core cognitive feature of myotonic dystrophy type 2. *Journal of Neurology*, *262*(1), 142-148. https://doi.org/10.1007/s00415-014-7545-y | No DM1 |
| RAN translation regulated by muscleblind proteins in myotonic dystrophy type 2 | 10.1016/j.neuron.2017.08.039 | Zu, T., Cleary, J.D., Liu, Y., Bañez-Coronel, M., Bubenik, J.L., Ayhan, F., Ashizawa, T., Xia, G., Clark, H.B., Yachnis, A.T., Swanson, M.S. and Ranum, L.P.W. (2017). Ran translation regulated by muscleblind proteins in myotonic dystrophy type 2. *Neuron*, *95*(6), 1292-1305.e5. https://doi.org/10.1016/j.neuron.2017.08.039 | No DM1 |
| Fatigue in steinert myotonic dystrophy: literature review and research perspectives | 10.1016/j.amp.2011.02.017 | Gallais, B., Gargiulo, M., Montreuil, M. and Eymard, B. (2013). Fatigue in steinert myotonic dystrophy: literature review and research perspectives. *Annales Medico-Psychologiques*, *171*(2), 83-88. https://doi.org/10.1016/j.amp.2011.02.017 | No cognitive, Wrong publication type |
| Social cognition in myotonic dystrophy type 1: specific or secondary impairment? | 10.1371/journal.pone.0204227 | Labayru, G., Arenzana, I., Aliri, J., Zulaica, M., Lopez De Munain, A. and Andone Sistiaga, A. (2018). Social cognition in myotonic dystrophy type 1: specific or secondary impairment? *Plos ONE*, *13*(9). https://doi.org/10.1371/journal.pone.0204227 | Wrong study design |
| 207th ENMC workshop on chronic respiratory insufficiency in myotonic dystrophies: management and implications for research, 27-29 june 2014, naarden, the netherlands | 10.1016/j.nmd.2015.01.011 | Sansone, V.A., Gagnon, C., Atalaia , A., Boentert, M., Duboc, D., Falcier, E., Gallais, B., Heatwole, C., Iatomasi, M., Orlikowski, D., Phillips, M., Rao, F., Rogers, M., Schoser, B. and Wijkstra, P.(2015). 207th ENMC workshop on chronic respiratory insufficiency in myotonic dystrophies: management and implications for research, 27-29 june 2014, naarden, the netherlands. *Neuromuscular Disorders*, *25*(5), 432-442. https://doi.org/10.1016/j.nmd.2015.01.011 | No cognitive |
| Steinert syndrome and repercussions in dental medicine | 10.1016/j.archoralbio.2016.12.008 | Baptista, H. and Lopes Cardoso, I. (2017). Steinert syndrome and repercussions in dental medicine. *Archives of Oral Biology*, *75*, 37-47. https://doi.org/10.1016/j.archoralbio.2016.12.008 | No cognitive |
| The cognitive profile of myotonic dystrophy type 1: A systematic review and meta-analysis | 10.1016/j.cortex.2017.08.008 | Okkersen, K., Buskes, M., Groenewoud, J., Kessels, R.P.C., Knoop, H., van Engelen, B. and Raaphorst, J. (2017). The cognitive profile of myotonic dystrophy type 1: A systematic review and meta-analysis. *Cortex*, *95*, 143-155. https://doi.org/10.1016/j.cortex.2017.08.008 | Wrong publication type |
| Diffuse brain abnormalities in myotonic dystrophy type 1 detected by 3.0 t proton magnetic resonance spectroscopy | 10.1159/000371575 | Takado, Y., Terajima, K., Ohkubo, M., Okamoto, K., Shimohata, T., Nishizawa, M., Igarashi, H. and Nakada, T. (2015). Diffuse brain abnormalities in myotonic dystrophy type 1 detected by 3.0 T proton magnetic resonance spectroscopy. *European Neurology*, *73*(3), 247-256. https://doi.org/10.1159/000371575 | No cognitive |
| Muscle wasting in myotonic dystrophies: A model of premature aging | 10.3389/fnagi.2015.00125 | Mateos-Aierdi, A.J., Goicoechea, M., Aiastui, A., Torrón, R.F., Garcia-Puga, M., Matheu, A. and de Munain, A.L. (2015). Muscle wasting in myotonic dystrophies: a model of premature aging. *Frontiers in Aging Neuroscience*, 7. https://doi.org/10.3389/fnagi.2015.00125 | No cognitive, Wrong publication type |
| Diagnostic odyssey of patients with myotonic dystrophy | 10.1007/s00415-013-6993-0 | Hilbert, J.E., Ashizawa, T., Day, J.W., Luebbe, E.A., Martens, W.B., McDermott, M.P., Tawil, R., Thornton, C.A. and Moxley III, R.T. (2013). Diagnostic odyssey of patients with myotonic dystrophy. *Journal of Neurology*, *260*(10), 2497-2504. https://doi.org/10.1007/s00415-013-6993-0 | No cognitive |
| Factors influencing compliance with non-invasive ventilation at long-term in patients with myotonic dystrophy type 1: A prospective cohort | 10.1016/j.nmd.2016.07.014 | Boussaïd, G., Lofaso, F., Santos, D.B., Vaugier, I., Pottier, S., Prigent, H., Orlikowski, D. and Bahrami, S. (2016). Factors influencing compliance with non-invasive ventilation at long-term in patients with myotonic dystrophy type 1: a prospective cohort. *Neuromuscular Disorders*, *26*(10), 666-674. https://doi.org/10.1016/j.nmd.2016.07.014 | No cognitive |
| Central nervous system involvement in myotonic dystrophies | Not found | Silvestri, G., Modoni, A., Bianchi, M.L.E. and Marra, C. (2015). Central nervous system involvement in myotonic dystrophies. *Myotonic Dystrophies: epidemiology, diagnosis and therapeutic challenges*, 47-64. | Wrong publication type |
| Generalized epilepsy in a patient with myotonic dystrophy type 2: A case report | Not found | Cagnetti, C., Buratti, L., Balestrini, S., Foschi, N., Logullo, F., Petrelli, C. and Provinciali, L. (2013). Generalized epilepsy in a patient with myotonic dystrophy type 2: a case report. *Bollettino - Lega Italiana Contro l'Epilessia*, (145), 125-127. | No DM1,No cognitive, Wrong publication type |
| Brain involvement in myotonic dystrophy type 1: A morphometric and diffusion tensor imaging study with neuropsychological correlation | 10.1093/arclin/acx008 | Cabada, T., Iridoy, M., Jericó, I., Lecumberri, P., Seijas, R., Gargallo, A. and Gomez, M. (2017). Brain involvement in myotonic dystrophy type 1: a morphometric and diffusion tensor imaging study with neuropsychological correlation. *Archives of Clinical Neuropsychology*, *32*(4), 401-412. https://doi.org/10.1093/arclin/acx008 | Wrong study design |
| Cognitive deficits associated with sleep apnea in myotonic dystrophy type 1 | 10.3233/JND-140012 | Gallais, B., Gagnon, C., Mathieu, J., Richer, L., Jean, S. and Laberge, L. (2014). Cognitive deficits associated with sleep apnea in myotonic dystrophy type 1. *Journal of Neuromuscular Diseases*, *1*(1), 95-98. https://doi.org/10.3233/JND-140012 | No cognitive, Wrong study design |
| Lower limb muscle impairment in myotonic dystrophy type 1: The need for better guidelines | 10.1002/mus.24521 | Petitclerc, E., Hébert, L.J., Desrosiers, J. and Gagnon, C. (2015). Lower limb muscle impairment in myotonic dystrophy type 1: the need for better guidelines. *Muscle and Nerve*, *51*(4), 473-478. https://doi.org/10.1002/mus.24521 | No cognitive, Wrong publication type |
| Brain gray matter structural network in myotonic dystrophy type 1 | 10.1371/journal.pone.0187343 | Sugiyama, A., Sone, D., Sato, N., Kimura, Y., Ota, M., Maikusa, N., Maekawa, T., Enokizono, M., Mori-Yoshimura, M., Ohya, Y., Kuwabara, S. and Matsuda, H.(2017). Brain gray matter structural network in myotonic dystrophy type 1. *Plos ONE*, *12*(11). https://doi.org/10.1371/journal.pone.0187343 | Wrong study design |
| Cognitive impairment and quality of life in patients with myotonic dystrophy type 1 | 10.1002/mus.26022 | Fujino, H., Shingaki, H., Suwazono, S., Ueda, Y., Wada, C., Nakayama, T., Takahashi, M.P., Imura, O. and Matsumura, T.(2018). Cognitive impairment and quality of life in patients with myotonic dystrophy type 1. *Muscle and Nerve*, *57*(5), 742-748. https://doi.org/10.1002/mus.26022 | Wrong study design |
| Myotonic dystrophies | 10.1016/B978-0-12-383834-6.00139-7 | Turner, C.(2013). Myotonic dystrophies. *Emery and rimoin's principles and practice of medical genetics*, 1-30. https://doi.org/10.1016/B978-0-12-383834-6.00139-7 | Wrong publication type |
| Modeling of myotonic dystrophy cardiac phenotypes in drosophila | 10.3389/fneur.2018.00473 | Chakraborty, M., Llamusi, B. and Artero, R.(2018). Modeling of myotonic dystrophy cardiac phenotypes in drosophila. *Frontiers in Neurology*, *9*. https://doi.org/10.3389/fneur.2018.00473 | No cognitive, Wrong publication type |
| Core clinical phenotypes in myotonic dystrophies | 10.3389/fneur.2018.00303 | Wenninger, S., Montagnese, F. and Schoser, B. (2018). Core clinical phenotypes in myotonic dystrophies. *Frontiers in Neurology*, *9*. https://doi.org/10.3389/fneur.2018.00303 | No cognitive, Wrong publication type |
| Neuropsychological and psychological functioning aspects in myotonic dystrophy type 1 patients in italy | 10.3389/fneur.2018.00751 | Callus, E., Bertoldo, E.G., Beretta, M., Boveri, S., Cardani, R., Fossati, B., Brigonzi, E. and Meola, G. (2018). Neuropsychological and psychological functioning aspects in myotonic dystrophy type 1 patients in Italy. *Frontiers in Neurology*, 9. https://doi.org/10.3389/fneur.2018.00751 | Wrong publication type |
| Of mice and men: advances in the understanding of neuromuscular aspects of myotonic dystrophy | 10.3389/fneur.2018.00519 | Braz, S.O., Acquaire, J., Gourdon, G. and Gomes-Pereira, M. (2018). Of mice and men: Advances in the understanding of neuromuscular aspects of myotonic dystrophy. *Frontiers in Neurology*, *9*. https://doi.org/10.3389/fneur.2018.00519 | No cognitive, Wrong publication type |
| A descriptive study of the neuropsychological and psychopathological profile in patients with type 1 myotonic dystrophy | 10.33588/rn.6112.2015299 | Seijas-Gómez, R., Basterra-Jiménez, I., Luna-Lario, P., Tirapu-Ustárroz, J., Cabada-Giadás, T., Iridoy-Zulet, M., Jericó-Pascual, I., Gargallo-Vaamonde, Á. and López-Goñi, J.J. (2015). A descriptive study of the neuropsychological and psychopathological profile in patients with type 1 myotonic dystrophy. *Revista de Neurologia*, *61*(12), 529-535. https://doi.org/10.33588/rn.6112.2015299 | Wrong study design |
| Is the epicardial left ventricular lead implantation an alternative approach to percutaneous attempt in patients with steinert disease? A case report | Not found | Papa, A.A., Rago, A., Petillo, R., D'Ambrosio, P., Scutifero, M., Feo, M.D., Maiello, C. and Palladino, A. (2017). Is the epicardial left ventricular lead implantation an alternative approach to percutaneous attempt in patients with steinert disease? a case report. *Acta Myologica*, *36*(4), 213-217. | No cognitive, Wrong publication type |
| Sleepiness and sleep-related breathing disorders in myotonic dystrophy and responses to treatment: A prospective cohort study | 10.3233/JND-160191 | West, S.D., Lochmüller, H., Hughes, J., Atalaia, A., Marini-Bettolo, C., Baudouin, S.V. and Anderson, K.N. (2016). Sleepiness and sleep-related breathing disorders in myotonic dystrophy and responses to treatment: a prospective cohort study. *Journal of Neuromuscular Diseases*, *3*(4), 529-537. https://doi.org/10.3233/JND-160191 | No cognitive, Wrong study design |
| Cortical thickness and white matter integrity are associated with CTG expansion size in myotonic dystrophy type I | 10.3349/ymj.2017.58.4.807 | Yoo, W.-K., Park, Y.G., Choi, Y.C. and Kim, S.M. (2017). Cortical thickness and white matter integrity are associated with CTG expansion size in myotonic dystrophy type I. *Yonsei Medical Journal*, *58*(4), 807-815. https://doi.org/10.3349/ymj.2017.58.4.807 | No cognitive |
| Magnetic resonance of rare inherited neurological diseases - congenital cataracts, facial dysmorphism neuropathy syndrome and myotonic dystrophy type 1 and type 2 | Not found | Zlatareva, D., Tournev, I., Chamova, T. and Hadjidekov, V. (2013). Magneticresonance of rare inherited neurological diseases - congenital cataracts, facial dysmorphism neuropathy syndrome and myotonic dystrophy type 1 and type 2. *Rentgenologiya i Radiologiya*, *52*(3), 171-177. | No cognitive, Wrong publication type |
| A 9-year follow-up study of quantitative muscle strength changes in myotonic dystrophy type 1 | 10.1007/s00415-018-8898-4 | Gagnon, C., Petitclerc, É., Kierkegaard, M., Mathieu, J., Duchesne, É. and Hébert, L.J. (2018). A 9-year follow-up study of quantitative muscle strength changes in myotonic dystrophy type 1. *Journal of Neurology*, *265*(7), 1698-1705. https://doi.org/10.1007/s00415-018-8898-4 | No cognitive |
| Muscleblind-like 2-mediated alternative splicing in the developing brain and dysregulation in myotonic dystrophy | 10.1016/j.neuron.2012.05.029 | Charizanis, K., Lee, K.-Y., Batra, R., Goodwin, M., Zhang, C., Yuan, Y., Shiue, L., Cline, M., Scotti, M.M., Xia, G., Kumar, A., Ashizawa, T., Clark, H.B., Kimura, T., Takahashi, M.P., Fujimura, H., Jinnai, K., Yoshikawa, H., Gomes-Pereira, M., Gourdon, G., Sakai, N., Nishino, S., Foster, T.C., Ares, M., Darnell, R.B. and Swanson, M.S.(2012). Muscleblind-like 2-mediated alternative splicing in the developing brain and dysregulation in myotonic dystrophy. *Neuron*, *75*(3), 437-450. https://doi.org/10.1016/j.neuron.2012.05.029 | No cognitive |
| Brain MRI abnormalities in the adult form of myotonic dystrophy type 1: A longitudinal case series study | 10.1177/1971400915621325 | Conforti, R., De Cristofaro, M., Cristofano, A., Brogna, B., Sardaro, A., Tedeschi, G., Cirillo, S. and Di Costanzo, A. (2016). Brain mri abnormalities in the adult form of myotonic dystrophy type 1: A longitudinal case series study. *Neuroradiology journal*, *29*(1), 36-45. https://doi.org/10.1177/1971400915621325 | No cognitive |
| Myotonic dystrophy type 1: role of CCG, CTC and CGG interruptions within DMPK alleles in the pathogenesis and molecular diagnosis | 10.1111/cge.12954 | Santoro, M., Masciullo, M., Silvestri, G., Novelli, G., Botta, A.(2017). Myotonic dystrophy type 1: role of CCG, CTC and CGG interruptions within DMPK alleles in the pathogenesis and molecular diagnosis. *Clinical Genetics*, *92*(4), 355-364. https://doi.org/10.1111/cge.12954 | No cognitive, Wrong publication type |
| The myotonic dystrophy health index: Italian validation of a disease-specific outcome measure | 10.1016/j.nmd.2017.07.004 | Sansone, V.A., Lizio, A., Greco, L., Gragnano, G., Zanolini, A., Gualandris, M., Iatomasi, M. and Heatwole, C. (2017). The myotonic dystrophy health index: Italian validation of a disease-specific outcome measure. *Neuromuscular Disorders*, *27*(11), 1047-1053. https://doi.org/10.1016/j.nmd.2017.07.004 | Wrong publication type |
| Brain imaging in myotonic dystrophy type 1 | 10.1212/WNL.0000000000004300 | Okkersen, K., Monckton, D.G., Le, N., Tuladhar, A.M., Raaphorst, J. and Van Engelen, B.G.M. (2017). Brain imaging in myotonic dystrophy type 1. *Neurology*, *89*(9), 960-969. https://doi.org/10.1212/WNL.0000000000004300 | Wrong publication type |
| Brain tumors in patients with myotonic dystrophy: a population-based study | 10.1111/ene.12886 | Gadalla, S.M., Pfeiffer, R.M., Kristinsson, S.Y., Björkholm, M., Landgren, O. and Greene, M.H. (2016). Brain tumors in patients with myotonic dystrophy: A population-based study. *European Journal of Neurology*, *23*(3), 542-547. https://doi.org/10.1111/ene.12886 | No cognitive |
| Variant interrupted DMPK alleles: implications in the pathogenesis and molecular diagnosis of myotonic dystrophy type 1 (DM1) | Not found | Massimo, S., Marcella, M., Giuseppe, N. and Annalisa, B.(2015). Variant interrupted DMPK alleles: implications in the pathogenesis and molecular diagnosis of myotonic dystrophy type 1 (DM1). *Myotonic dystrophies: epidemiology, diagnosis and therapeutic challenges*, , , 121-137. | No cognitive, Wrong publication type |
| Myotonic dystrophy type 1 management and therapeutics | 10.1007/s11940-016-0434-1 | Smith, C.A. and Gutmann, L. (2016). Myotonic dystrophy type 1 management and therapeutics.*Current Treatment Options in Neurology*, *18*(12), . https://doi.org/10.1007/s11940-016-0434-1 | No cognitive |
| A genetic systemic disease: clinical description of type 1 myotonic dystrophy in adults | 10.1016/j.revmed.2012.03.355 | Kaminsky, P. and Pruna, L. (2012). A genetic systemic disease: clinical description of type 1 myotonic dystrophy in adults. *Revue de Medecine Interne*, *33*(9), 514-518. https://doi.org/10.1016/j.revmed.2012.03.355 | No cognitive |
| Outcome measures for central nervous system evaluation in myotonic dystrophy type 1 may be confounded by deficits in motor function or insight | 10.3389/fneur.2018.00780 | Hamilton, M.J., McLean, J., Cumming, S., Ballantyne, B., McGhie, J., Jampana, R., Longman, C., Evans, J.J., Monckton, D.G. and Farrugia, M.E. (2018). Outcome measures for central nervous system evaluation in myotonic dystrophy type 1 may be confounded by deficits in motor function or insight. *Frontiers in Neurology*, 9. https://doi.org/10.3389/fneur.2018.00780 | Wrong publication type |
| CPG methylation, a parent-of-origin effect for maternal-biased transmission of congenital myotonic dystrophy | 10.1016/j.ajhg.2017.01.033 | Barbé, L., Lanni, S., López-Castel, A., Franck, S., Spits, C., Keymolen, K., Seneca, S., Tomé, S., Miron, I., Letourneau, J., Liang, M., Choufani, S., Weksberg, R., Wilson, M.D., Sedlacek, Z., Gagnon, C., Musova, Z., Chitayat, D., Shannon, P., Mathieu, J., Sermon, K. and Pearson, C.E. (2017). CPG methylation, a parent-of-origin effect for maternal-biased transmission of congenital myotonic dystrophy. *American Journal of Human Genetics*, *100*(3), 488-505. https://doi.org/10.1016/j.ajhg.2017.01.033 | No cognitive |
| Five-year study of quality of life in myotonic dystrophy | 10.1111/ane.12549 | Peric, S., Vujnic, M., Dobricic, V., Marjanovic, A., Basta, I., Novakovic, I., Lavrnic, D. and Rakocevic-Stojanovic, V. (2016). Five-year study of quality of life in myotonic dystrophy. *Acta Neurologica Scandinavica*, *134*(5), 346-351. https://doi.org/10.1111/ane.12549 | No cognitive |
| Validation of the nine hole peg test as a measure of dexterity in myotonic dystrophy type 1 | 10.1016/j.nmd.2018.08.011 | Cutellè, C., Rastelli, E., Gibellini, M., Greco, G., Frezza, E., Botta, A., Terracciano, C. and Massa, R. (2018). Validation of the nine hole peg test as a measure of dexterity in myotonic dystrophy type 1. *Neuromuscular Disorders*, *28*(11), 947-951. https://doi.org/10.1016/j.nmd.2018.08.011 | No cognitive |
| A new window on neurocognitive dysfunction in the childhood form of myotonic dystrophy type 1 (DM1) | 10.1016/j.nmd.2011.04.009 | Angeard, N., Jacquette, A., Gargiulo, M., Radvanyi, H., Moutier, S., Eymard, B. and Héron, D. (2011). A new window on neurocognitive dysfunction in the childhood form of myotonic dystrophy type 1 (DM1). *Neuromuscular Disorders*, *21*(7), 468-476. https://doi.org/10.1016/j.nmd.2011.04.009 | Wrong study design |
| The brain in myotonic dystrophy 1 and 2: Evidence for a predominant white matter disease | 10.1093/brain/awr299 | Minnerop, M., Weber, B., Schoene-Bake, J.-C., Roeske, S., Mirbach, S., Anspach, C., Schneider-Gold, C., Betz, R.C., Helmstaedter, C., Tittgemeyer, M., Klockgether, T. and Kornblum, C. (2011). The brain in myotonic dystrophy 1 and 2: Evidence for a predominant white matter disease. *Brain*, *134*(12), 3527-3543. https://doi.org/10.1093/brain/awr299 | No cognitive, Wrong study design |
| Development of a disability scale for myotonic dystrophy type 1 | 10.1111/j.1600-0404.2011.01587.x | Contardi, S., Pizza, F., Falzone, F., D'Alessandro, R., Avoni, P., Di Stasi, V., Montagna, P. and Liguori, R. (2012). Development of a disability scale for myotonic dystrophy type 1. *Acta Neurologica Scandinavica*, *125*(6), 431-438. https://doi.org/10.1111/j.1600-0404.2011.01587.x | No cognitive,Wrong study design |
| Magnetic resonance imaging of muscle and brain in myotonic dystrophy | 10.1007/BF00838438 | Damian, M.S., Bachmann, G., Herrmann, D. and Dorndorf, W. (1993). Magnetic resonance imaging of muscle and brain in myotonic dystrophy. *Journal of neurology*, *240*(1), 8-12. https://doi.org/10.1007/BF00838438 | No cognitive |
| Autism spectrum conditions in myotonic dystrophy type 1: A study on 57 individuals with congenital and childhood forms | 10.1002/ajmg.b.30698 | Ekström, A.-B., Hakenäs-Plate, L., Samuelsson, L., Tulinius, M. and Wentz, E. (2008). Autism spectrum conditons in myotonic dystrophy type 1: A study on 57 individuals with congenital and childhood forms. *American Journal of Medical Genetics, Part B: Neuropsychiatric Genetics*, *147*(6), 918-926. https://doi.org/10.1002/ajmg.b.30698 | Wrong publication type |
| A longitudinal physical profile assessment of skeletal muscle manifestations in myotonic dystrophy | 10.1191/026921599674297570 | Nitz, J.C., Burns, Y.R. and Jackson, R.V. (1999). A longitudinal physical profile assessment of skeletal muscle manifestations in myotonic dystrophy. *Clinical Rehabilitation*, *13*(1), 64-73. https://doi.org/10.1191/026921599674297570 | No cognitive |
| Myotonic dystrophy: The burden for patients and their partners | 10.2340/16501977-0598 | Timman, R., Tibben, A. and Wintzen, A.R. (2010). Myotonic dystrophy: the burden for patients and their partners. *Journal of Rehabilitation Medicine*, *42*(9), 823-830. https://doi.org/10.2340/16501977-0598 | No cognitive |
| Fatigue and daytime sleepiness rating scales in myotonic dystrophy: A study of reliability | 10.1136/jnnp.2004.043455 | Laberge, L., Gagnon, C., Jean, S. and Mathieu, J. (2005). Fatigue and daytime sleepiness rating scales in myotonic dystrophy: A study of reliability. *Journal of Neurology, Neurosurgery and Psychiatry*, *76*(10), 1403-1405. https://doi.org/10.1136/jnnp.2004.043455 | No cognitive, Wrong study design |
| Domiciliary-assisted ventilation in patients with myotonic dystrophy | 10.1378/chest.121.2.459 | Nugent, A.-M., Smith, I.E. and Shneerson, J.M. (2002). Domiciliary-assisted ventilation in patients with myotonic dystrophy. *Chest*, *121*(2), 459-464. https://doi.org/10.1378/chest.121.2.459 | No cognitive, Wrong publication type |
| Myotonic dystrophy type 1 | 10.1002/9780470893159.ch36 | de Die-Smulders, C.E.M., Jennekens, F.G.I. and Faber, C.G. (2010). Myotonic dystrophy type 1. *Management of genetic syndromes: third edition*, 529-546. https://doi.org/10.1002/9780470893159.ch36 | Wrong publication type |
| Correlations between individual clinical manifestations and CTG repeat amplification in myotonic dystrophy | 10.1034/j.1399-0004.2000.570112.x | Marchini, C., Lonigro, R., Verriello, L., Pellizzari, L., Bergonzi, P. and Damante, G. (2000). Correlations between individual clinical manifestations and CTG repeat amplification in myotonic dystrophy. *Clinical Genetics*, *57*(1), 74-82. https://doi.org/10.1034/j.1399-0004.2000.570112.x | No cognitive |
| Cerebral involvement in myotonic dystrophies | 10.1002/mus.20800 | Meola, G. and Sansone, V. (2007). Cerebral involvement in myotonic dystrophies. *Muscle and Nerve*, *36*(3), 294-306. https://doi.org/10.1002/mus.20800 | Wrong publication type |
| Normal pressure hydrocephalus in a patient with myotonic dystrophy: Case report with a 10-year follow-up | 10.1227/01.NEU.0000204879.83077.C9 | Delavallée, M. and Raftopoulos, C. (2006). Normal pressure hydrocephalus in a patient with myotonic dystrophy: Case report with a 10-year follow-up. *Neurosurgery*, *58*(4), E796.1-E796.4. https://doi.org/10.1227/01.NEU.0000204879.83077.C9 | Wrong publication type |
| Myotonic dystrophy type 1 or Steinert's disease | 10.1007/978-1-4614-0653-2_18 | Romeo, V. (2012). Myotonic dystrophy type 1 or Steinert's disease. *Advances in Experimental Medicine and Biology*, *724*, 239-257. https://doi.org/10.1007/978-1-4614-0653-2_18 | No cognitive, Wrong publication type |
| Congenital myotonic dystrophy: assisted ventilation duration and outcome | 10.1542/peds.113.4.811 | Campbell, C., Sherlock, R., Jacob, P. and Blayney, M. (2004). Congenital myotonic dystrophy: assisted ventilation duration and outcome. *Pediatrics*, *113*(4), 811-816. https://doi.org/10.1542/peds.113.4.811 | No cognitive, Wrong publication type |
| Comparative analysis of brain structure, metabolism, and cognition in myotonic dystrophy 1 and 2 | 10.1212/WNL.0b013e3181d8c35f | Weber, Y.G., Roebling, R., Kassubek, J., Hoffmann, S., Rosenbohm, A., Wolf, M., Steinbach, P., Jurkat-Rott, K., Walter, H., Reske, S.N., Lehmann-Horn, F., Mottaghy, F.M. and Lerche, H. (2010). Comparative analysis of brain structure, metabolism, and cognition in myotonic dystrophy 1 and 2. *Neurology*, *74*(14), 1108-1117. https://doi.org/10.1212/WNL.0b013e3181d8c35f | No cognitive, Wrong publication type |
| Cognition and adaptive skills in myotonic dystrophy type 1: A study of 55 individuals with congenital and childhood forms | 10.1111/j.1469-8749.2009.03300.x | Ekström, A.-B., Hakenäs-Plate, L., Tulinius, M. and Wentz, E. (2009). Cognition and adaptive skills in myotonic dystrophy type 1: A study of 55 individuals with congenital and childhood forms. *Developmental Medicine and Child Neurology*, *51*(12), 982-990. https://doi.org/10.1111/j.1469-8749.2009.03300.x | Wrong study design |
| Myotonic dystrophy: Correlation of clinical symptoms with the size of the CTG trinucleotide repeat | 10.1007/BF00887824 | Jaspert, A., Fahsold, R., Grehl, H. and Claus, D. (1995). Myotonic dystrophy: correlation of clinical symptoms with the size of the CTG trinucleotide repeat. *Journal of Neurology*, *242*(2), 99-104. https://doi.org/10.1007/BF00887824 | No cognitive |
| Brain magnetic resonance images and molecular genetic analysis in myotonic dystrophy | Not found | Kuo, H.-C., Huang, C.-C., Chu, C.-C., Wei, Y.-Y., Chiang, S.-Y. and Hsiao, K.-M. (2002). Brain magnetic resonance images and molecular genetic analysis in myotonic dystrophy. *Acta Neurologica Taiwanica*, *11*(4), 187-193. | No cognitive |
| Brain disease and molecular analysis in myotonic dystrophy | 10.1097/00001756-199412000-00036 | Damian, M.S., Bachmann, G., Koch, M.C., Schilling, G., StÖppler, S. and Dorndorf, W. (1994). Brain disease and molecular analysis in myotonic dystrophy. *Neuroreport*, *5*(18), 2549-2552. https://doi.org/10.1097/00001756-199412000-00036 | Wrong study design |
| Auditory abnormalities, including 'precocious presbyacusis', in myotonic dystrophy: Original paper | 10.3109/00206099409071869 | Huygen, P.L.M., Verhagen, W.I.M. and Noten, J.F.P. (1994). Auditory abnormalities, including 'precocious presbyacusis', in myotonic dystrophy: original paper. *International Journal of Audiology*, *33*(2), 73-84. https://doi.org/10.3109/00206099409071869 | No cognitive |
| Cognition and event-related potentials in adult-onset non-demented myotonic dystrophy type 1 | 10.1016/j.clinph.2011.06.012 | Tanaka, H., Arai, M., Harada, M., Hozumi, A. and Hirata, K. (2012). Cognition and event-related potentials in adult-onset non-demented myotonic dystrophy type 1. *Clinical Neurophysiology*, *123*(2), 261-269. https://doi.org/10.1016/j.clinph.2011.06.012 | Wrong study design |
| Retrospective study on pet-spect imaging in a large cohort of myotonic dystrophy type 1 patients | 10.1007/s10072-010-0406-2 | Romeo, V., Pegoraro, E., Squarzanti, F., Sorarù, G., Ferrati, C., Ermani, M., Zucchetta, P., Chierichetti, F. and Angelini, C. (2010). Retrospective study on pet-spect imaging in a large cohort of myotonic dystrophy type 1 patients. *Neurological Sciences*, *31*(6), 757-763. https://doi.org/10.1007/s10072-010-0406-2 | No cognitive |
| Steinert disease | 10.1016/j.lpm.2007.01.002 | Bouhour, F., Bost, M. and Vial, C. (2007). Steinert disease. *Presse Medicale*, *36*(6), 965-971. https://doi.org/10.1016/j.lpm.2007.01.002 | No cognitive, Wrong publication type |
| Oral health status in a group of children and adolescents with myotonic dystrophy type 1 over a 4-year period | 10.1111/j.1365-263X.2009.01012.x | Engvall, M., SjÖgreen, L., Kjellberg, H., Robertson, A., Sundell, S. and Kiliaridis, S. (2009). Oral health status in a group of children and adolescents with myotonic dystrophy type 1 over a 4-year period. *International Journal of Paediatric Dentistry*, *19*(6), 412-422. https://doi.org/10.1111/j.1365-263X.2009.01012.x | No cognitive |
| Grey and white matter loss along cerebral midline structures in myotonic dystrophy type 2 | 10.1007/s00415-008-0997-1 | Minnerop, M., Luders, E., Specht, K., Ruhlmann, J., Schneider-Gold, C., Schröder, R., Thompson, P.M., Toga, A.W., Klockgether, T. and Kornblum, C. (2008). Grey and white matter loss along cerebral midline structures in myotonic dystrophy type 2. *Journal of Neurology*, *255*(12), 1904-1909. https://doi.org/10.1007/s00415-008-0997-1 | No DM1 |
| Magnetic resonance imaging and clinical correlates of intellectual impairment in myotonic dystrophy | 10.1001/archneur.1989.00520410070026 | Huber, S.J., Kissel, J.T., Shuttleworth, E.C., Clapp, L.E., Chakeres, D.W. and Brogan, M.A. (1989). Magnetic resonance imaging and clinical correlates of intellectual impairment in myotonic dystrophy. *Archives of Neurology*, *46*(5), 536-540. https://doi.org/10.1001/archneur.1989.00520410070026 | Wrong study design |
| Visual function in congenital and childhood myotonic dystrophy type 1 | 10.1016/j.ophtha.2010.01.055 | Ekström, A.-B., Tulinius, M., Sjöström, A. and Aring, E. (2010). Visual function in congenital and childhood myotonic dystrophy type 1. *Ophthalmology*, *117*(5), 976-982. https://doi.org/10.1016/j.ophtha.2010.01.055 | No cognitive |
| Brain proton magnetic resonance spectroscopy and brain atrophy in myotonic dystrophy | 10.1001/archneur.56.3.325 | Akiguchi, I., Nakano, S., Shiino, A., Kimura, R., Inubushi, T., Handa, J., Nakamura, M., Tanaka, M., Oka, N. and Kimura, J. (1999). Brain proton magnetic resonance spectroscopy and brain atrophy in myotonic dystrophy. *Archives of Neurology*, *56*(3), 325-330. https://doi.org/10.1001/archneur.56.3.325 | No cognitive |
| Involvement of the central nervous system in myotonic dystrophy | 10.1016/0022-510X(94)90071-X | Abe, K., Fujimura, H., Toyooka, K., Yorifuji, S., Nishikawa, Y., Hazama, T. and Yanagihara, T. (1994). Involvement of the central nervous system in myotonic dystrophy. *Journal of the Neurological Sciences*, *127*(2), 179-185. https://doi.org/10.1016/0022-510X(94)90071-X | Wrong study design |
| Living with myotonic dystrophy; what can be learned from couples? A qualitative study | 10.1186/1471-2377-11-86 | Cup, E.H.C., Kinébanian, A., Satink, T., Pieterse, A.J., Hendricks, H.T., Oostendorp, R.A.B., van der Wilt, G.J. and van Engelen, B.G.M. (2011). Living with myotonic dystrophy; what can be learned from couples? a qualitative study. *BMC Neurology*, 11. https://doi.org/10.1186/1471-2377-11-86 | No cognitive |
| Cognitive/personality pattern and triplet expansion size in adult myotonic dystrophy type 1 (DM1): CTG repeats, cognition and personality in DM1 | 10.1017/S0033291709990602 | Sistiaga, A., Urreta, I., Jodar, M., Cobo, A.M., Emparanza, J., Otaegui, D., Poza, J.J., Merino, J.J., Imaz, H., Martí-Mass, J.F. and López De Munain, A.(2010). Cognitive/personality pattern and triplet expansion size in adult myotonic dystrophy type 1 (DM1): CTG repeats, cognition and personality in DM1. *Psychological Medicine*, *40*(3), 487-495. https://doi.org/10.1017/S0033291709990602 | Wrong study design |
| Impact of biopsychosocial factors on chronic pain in persons with myotonic and facioscapulohumeral muscular dystrophy | 10.1177/1049909109335146 | Miró, J., Raichle, K.A., Carter, G.T., O'Brien, S.A., Abresch, R.T., McDonald, C.M. and Jensen, M.P.(2009). Impact of biopsychosocial factors on chronic pain in persons with myotonic and facioscapulohumeral muscular dystrophy. *American Journal of Hospice and Palliative Medicine*, *26*(4), 308-319. https://doi.org/10.1177/1049909109335146 | No cognitive |
| Is silent hypoglycemia part of ideal glycemic control in DM1 patients? - hypoglycemic state by CGMs vs. glycemic average | 10.1590/s0004-27302008000600010 | Maia, F.F.R. and Araújo, L.R. (2008). Is silent hypoglycemia part of ideal glycemic control in DM1 patients? - hypoglycemic state by CGMs vs. glycemic average. *Arquivos Brasileiros de Endocrinologia e Metabologia*, *52*(6), 994-1000. https://doi.org/10.1590/s0004-27302008000600010 | No cognitive, Wrong study design |
| Myotonic dystrophy: A new perspective on the treatment of a multisystemic disease | 10.1358/dof.2010.035.03.1416993 | Marra, C., Quaranta, D., Silvestri, G. and Modoni, A. (2010). Myotonic dystrophy: a new perspective on the treatment of a multisystemic disease. *Drugs of the Future*, *35*(3), 237-244. https://doi.org/10.1358/dof.2010.035.03.1416993 | No cognitive, Wrong publication type |
| Fatigue, excessive daytime sleepiness, and sleep-related breathing disorders in patients with myotonic dystrophies | 10.1007/s11818-011-0509-x | Boentert, M. and Young, P. (2011). Fatigue, excessive daytime sleepiness, and sleep-related breathing disorders in patients with myotonic dystrophies. *Somnologie*, *15*(1), 47-52. https://doi.org/10.1007/s11818-011-0509-x | No cognitive |
| Masticatory dysfunction in patients with myotonic dystrophy (type 1): A 5-year follow-up | 10.1111/j.1754-4505.2009.00093.x | Umemoto, G., Nakamura, H., Oya, Y. and Kikuta, T. (2009). Masticatory dysfunction in patients with myotonic dystrophy (type 1): A 5-year follow-up. *Special Care in Dentistry*, *29*(5), 210-214. https://doi.org/10.1111/j.1754-4505.2009.00093.x | No cognitive |
| Cortical damage in brains of patients with adult-form of myotonic dystrophy type 1 and no or minimal mri abnormalities | 10.1007/s00415-006-0248-2 | Giorgio, A., Dotti, M.T., Battaglini, M., Marino, S., Mortilla, M., Stromillo, M.L., Bramanti, P., Orrico, A., Federico, A. and De Stefano, N. (2006). Cortical damage in brains of patients with adult-form of myotonic dystrophy type 1 and no or minimal mri abnormalities. *Journal of Neurology*, *253*(11), 1471-1477. https://doi.org/10.1007/s00415-006-0248-2 | No cognitive |
| The myotonic dystrophies | 10.1055/s-2008-1040823 | Thornton, C.(1999). The myotonic dystrophies. _Seminars in neurology_, 19(1), 25-33. https://doi.org/10.1055/s-2008-1040823 | No cognitive |
| Myotonic dystrophy: Muscle involvement in relation to disease type and size of expanded CTG-repeat sequence | 10.1017/S0012162205000927 | Kroksmark, A.-K., Ekström, A.-B., Björck, E. and Tulinius, M. (2005). Myotonic dystrophy: muscle involvement in relation to disease type and size of expanded CTG-repeat sequence. *Developmental Medicine and Child Neurology*, *47*(7), 478-485. https://doi.org/10.1017/S0012162205000927 | No cognitive |
| P300 and respiratory findings in myotonic muscular dystrophy | Not found | Oliveri, M., Fierro, B., Lo Presti, R., Brighina, F., La Bua, V. and Caimi, G. (1999). P300 and respiratory findings in myotonic muscular dystrophy. *Functional neurology*, *14*(3), 149-154. | No cognitive |
| Open-label trial of recombinant human insulin-like growth factor 1/recombinant human insulin-like growth factor binding protein 3 in myotonic dystrophy type 1 | 10.1001/archneurol.2010.227 | Heatwole, C.R., Eichinger, K.J., Friedman, D.I., Hilbert, J.E., Jackson, C.E., Logigian, E.L., Martens, W.B., McDermott, M.P., Pandya, S.K., Quinn, C., Smirnow, A.M., Thornton, C.A. and Moxley III, R.T. (2011). Open-label trial of recombinant human insulin-like growth factor 1/recombinant human insulin-like growth factor binding protein 3 in myotonic dystrophy type 1. *Archives of neurology*, *68*(1), 37-44. https://doi.org/10.1001/archneurol.2010.227 | No cognitive |
| Type I diabetes mellitus in Chilean children under 5 years-old: Onset characteristics in contrast with other age groups | Not found | Eyzaguirre Croxatto, F., Peláez D., J.M., Sepúlveda R., C., Gaete V., X., Codner D., E., Unanue M., N., Ávila A., A., Ugarte P., F., Silva A., R. and García B., H. (2006). Type I diabetes mellitus in Chilean children under 5 years-old: onset characteristics in contrast with other age groups. *Revista Chilena de Pediatria*, *77*(4), 375-381. | No DM1 |
| Development of orofacial functions in young individuals with myotonic dystrophy: a retrospective study | Not found | Sjögreen, L., Engvall, M., Kiliaridis, S., Tulinius, M. and Lohmander, A. (2008). Development of orofacial functions in young individuals with myotonic dystrophy: A retrospective study. *Journal of Medical Speech-Language Pathology*, *16*(1), 33-41. | No cognitive |
| Developmental changes in expression of myotonic dystrophy protein kinase in the rat central nervous system | 10.1002/(SICI)1096-9861(19980511)394:3<309::AID-CNE3>3.0.CO;2-5 | Balasubramanyam, A., Iyer, D., Stringer, J.L., Beaulieu, C., Potvin, A., Neumeyer, A.M., Avruch, J. and Epstein, H.F. (1998). Developmental changes in expression of myotonic dystrophy protein kinase in the rat central nervous system. *Journal of Comparative Neurology*, *394*(3), 309-325. https://doi.org/10.1002/(SICI)1096-9861(19980511)394:3<309::AID-CNE3>3.0.CO;2-5 | No cognitive |
| Fatigue and daytime sleepiness in patients with myotonic dystrophy type 1: To lump or split? | 10.1016/j.nmd.2009.03.007 | Laberge, L., Dauvilliers, Y., Bégin, P., Richer, L., Jean, S. and Mathieu, J. (2009). Fatigue and daytime sleepiness in patients with myotonic dystrophy type 1: to lump or split?. *Neuromuscular Disorders*, *19*(6), 397-402. https://doi.org/10.1016/j.nmd.2009.03.007 | No cognitive, Wrong study design |
| Health supervision and anticipatory guidance in adult myotonic dystrophy type 1 | 10.1016/j.nmd.2010.08.006 | Gagnon, C., Chouinard, M.C., Laberge, L., Veillette, S., Bégin, P., Breton, R., Jean, S., Brisson, D., Gaudet, D. and Mathieu, J. (2010). Health supervision and anticipatory guidance in adult myotonic dystrophy type 1. *Neuromuscular Disorders*, *20*(12), 847-851. https://doi.org/10.1016/j.nmd.2010.08.006 | Wrong publication type |
| Conventional and quantitative electroencephalography in patients with Steinert's myotonic dystrophy | 10.33588/rn.3901.2004105 | Calderón-González, P.L., Parra-Rodríguez, M.A., Rodríguez-Martínez, W., Lestayo-O'Farrill, Z., Gutiérrez-Gil, J. and Montejo-Pujadas, Y. (2004). Conventional and quantitative electroencephalography in patients with steinert's myotonic dystrophy. *Revista de Neurologia*, *39*(1), 12-17. https://doi.org/10.33588/rn.3901.2004105 | No cognitive |
| Psychiatric and cognitive phenotype in children and adolescents with myotonic dystrophy | 10.1007/s00787-009-0037-4 | Douniol, M., Jacquette, A., Guilé, J.-M., Tanguy, M.-L., Angeard, N., Héron, D., Plaza, M. and Cohen, D. (2009). Psychiatric and cognitive phenotype in children and adolescents with myotonic dystrophy. *European Child and Adolescent Psychiatry*, *18*(12), 705-715. https://doi.org/10.1007/s00787-009-0037-4 | Wrong publication type |
| Cardiovascular autonomic control in myotonic dystrophy type 1: A correlative study with clinical and genetic data | 10.1016/j.nmd.2003.11.002 | Di Leo, R., Rodolico, C., De Gregorio, C., Recupero, A., Coglitore, S., Annesi, G., Toscano, A., Messina, C. and Vita, G. (2004). Cardiovascular autonomic control in myotonic dystrophy type 1: a correlative study with clinical and genetic data. *Neuromuscular Disorders*, *14*(2), 136-141. https://doi.org/10.1016/j.nmd.2003.11.002 | No cognitive, Wrong study design |
| CIS effects of CTG expansion in myotonic dystrophy type 1 | 10.1016/B978-012369462-1/50005-3 | Reddy, S. and Paul, S. (2006). CIS effects of CTG expansion in myotonic dystrophy type 1. *Genetic Instabilities and Neurological Diseases*, 55-78. https://doi.org/10.1016/B978-012369462-1/50005-3 | No cognitive |
| Anaesthetic management of the child with myotonic dystrophy: Epidural anaesthesia as an alternative to general anaesthesia | 10.1111/j.1460-9592.1995.tb00319.x | TOBIAS, J.D.(1995). Anaesthetic management of the child with myotonic dystrophy: epidural anaesthesia as an alternative to general anaesthesia. *Pediatric Anesthesia*, *5*(5), 335-338. https://doi.org/10.1111/j.1460-9592.1995.tb00319.x | No cognitive |
| Myotonic dystrophies as a brain disorder | 10.1007/s10072-010-0414-2 | Meola, G.(2010). Myotonic dystrophies as a brain disorder. _Neurological sciences_, 31(6), 863-864. https://doi.org/10.1007/s10072-010-0414-2 | Wrong publication type |
| Quantification of brain atrophy in patients with myotonic dystrophy and proximal myotonic myopathy: A controlled 3-dimensional magnetic resonance imaging study | 10.1016/S0304-3940(03)00740-7 | Kassubek, J., Juengling, F.D., Hoffmann, S., Rosenbohm, A., Kurt, A., Jurkat-Rott, K., Steinbach, P., Wolf, M., Ludolph, A.C., Lehmann-Horn, F., Lerche, H. and Weber, Y.G. (2003). Quantification of brain atrophy in patients with myotonic dystrophy and proximal myotonic myopathy: a controlled 3-dimensional magnetic resonance imaging study. *Neuroscience Letters*, *348*(2), 73-76. https://doi.org/10.1016/S0304-3940(03)00740-7 | No cognitive |
| Changes in pain-related beliefs, coping, and catastrophizing predict changes in pain intensity, pain interference, and psychological functioning in individuals with myotonic muscular dystrophy and facioscapulohumeral dystrophy | 10.1097/AJP.0b013e31822019b1 | Nieto, R., Raichle, K.A., Jensen, M.P. and Miró, J. (2012). Changes in pain-related beliefs, coping, and catastrophizing predict changes in pain intensity, pain interference, and psychological functioning in individuals with myotonic muscular dystrophy and facioscapulohumeral dystrophy. *Clinical Journal of Pain*, *28*(1), 47-54. https://doi.org/10.1097/AJP.0b013e31822019b1 | No cognitive |
| Rasch-built myotonic dystrophy type 1 activity and participation scale (DM1-activ) | 10.1016/j.nmd.2010.03.010 | Hermans, M.C.E., Faber, C.G., De Baets, M.H., de Die-Smulders, C.E.M. and Merkies, I.S.J. (2010). Rasch-built myotonic dystrophy type 1 activity and participation scale (DM1-activ). *Neuromuscular Disorders*, *20*(5), 310-318. https://doi.org/10.1016/j.nmd.2010.03.010 | No cognitive |
| Hydrocephalus and cognitive decline in myotonic dystrophy: Case report and literature review | 10.1016/S0003-9993(98)90105-8 | Moroz, A. and Kalva, S. (1998). Hydrocephalus and cognitive decline in myotonic dystrophy: case report and literature review. *Archives of physical medicine and rehabilitation*, *79*(8), 1022-1023. https://doi.org/10.1016/S0003-9993(98)90105-8 | Wrong publication type |
| Health-related quality of life in patients with myotonic dystrophy type 1 and amyotrophic lateral sclerosis | Not found | Peric, S., Rakocevic-Stojanovic, V., Stevic, Z., Basta, I., Pavlovic, S., Vujanac, V., Marjanovic, I. and Lavrnic, D. (2010). Health-related quality of life in patients with myotonic dystrophy type 1 and amyotrophic lateral sclerosis. *Acta neurologica belgica*, *110*(1), 71-77. | No cognitive, Wrong study design |
| Toward understanding cognitive impairment in patients with myotonic dystrophy type 1 | 10.1093/arclin/acq016 | Zalonis, I., Bonakis, A., Christidi, F., Vagiakis, E., Papageorgiou, S.G., Kalfakis, N., Manta, P. and Vassilopoulos, D. (2010). Toward understanding cognitive impairment in patients with myotonic dystrophy type 1. *Archives of clinical neuropsychology*, *25*(4), 303-313. https://doi.org/10.1093/arclin/acq016 | Wrong publication type |
| A study of the cognitive and psychological profile in 16 children with congenital or juvenile myotonic dystrophy | 10.1111/j.1399-0004.1997.tb02533.x | Steyaert, J., Umans, S., Willekens, D., Legius, E., Pijkels, E., De Die-Smulders, C., Van Den Berghe, H. and Fryns, J.-P. (1997). A study of the cognitive and psychological profile in 16 children with congenital or juvenile myotonic dystrophy. *Clinical Genetics*, *52*(3), 135-141. https://doi.org/10.1111/j.1399-0004.1997.tb02533.x | Wrong study design |
| Myotonic dystrophy types 1 and 2 | 10.1016/B978-0-08-045031-5.00015-3 | Ashizawa, T. and Sarkar, P.S. (2011). Myotonic dystrophy types 1 and 2. *Handbook of clinical neurology*, *101*, 193-237. https://doi.org/10.1016/B978-0-08-045031-5.00015-3 | Wrong publication type |
| Relative sparing of extraocular muscles in myotonic dystrophy: An electrooculographic study | 10.1111/j.1600-0404.1997.tb00088.x | Di Costanzo, A., Toriello, A., Mottola, A., Di Iorio, G., Bonavita, V. and Tedeschi, G. (1997). Relative sparing of extraocular muscles in myotonic dystrophy: An electrooculographic study. *Acta Neurologica Scandinavica*, *95*(3), 158-163. https://doi.org/10.1111/j.1600-0404.1997.tb00088.x | No cognitive |
| Posture and gait abilities in patients with myotonic dystrophy (Steinert disease). Evaluation on the short-term of a rehabilitation program | 10.1016/j.rehab.2010.06.004 | Missaoui, B., Rakotovao, E., Bendaya, S., Mane, M., Pichon, B., Faucher, M. and Thoumie, P. (2010). Posture and gait abilities in patients with myotonic dystrophy (steinert disease). evaluation on the short-term of a rehabilitation program. *Annals of Physical and Rehabilitation Medicine*, *53*(6), 387-398. https://doi.org/10.1016/j.rehab.2010.06.004 | No cognitive |
| Anesthesia and myotonic dystrophy type 2: A case series | 10.1007/s12630-009-9244-1 | Weingarten, T.N., Hofer, R.E., Milone, M. and Sprung, J. (2010). Anesthesia and myotonic dystrophy type 2: a case series. *Canadian Journal of Anesthesia*, *57*(3), 248-255. https://doi.org/10.1007/s12630-009-9244-1 | No DM1, No cognitive |
| Cognitive impairment and (CTG)n expansion in myotonic dystrophy patients | 10.1016/S0006-3223(99)00016-5 | Perini, G.I., Menegazzo, E., Ermani, M., Zara, M., Gemma, A., Ferruzza, E., Gennarelli, M. and Angelini, C. (1999). Cognitive impairment and (CTG)n expansion in myotonic dystrophy patients. *Biological Psychiatry*, *46*(3), 425-431. https://doi.org/10.1016/S0006-3223(99)00016-5 | Wrong study design |
| The kohs' blocks test as an important instrument to investigate the visuo-spatial impairments in myotonic dystrophy. part i. quantitative and qualitative analysis | 10.1590/S0004-282X1999000400002 | Wigg, C.M.D. and Duro, L.A.A. (1999). The kohs' blocks test as an important instrument to investigate the visuo-spatial impairments in myotonic dystrophy. part i. quantitative and qualitative analysis. *Arquivos de Neuro-Psiquiatria*, *57*(3), 547-555. https://doi.org/10.1590/S0004-282X1999000400002 | No cognitive |
| Patient registries and trial readiness in myotonic dystrophy - treat-nmd/marigold international workshop report | 10.1016/j.nmd.2009.08.009 | Thompson, R., Schoser, B., Monckton, D.G., Blonsky, K. and Lochmüller, H. (2009). Patient registries and trial readiness in myotonic dystrophy - treat-nmd/marigold international workshop report. *Neuromuscular Disorders*, *19*(12), 860-866. https://doi.org/10.1016/j.nmd.2009.08.009 | No cognitive |
| Myotonic dystrophy curschmann-steinert | 10.1055/s-2006-927292 | Papageorgiou, E., Bock, S.W. and Schiefer, U. (2007). Myotonic dystrophy curschmann-steinert. *Klinische Monatsblatter fur Augenheilkunde*, *224*(1), 70-75. https://doi.org/10.1055/s-2006-927292 | Wrong publication type |
| Cognitive deficits and CTG repeat expansion size in classical myotonic dystrophy type 1 (DM1) | 10.1186/1744-9081-2-16 | Winblad, S., Lindberg, C. and Hansen, S. (2006). Cognitive deficits and CTG repeat expansion size in classical myotonic dystrophy type 1 (DM1). *Behavioral and Brain Functions*, *2*. https://doi.org/10.1186/1744-9081-2-16 | Wrong study design |
| Mis-splicing of tau exon 10 in myotonic dystrophy type 1 is reproduced by overexpression of celf2 but not by mbnl1 silencing | 10.1016/j.bbadis.2011.03.010 | Dhaenens, C.M., Tran, H., Frandemiche, M.-L., Carpentier, C., Schraen-Maschke, S., Sistiaga, A., Goicoechea, M., Eddarkaoui, S., Van Brussels, E., Obriot, H., Labudeck, A., Gevaert, M.H., Fernandez-Gomez, F., Charlet-Berguerand, N., Deramecourt, V., Maurage, C.A., Buée, L., de Munain, A.L., Sablonnière, B., Caillet-Boudin, M.L. and Sergeant, N. (2011). Mis-splicing of tau exon 10 in myotonic dystrophy type 1 is reproduced by overexpression of celf2 but not by mbnl1 silencing. *Biochimica et Biophysica Acta - Molecular Basis of Disease*, *1812*(7), 732-742. https://doi.org/10.1016/j.bbadis.2011.03.010 | No cognitive |
| White matter abnormalities and neurocognitive correlates in children and adolescents with myotonic dystrophy type 1: a diffusion tensor imaging study | 10.1016/j.nmd.2010.11.013 | Wozniak, J.R., Mueller, B.A., Ward, E.E., Lim, K.O. and Day, J.W. (2011). White matter abnormalities and neurocognitive correlates in children and adolescents with myotonic dystrophy type 1: a diffusion tensor imaging study. *Neuromuscular Disorders*, *21*(2), 89-96. https://doi.org/10.1016/j.nmd.2010.11.013 | Wrong study design |
| Neuropathological changes of the brain in myotonic dystrophy - some new observations | 10.1016/0022-510X(87)90105-5 | Ono, S., Inoue, K., Mannen, T., Kanda, F., Jinnai, K. and Takahashi, K. (1987). Neuropathological changes of the brain in myotonic dystrophy - some new observations. *Journal of the Neurological Sciences*, *81*(2), 301-320. https://doi.org/10.1016/0022-510X(87)90105-5 | No cognitive |
| Presentation, clinical course, and outcome of the congenital form of myotonic dystrophy | 10.1016/0887-8994(94)90104-X | Roig, M., Balliu, P.-R., Navarro, C., Brugera, R. and Losada, M. (1994). Presentation, clinical course, and outcome of the congenital form of myotonic dystrophy. *Pediatric Neurology*, *11*(3), 208-213. https://doi.org/10.1016/0887-8994(94)90104-X | No cognitive, Wrong study design |
| A neurodevelopmental approach to myotonic dystrophy type 1. | 10.1111/dmcn.14194 | Angeard, N. (2019). A neurodevelopmental approach to myotonic dystrophy type 1. *Developmental Medicine and Child Neurology*, *61*(10), 1126. https://doi.org/10.1111/dmcn.14194 | No cognitive |
| Neuropsychological assessment should always be considered in myotonic dystrophy type 2. | 10.1097/WNN.0000000000000263 | Theodosiou, T., Christidi, F., Xirou, S., Bede, P., Karavasilis, E., Papadopoulos, C., Kourtesis, P., Pantoleon, V., Kararizou, E., Papadimas, G., and Zalonis, I. (2021). Neuropsychological assessment should always be considered in myotonic dystrophy type 2. *Cognitive and Behavioral Neurology*, *34*(1), 1-10. https://doi.org/10.1097/WNN.0000000000000263 | No DM1 |
| Age-related cognitive decline in adults with DM1: A 9-year longitudinal study. | 10.1051/medsci/201733s114 | Michon, C.C. and Réveillère, C. (2017). Age-related cognitive decline in adults with DM1: A 9-year longitudinal study. *Medecine Aciences : m/s*, 33, 61-62. https://doi.org/10.1051/medsci/201733s114 | Wrong publication type |
| Brain imaging in myotonic dystrophy type 1: A systematic review. | 10.1212/WNL.0000000000004300 | Okkersen, K., Monckton, D.G., Le, N., Tuladhar, A.M., Raaphorst, J., and van Engelen, B.G.M. (2017). Brain imaging in myotonic dystrophy type 1: a systematic review.. *Neurology*, *89*(9), 960-969. https://doi.org/10.1212/WNL.0000000000004300 | No cognitive, Wrong publication type |
| Routine clinical diagnosis of cognitive impairments. | 10.1055/s-0033-1336945 | Czekaj, A., Steinert, T., and Uhlmann, C. (2014). Routine clinical diagnosis of cognitive impairments. *Psychiatrische Praxis*, *41*(3), 166-7. https://doi.org/10.1055/s-0033-1336945 | foreign language, No tracking possible |
| Comprehensive four-year disease progression assessment of myotonic dystrophy type 1. | 10.1016/j.nmd.2024.08.005 | la Fontaine, L.A., Bruijnes, J.E., Smulders, F.H., Gorissen-Brouwers, C., Karnebeek, I.E., Braakman, H.M., Klinkenberg, S., Mul, K., 't Hoen, P.A., van Kuijk, S.M., van Engelen, B.G., Merkies, I.S. and Faber, C.G. (2024). Comprehensive four-year disease progression assessment of myotonic dystrophy type 1. *Neuromuscular Disorders*, *43*, 44-52. https://doi.org/10.1016/j.nmd.2024.08.005 | No cognitive |
| Neurocognitive disorder in myotonic dystrophy type 1. | 10.1016/j.heliyon.2024.e30875 | Winblad S, Eliasdottir O, Nordström S, Lindberg C(2024). Neurocognitive disorder in myotonic dystrophy type 1. *Heliyon*, *10*(10), e30875. https://doi.org/10.1016/j.heliyon.2024.e30875 | Wrong study design |
| Clinical features and genetic spectrum of a multicenter chinese cohort with myotonic dystrophy type 1. | 10.1186/s13023-024-03114-z | Zhong H, Zeng L, Yu X, Ke Q, Dong J, Chen Y, Luo L, Chang X, Guo J, Wang Y, Xiong H, Liu R, Liu C, Wu J, Lin J, Xi J, Zhu W, Tan S, Liu F, Lu J, Zhao C and Luo S(2024). Clinical features and genetic spectrum of a multicenter chinese cohort with myotonic dystrophy type 1. *Orphanet Journal of Rare Diseases*, *19*(1), 103. https://doi.org/10.1186/s13023-024-03114-z | No cognitive |
| Common characteristics between frailty and myotonic dystrophy type 1: A narrative review. | 10.14336/AD.2024.0950 | Garmendia J, Labayru G, Souto Barreto P, Vergara I, de Munain AL and Sistiaga A. (2024). Common characteristics between frailty and myotonic dystrophy type 1: a narrative review. *Aging and Disease*. https://doi.org/10.14336/AD.2024.0950 | Wrong publication type |
| Taking control! Structural and behavioural plasticity in response to game-based inhibition training in older adults. | 10.1016/j.neuroimage.2017.05.026 | Kühn S, Lorenz RC, Weichenberger M, Becker M, Haesner M, O'Sullivan J, Steinert A, Steinhagen-Thiessen E, Brandhorst S, Bremer T and Gallinat J (2017). Taking control! Structural and behavioural plasticity in response to game-based inhibition training in older adults. *Neuroimage*, *156*, 199-206. https://doi.org/10.1016/j.neuroimage.2017.05.026 | No DM1 |
| Myotonic dystrophy type 1: Clinical manifestations in children and adolescents. | 10.1136/archdischild-2018-314837 | Ho G, Carey KA, Cardamone M and Farrar MA (2019). Myotonic dystrophy type 1: Clinical manifestations in children and adolescents. *Archives of Disease in Childhood*, *104*(1), 48-52. https://doi.org/10.1136/archdischild-2018-314837 | Wrong study design |
| Increased functional connectivity of white-matter in myotonic dystrophy type 1. | 10.3389/fnins.2022.953742 | Li J, Li J, Huang P, Huang LN, Ding QG, Zhan L, Li M, Zhang J, Zhang H, Cheng L, Li H, Liu DQ, Zhou HY and Jia XZ (2022). Increased functional connectivity of white-matter in myotonic dystrophy type 1. *Frontiers in Neuroscience*, *16*, 953742. https://doi.org/10.3389/fnins.2022.953742 | No cognitive |
| Clinical outcome evaluations and cbt response prediction in myotonic dystrophy. | 10.3233/JND-210634 | van As D, Okkersen K, Bassez G, Schoser B, Lochmüller H, Glennon JC, Knoop H, van Engelen BGM and 't Hoen PAC (2021). Clinical outcome evaluations and CBT response prediction in myotonic dystrophy. *Journal of Neuromuscular Diseases*, *8*(6), 1031-1046. https://doi.org/10.3233/JND-210634 | No cognitive |
| Myotonic dystrophy type 1 (Steinert disease): 29 years of experience at a tertiary pediatric hospital. | 10.1016/j.ejpn.2023.12.001 | Cascais I, Garrido C, Morais L, Amorim R, Lima R, Mansilha HF, Correia T, Oliveira A and Santos M (2024). Myotonic dystrophy type 1 (Steinert disease): 29 years of experience at a tertiary pediatric hospital.. *European Journal of Paediatric Neurology*, *48*, 85-90. https://doi.org/10.1016/j.ejpn.2023.12.001 | No cognitive |
| Scapular dyskinesis in myotonic dystrophy type 1: Clinical characteristics and genetic investigations. | 10.1007/s00415-019-09494-8 | Voermans NC, van der Bilt RC, IJspeert J, Hogrel JY, Jeanpierre M, Behin A, Laforet P, Stojkovic T, van Engelen BG, Padberg GW, Sacconi S, Lemmers RJLF, van der Maarel SM, Eymard B and Bassez G (2019). Scapular dyskinesis in myotonic dystrophy type 1: clinical characteristics and genetic investigations. *Journal of Neurology*, *266*(12), 2987-2996. https://doi.org/10.1007/s00415-019-09494-8 | No cognitive |
| Neurodegeneration trajectory in pediatric and adult/late DM1: A follow-up mri study across a decade. | 10.1002/acn3.51163 | Labayru G, Jimenez-Marin A, Fernández E, Villanua J, Zulaica M, Cortes JM, Díez I, Sepulcre J, López de Munain A and Sistiaga A. (2020). Neurodegeneration trajectory in pediatric and adult/late DM1: A follow-up mri study across a decade. *Annals of Clinical and Translational Neurology*, *7*(10), 1802-1815. https://doi.org/10.1002/acn3.51163 | Wrong study design |
| An evaluation of 24 h holter monitoring in patients with myotonic dystrophy type 1. | 10.1093/europace/euac104 | Joosten IBT, Janssen CEW, Horlings CGC, den Uijl D, Evertz R, van Engelen BGM, Faber CG and Vernooy K. (2023). An evaluation of 24 h holter monitoring in patients with myotonic dystrophy type 1. *Europace: european pacing, arrhythmias, and cardiac electrophysiology: journal of the working groups on cardiac pacing, arrhythmias, and cardiac cellular electrophysiology of the european society of cardiology*, *25*(1), 156-163. https://doi.org/10.1093/europace/euac104 | No cognitive |
| Myotonic dystrophy and Huntington's disease care: "We like to think we're making a difference". | 10.1017/cjn.2016.257 | LaDonna KA, Watling CJ, Ray SL, Piechowicz C and Venance SL (2016). Myotonic dystrophy and Huntington's disease care: "We like to think we're making a difference". *The Canadian Journal of Neurological Sciences. Le Journal Canadien des Sciences Neurologiques*, *43*(5), 678-86. https://doi.org/10.1017/cjn.2016.257 | No cognitive |
| Risk factors for ischaemic stroke in myotonic dystrophy type 1. | 10.1556/650.2022.32620 | Varga D, Perecz B, Sípos A, Jedlicska D and Pál E (2022). Risk factors for ischaemic stroke in myotonic dystrophy type 1. *Orvosi Hetilap*, *163*(49), 1962-1966. https://doi.org/10.1556/650.2022.32620 | No cognitive, Wrong publication type |
| Multidisciplinary care of patients with myotonic dystrophy type 1 (DM1) in south aquitaine. | 10.1051/medsci/2021190 | Flabeau O and Bisson T (2021). Multidisciplinary care of patients with myotonic dystrophy type 1 (DM1) in south aquitaine. *Medecine sciences, 37*, 32-35. https://doi.org/10.1051/medsci/2021190 | No cognitive |
| Cognitive deficits, apathy, and hypersomnolence represent the core brain symptoms of adult-onset myotonic dystrophy type 1. | 10.3389/fneur.2021.700796 | Miller JN, Kruger A, Moser DJ, Gutmann L, van der Plas E, Koscik TR, Cumming SA, Monckton DG and Nopoulos PC (2021). Cognitive deficits, apathy, and hypersomnolence represent the core brain symptoms of adult-onset myotonic dystrophy type 1. *Frontiers in Neurology*, *12*, 700796. https://doi.org/10.3389/fneur.2021.700796 | Wrong study design |
| Longitudinal changes in white matter as measured with diffusion tensor imaging in adult-onset myotonic dystrophy type 1. | 10.1016/j.nmd.2023.05.010 | Koscik TR, van der Plas E, Long JD, Cross S, Gutmann L, Cumming SA, Monckton DG, Shields RK, Magnotta V and Nopoulos PC (2023). Longitudinal changes in white matter as measured with diffusion tensor imaging in adult-onset myotonic dystrophy type 1. *Neuromuscular Disorders*, *33*(8), 660-669. https://doi.org/10.1016/j.nmd.2023.05.010 | No cognitive |
| Tractography reveals diffuse white matter abnormalities in myotonic dystrophy type 1. | 10.1016/j.jns.2014.04.005 | Wozniak JR, Mueller BA, Lim KO, Hemmy LS and Day JW (2014). Tractography reveals diffuse white matter abnormalities in myotonic dystrophy type 1. *Journal of the Neurological Sciences*, *341*(1), 73-8. https://doi.org/10.1016/j.jns.2014.04.005 | Wrong study design |
| Elevated serum neurofilament light chain (NFL) as a potential biomarker of neurological involvement in myotonic dystrophy type 1 (DM1). | 10.1007/s00415-022-11165-0 | Nicoletti TF, Rossi S, Vita MG, Perna A, Guerrera G, Lino F, Iacovelli C, Di Natale D, Modoni A, Battistini L and Silvestri G (2022). Elevated serum neurofilament light chain (nfl) as a potential biomarker of neurological involvement in myotonic dystrophy type 1 (DM1). *Journal of Neurology*, *269*(9), 5085-5092. https://doi.org/10.1007/s00415-022-11165-0 | Wrong publication type |
| What do we know today about the prospective long-term course of social anxiety disorder? A systematic literature review. | 10.1016/j.janxdis.2013.08.002 | Steinert C, Hofmann M, Leichsenring F and Kruse J (2013). What do we know today about the prospective long-term course of social anxiety disorder? a systematic literature review. *Journal of Anxiety Disorders*, *27*(7), 692-702. https://doi.org/10.1016/j.janxdis.2013.08.002 | No DM1, No cognitive,Wrong publication type |
| Participation restriction in childhood phenotype of myotonic dystrophy type 1: A systematic retrospective chart review. | 10.1111/dmcn.13282 | Gagnon C, Kierkegaard M, Blackburn C, Chrestian N, Lavoie M, Bouchard MF and Mathieu J (2017). Participation restriction in childhood phenotype of myotonic dystrophy type 1: A systematic retrospective chart review.. *Developmental Medicine and Child Neurology*, *59*(3), 291-296. https://doi.org/10.1111/dmcn.13282 | Wrong publication type |
| Cognitive and neuroradiological findings in myotonic dystrophy. | Not found | Sinforiani E, Sandrini G, Martelli A, Mauri M, Uggetti C, Bono G and Nappi G (1991). Cognitive and neuroradiological findings in myotonic dystrophy. *Functional Neurology*, *6*(4), 377-84. | Wrong study design |
| Treatment of cardiovascular risk factors to prevent cognitive decline and dementia: A systematic review. | 10.2147/vhrm.s7343 | Ligthart SA, Moll van Charante EP, Van Gool WA and Richard E (2010). Treatment of cardiovascular risk factors to prevent cognitive decline and dementia: A systematic review. *Vascular Health and Risk Management*, *6*, 775-85. https://doi.org/10.2147/vhrm.s7343 | No DM1, Wrong publication type |
| Peer support intervention improves pain-related outcomes among rural adults with diabetes and chronic pain at 12-month follow-up. | 10.1111/jrh.12422 | Khodneva Y, Richman J, Andreae S, Cherrington A and Safford MM (2021). Peer support intervention improves pain-related outcomes among rural adults with diabetes and chronic pain at 12-month follow-up. *The Journal of Rural Health*, *37*(2), 394-405. https://doi.org/10.1111/jrh.12422 | No DM1 |
| Uncovering health and social care needs among myotonic dystrophy patients: utility of the needs and provisions complexity scale. | 10.1111/ane.13086 | Holmøy AKT, Johannessen CH, Hope S, van Walsem MR, Aanonsen NO and Hassel B (2019). Uncovering health and social care needs among myotonic dystrophy patients: utility of the needs and provisions complexity scale. *Acta Neurologica Scandinavica*, *139*(6), 526-532. https://doi.org/10.1111/ane.13086 | No cognitive |
| Quantitative muscle mri depicts increased muscle mass after a behavioral change in myotonic dystrophy type 1. | 10.1148/radiol.2020192518 | Heskamp L, Okkersen K, van Nimwegen M, Ploegmakers MJ, Bassez G, Deux JF, van Engelen BG and Heerschap A (2020). Quantitative muscle mri depicts increased muscle mass after a behavioral change in myotonic dystrophy type 1. *Radiology*, *297*(1), 132-142. https://doi.org/10.1148/radiol.2020192518 | No cognitive |
| The saccade velocity test. | 10.1212/wnl.25.11.1071 | Baloh RW, Konrad HR, Sills AW and Honrubia V (1975). The saccade velocity test. *Neurology*, *25*(11), 1071-6. https://doi.org/10.1212/wnl.25.11.1071 | No cognitive |
| Somatic cag repeat expansion in blood associates with biomarkers of neurodegeneration in Huntington's disease decades before clinical motor diagnosis. | 10.1038/s41591-024-03424-6 | Scahill RI, Farag M, Murphy MJ, Hobbs NZ, Leocadi M, Langley C, Knights H, Ciosi M, Fayer K, Nakajima M, Thackeray O, Gobom J, Rönnholm J, Weiner S, Hassan YR, Ponraj NKP, Estevez-Fraga C, Parker CS, Malone IB, Hyare H, Long JD, Heslegrave A, Sampaio C, Zhang H, Robbins TW, Zetterberg H, Wild EJ, Rees G, Rowe JB, Sahakian BJ, Monckton DG, Langbehn DR and Tabrizi SJ (2025). Somatic cag repeat expansion in blood associates with biomarkers of neurodegeneration in huntington's disease decades before clinical motor diagnosis.. *Nature Medicine*, *31*(3), 807-818. https://doi.org/10.1038/s41591-024-03424-6 | No DM1 |
| Psychodynamic therapy: as efficacious as other empirically supported treatments? A meta-analysis testing equivalence of outcomes. | 10.1176/appi.ajp.2017.17010057 | Steinert C., Munder T., Rabung S., Hoyer J., and Leichsenring, F. (2017). Psychodynamic therapy: As efficacious as other empirically supported treatments? A meta-analysis testing equivalence of outcomes. *The American Journal of Psychiatry*, *174*(10), 943-953. https://doi.org/10.1176/appi.ajp.2017.17010057 | No DM1 |
| Comparisons of intellectual capacities between mild and classic adult-onset phenotypes of myotonic dystrophy type 1 (DM1). | 10.1186/s13023-014-0186-5 | Jean S, Richer L, Laberge L and Mathieu J (2014). Comparisons of intellectual capacities between mild and classic adult-onset phenotypes of myotonic dystrophy type 1 (DM1). *Orphanet Journal of Rare Diseases*, *9*, *186*. https://doi.org/10.1186/s13023-014-0186-5 | Wrong study design |
| Experiences with bariatric surgery in patients with facioscapulohumeral dystrophy and myotonic dystrophy type 1: A qualitative study. | 10.1016/j.nmd.2018.09.003 | Abel EEDH, Cup EHC, Lanser A, Leclercq WKG, Raaphorst J, Padberg GW, Satink T and Voermans NC (2018). Experiences with bariatric surgery in patients with facioscapulohumeral dystrophy and myotonic dystrophy type 1: A qualitative study. *Neuromuscular Disorders*, *28*(11), 938-946. https://doi.org/10.1016/j.nmd.2018.09.003 | No cognitive |
| White matter lesions detected by magnetic resonance imaging in neonates and children with congenital myotonic dystrophy. | 10.1016/j.pediatrneurol.2019.03.004 | Peglar LM, Nagaraj UD, Tian C and Venkatesan C (2019). White matter lesions detected by magnetic resonance imaging in neonates and children with congenital myotonic dystrophy. *Pediatric neurology*, *96*, 64-69. https://doi.org/10.1016/j.pediatrneurol.2019.03.004 | No cognitive |
| Functional deterioration and selenium-vitamin e treatment in myotonic dystrophy. a placebo-controlled study. | 10.1111/j.1365-2796.1994.tb01061.x | Orndahl G, Grimby G, Grimby A, Johansson G and Wilhelmsen L (1994). Functional deterioration and selenium-vitamin e treatment in myotonic dystrophy. a placebo-controlled study. *Journal of Internal Medicine*, *235*(3), 205-10. https://doi.org/10.1111/j.1365-2796.1994.tb01061.x | No cognitive |
| Learning through teaching: How physicians learn medicine in authentic clinical contexts. | 10.1097/ACM.0000000000005662 | Frija-Gruman NM, Steinert Y, Macdonald ME and Sun NZ (2025). Learning through teaching: How physicians learn medicine in authentic clinical contexts.. *Academic Medicine: Journal of the Association of American Medical Colleges*, *100*(3), 306-312. https://doi.org/10.1097/ACM.0000000000005662 | No DM1 |
| Swallowing dysfunction in patients with nephropathic cystinosis. | 10.1016/j.ymgme.2019.01.011 | van Rijssel AE, Knuijt S, Veys K, Levtchenko EN and Janssen MCH (2019). Swallowing dysfunction in patients with nephropathic cystinosis. *Molecular Genetics and Metabolism*, *126*(4), 413-415. https://doi.org/10.1016/j.ymgme.2019.01.011 | No DM1 |
| Care for capabilities: implementing the capability approach in rehabilitation of patients with neuromuscular diseases. study protocol of the controlled before-after recap-nmd study. | 10.1371/journal.pone.0261475 | Bloemen B, Pijpers E, Cup E, Groothuis J, van Engelen B and van der Wilt GJ (2021). Care for capabilities: Implementing the capability approach in rehabilitation of patients with neuromuscular diseases. Study protocol of the controlled before-after recap-nmd study. *Plos ONE*, *16*(12), e0261475. https://doi.org/10.1371/journal.pone.0261475 | Wrong publication type |
| Diffusion tensor imaging reveals widespread white matter abnormalities in children and adolescents with myotonic dystrophy type 1. | 10.1007/s00415-012-6771-4 | Wozniak JR, Mueller BA, Bell CJ, Muetzel RL, Lim KO and Day JW (2013). Diffusion tensor imaging reveals widespread white matter abnormalities in children and adolescents with myotonic dystrophy type 1.. *Journal of Neurology*, *260*(4), 1122-31. https://doi.org/10.1007/s00415-012-6771-4 | No cognitive |
| Cognitive behaviour therapy plus aerobic exercise training to increase activity in patients with myotonic dystrophy type 1 (DM1) compared to usual care (optimistic): Study protocol for randomised controlled trial. | 10.1186/s13063-015-0737-7 | van Engelen B (2015). Cognitive behaviour therapy plus aerobic exercise training to increase activity in patients with myotonic dystrophy type 1 (DM1) compared to usual care (optimistic): study protocol for randomised controlled trial. *Trials*, *16*, 224. https://doi.org/10.1186/s13063-015-0737-7 | No cognitive, Wrong study design |
| Effectiveness of cash-plus programmes on early childhood outcomes compared to cash transfers alone: a systematic review and meta-analysis in low- and middle-income countries. | 10.1371/journal.pmed.1003698 | Little MT, Roelen K, Lange BCL, Steinert JI, Yakubovich AR, Cluver L and Humphreys DK (2021). Effectiveness of cash-plus programmes on early childhood outcomes compared to cash transfers alone: a systematic review and meta-analysis in low- and middle-income countries. *Plos Medicine*, *18*(9), e1003698. https://doi.org/10.1371/journal.pmed.1003698 | No DM1, Wrong publication type |
| Phosphorylated neurofilament heavy chain in cerebrospinal fluid and plasma as a nusinersen treatment response marker in childhood-onset SMA individuals from serbia. | 10.3389/fneur.2024.1394001 | Brkušanin M, Kosać A, Branković-Srećković V, Jovanović K, Perić S, Karanović J, Matijašević Joković S, Garai N, Pešović J, Nikolić D, Stević Z, Brajušković G, Milić-Rašić V and Savić-Pavićević D (2024). Phosphorylated neurofilament heavy chain in cerebrospinal fluid and plasma as a nusinersen treatment response marker in childhood-onset SMA individuals from serbia. *Frontiers in Neurology*, *15*, 1394001. https://doi.org/10.3389/fneur.2024.1394001 | No DM1 |
| Decreased and improved movement abilities in a case of myotonic dystrophy type 1: examining longitudinal characteristics based on repeated evaluations. | 10.7759/cureus.60818 | Ishibashi K, Ishii D, Yamamoto S, Ono Y, Yoshikawa K, Matsuda T, Asakawa Y and Kohno Y (2024). Decreased and improved movement abilities in a case of myotonic dystrophy type 1: Examining longitudinal characteristics based on repeated evaluations. *Cureus*, *16*(5), e60818. https://doi.org/10.7759/cureus.60818 | No cognitive |
| Diabetes mellitus and progression of vascular brain lesions and brain atrophy in patients with symptomatic atherosclerotic disease. the smart-mr study. | 10.1016/j.jns.2013.06.019 | Kooistra M, Geerlings MI, Mali WP, Vincken KL, van der Graaf Y and Biessels GJ (2013). Diabetes mellitus and progression of vascular brain lesions and brain atrophy in patients with symptomatic atherosclerotic disease. the smart-mr study. *Journal of the Neurological Sciences*, *332*(1), 69-74. https://doi.org/10.1016/j.jns.2013.06.019 | No DM1 |
| Evaluation of information-processing speed and neuropsychological functioning in patients with myotonic dystrophy. | 10.1080/01688638708405353 | Stuss DT, Kates MH, Poirier CA, Hylton D, Humphreys P, Keene D and Laflèche G (1987). Evaluation of information-processing speed and neuropsychological functioning in patients with myotonic dystrophy. *Journal of Clinical and Experimental Neuropsychology*, *9*(2), 131-46. https://doi.org/10.1080/01688638708405353 | Wrong study design |
| Effect of mexiletine on muscle stiffness in patients with nondystrophic myotonia evaluated using aggregated n-of-1 trials. | 10.1001/jama.2018.18020 | Stunnenberg BC, Raaphorst J, Groenewoud HM, Statland JM, Griggs RC, Woertman W, Stegeman DF, Timmermans J, Trivedi J, Matthews E, Saris CGJ, Schouwenberg BJ, Drost G, van Engelen BGM and van der Wilt GJ (2018). Effect of mexiletine on muscle stiffness in patients with nondystrophic myotonia evaluated using aggregated n-of-1 trials. *JAMA*, *320*(22), 2344-2353. https://doi.org/10.1001/jama.2018.18020 | No DM1 |
| The development of a model of fatigue in neuromuscular disorders: a longitudinal study. | 10.1016/j.jpsychores.2006.11.014 | Kalkman JS, Schillings ML, Zwarts MJ, van Engelen BG and Bleijenberg G (2007). The development of a model of fatigue in neuromuscular disorders: a longitudinal study. *Journal of Psychosomatic Research*, *62*(5), 571-9. https://doi.org/10.1016/j.jpsychores.2006.11.014 | Wrong publication type |
| Treatment of cataplexy in niemann-pick disease type c with the use of miglustat. | 10.1016/j.ejpn.2010.02.001 | Zarowski M, Steinborn B, Gurda B, Dvorakova L, Vlaskova H and Kothare SV (2011). Treatment of cataplexy in niemann-pick disease type c with the use of miglustat. *European Journal of Paediatric Neurology*, *15*(1), 84-7. https://doi.org/10.1016/j.ejpn.2010.02.001 | No DM1 |
| Congenital muscular dystrophy in jordanian children. | 10.1177/088307389801300804 | Al-Qudah AA and Tarawneh M (1998). Congenital muscular dystrophy in jordanian children.. *Journal of Child Neurology*, *13*(8), 383-6. https://doi.org/10.1177/088307389801300804 | No cognitive |
| Rehospitalization risk of former voluntary and involuntary patients with schizophrenia. | 10.1007/s00127-014-0892-2 | Pfiffner C, Steinert T, Kilian R, Becker T, Frasch K, Eschweiler G, Längle G, Croissant D, Schepp W, Weiser P and Jaeger S (2014). Rehospitalization risk of former voluntary and involuntary patients with schizophrenia.. *Social Psychiatry and Psychiatric Epidemiology*, *49*(11), 1719-27. https://doi.org/10.1007/s00127-014-0892-2 | No DM1 |
| Myotonic dystrophy and progressive cognitive decline: a common condition or two separate problems? | 10.1016/s0010-9452(08)70789-5 | Wilson BA, Balleny H, Patterson K and Hodges JR (1999). Myotonic dystrophy and progressive cognitive decline: a common condition or two separate problems?. *Cortex*, *35*(1), 113-21. https://doi.org/10.1016/s0010-9452(08)70789-5 | Wrong publication type |
| A 5-year follow-up study of an atypical case of myotonic dystrophy. | 10.1080/02699050500283509 | Macniven JA, Graham NL, Davies RR and Wilson BA (2005). A 5-year follow-up study of an atypical case of myotonic dystrophy. *Brain Injury*, *19*(14), 1213-21. https://doi.org/10.1080/02699050500283509 | Wrong publication type |
| Psychotropic drug treatment, clinical characteristics and cognitive processing speed in patients with schizophrenia: Results from the elan study. | 10.1055/s-0031-1297260 | Frasch K, Weiser P, Becker T, Längle G, Steinert T, Niederreiner C, Pfiffner C, Jäger S, Bayer W, Eschweiler GW and Kilian R (2012). Psychotropic drug treatment, clinical characteristics and cognitive processing speed in patients with schizophrenia: results from the elan study. *Pharmacopsychiatry*, *45*(4), 138-45. https://doi.org/10.1055/s-0031-1297260 | No DM1 |
| Vigabatrin with hormonal treatment versus hormonal treatment alone (iciss) for infantile spasms: 18-month outcomes of an open-label, randomised controlled trial. | 10.1016/S2352-4642(18)30244-X | O'Callaghan FJK, Edwards SW, Alber FD, Cortina Borja M, Hancock E, Johnson AL, Kennedy CR, Likeman M, Lux AL, Mackay MT, Mallick AA, Newton RW, Nolan M, Pressler R, Rating D, Schmitt B, Verity CM and Osborne JP (2018). Vigabatrin with hormonal treatment versus hormonal treatment alone (ICISS) for infantile spasms: 18-month outcomes of an open-label, randomised controlled trial. *The Lancet. Child & Adolescent Health*, *2*(10), 715-725. https://doi.org/10.1016/S2352-4642(18)30244-X | No DM1 |
| Proximal myotonic myopathy: Report on Italian families and literature review. | Not found | Meola G, Sansone V, Vitelli E, Mancuso M and Siciliano G (2001). Proximal myotonic myopathy: Report on Italian families and literature review.. *Journal of Clinical Neuromuscular Disease*, *2*(4), 201-10. | No DM1, No cognitive, Wrong publication type |
| Four family members with proximal myotonic myopathy. | Not found | Tieleman AA, van der Velden MP, Visser MC, Wokke JH, Scheffer H and van Engelen BG (2004). Four family members with proximal myotonic myopathy. *Nederlands Tijdschrift Voor Geneeskunde*, *148*(19), 948-52. | No DM1 |

**Table S7.** *Descriptive data and neuropsychological battery used for assessment of the included articles*

| Authors (year) | N | Gender | Phenotype | Inheritance pattern | Time between baseline and follow-up | Age at baseline and follow-up | Neuropsychological battery |
| --- | --- | --- | --- | --- | --- | --- | --- |
| Deutsch et al. (2022) | 80 | 59.3% F | from juvenile onwards | not reported | T1: 3 months ± 2 weeks  T2: 12 months ± 4 weeks | Baseline: x̄ = 43  Follow-up: not reported | Cogstate Digital Cognitive Assessment: DET, IDN, ONB, GMLT |
| Díaz-Leiva et al. (2020) | 31 | 45% F | adult | not reported | 4 years | Baseline: not reported  Follow-up: from 21 to 53 years old | TBR: Digit and Localization spans, and Cubes, WMS-III: Localization span, WAIS-IV: Letter-Number Sequencing, BVRT-C, CPT-II, BTA, TMT-A, TMT-B, TAVEC, and DEX-Sp |
| Echenne et al. (2008) | 32 | 9 F | congenital and childhood | congenital with RD: 9 maternal  congenital without RD: 7 maternal  childhood: 12 maternal | congenital with: Mdn = 16.6 years  congenital without: Mdn = 20.1 years  childhood: Mdn = 15.6 | Patients were born between 1976 and 1999 | WIPPSI, WISC-III, WISC-R or WAIS |
| Gallais et al. (2017) | 115 | 72 F | adult and late | not reported | 9 years | Baseline: x̄ = 43.6 years  Follow-up: x̄ = 52.3 years | CVLT, RCFT, VF: phonemic and semantic, BNT, SCWT, Ruff 2 & 7 Test, WAIS-R |
| Garmendia et al., (2022) | 118 | 69 F | premanifest, juvenile and adult | manifest:12% maternal  premanifest: 35.3% maternal | x̄ = 11.17 years | Baseline: x̄ =48.14 years (premanifest); x̄ = 43.53 years (manifest)  Follow-up: x̄ = 57.15 years (premanifest); x̄ = 55.48 years (manifest) | WAIS-III: Vocabulary, Block design, and Digit span, ROCF, RAVLT, SCWT, RPM, VF: phonemic and semantic, CALCAP |
| Gliem et al., (2019) | 49 | 25 F | adult | not reported | x̄ = 5.5 years | Baseline: x̄ = 42.5 years  Follow-up: x̄ = 48.2 years | NeuroCogFX, BNT, c.I.T., TMT, HAWIE-R, LS, VF: phonemic and semantic |
| Labayru et al., (2020) | 129 | DM1: 54.67% F  HC: 61.12% f | childhood, juvenile, adult and late | not reported | x̄ = 11.62 years | Baseline: x̄ = 38.65 years  Follow-up: x̄ = 50.4 years | WAIS-III: Block design, Digit span, and Vocabulary, Stroop test, CALCAP, RVLT, VF: phonemic and semantic, ROCF, RPM |
| Labayru et al., (2022) | 18 | 9 F | adult and late | 37.5% maternal  50% paternal  12.5% both | x̄ = 9.06 years | Baseline: x̄ = 46.5 years  Follow-up: x̄ = 55.75 years | WAIS-III, ROCF, RAVLT, SCWT, RPM, VF: phonemic and semantic, CALCAP |
| Lindeblad et al. (2019) | 51 | 22 F | Congenital and childhood | Severe congenital: 100% maternal  Mild congenital: 71% maternal, 18% paternal, 11% unknown  Childhood: 72% maternal | x̄ = 7 years 8 months | Baseline:  Severe congenital: x̄ = 9 years 11 months  Mild congenital: x̄ = 11 years 1 months  Childhood: x̄ = 13 years 8 month  Follow-up:  Severe congenital: x̄ = 18 years  Mild congenital: x̄ = 18 years 9 months  Childhood: x̄ = 21 years 5 months | WPPSI-R, WISC-R, WAIS-R |
| Martinello et al., (1999) | 5 | 1 F | congenital | 5 maternal | x̄ = 4 years | Baseline: x̄ = 12 years | RPM, SB, WISC, WAIS |
| Modoni et. al., (2008) | 34 | Juvenile and adult 19 F | congenital, juvenile and adult | not reported | 4 years | Baseline:  E2 group: x̄ = 53.2  E3 group: x̄ = 42.6 | MMSE, RAVLT, RCF: Recall and Copy, DST: forward and backward, VF: phonemic and semantic, Naming of pictures of objects and actions, Stroop, TMT-B, MFTC, RCPM |
| Ricci et al., (2022) | 6 | 6 F | Congenital and childhood | 6 maternal  4 paternal | 2 years | Baseline: x̄ = 9 years 1 month  Follow-up: x̄ = 10 years | BSID-III, WPPSI-III, WISC-IV, TPV: Eye-hand coordination, Position in space, Copying and reproduction and Figure-ground, Bells Test, TOL. |
| Sansone et al., (2007) | 25 | 41.1% F | Adult | 25 maternal | x̄ = 7.3 years | Baseline: x̄ = 44.9 years  Follow-up: not reported | MMSE, RCPM, Token test, VF: phonemic and semantic, DST: forward, Spatial span, Story recall, Rey Recall, RCF Copy, TMT-A, TMT-B, TEA, TOL |
| Tuikka et al., (1993) | 16 | 23 F | congenital and adult | Congenital: maternal  Adult:  10 maternal,  17 paternal | 12 years | Baseline: x̄ = 35.4 years  Follow-up: x̄ = 47.3 years | WAIS, WISC, WMS, BVRT, ROCF, Copy of drawings test, FTT |
| Winbald et al., (2016) | 37 | 19 F | adult | not reported | 5 years | Baseline: 40 years  Follow-up: 45 years | WAIS-R: Vocabulary, Arithmetic, Block design, Picture completion, Spatial span and Digit span, VF: phonemic, RCFT, RAVLT, TMT-A, TMT-B, SCWT-B, WCST |

N = patients included at follow-up that overwent the neuropsychological assessment; data for gender, inheritance pattern, and age at baseline might not be about the follow-up sample but about the baseline sample, depending on how descriptive data were reported in each publication. When data was given for HC and DM1 patients in age at baseline and follow-up, only information regarding DM1 patients is included. In Modoni et al. (2008), two groups are defined based on the number of CTG repeats: E (150-500 CTG repeats) and E3 (500-1000 CTG repeats). Acronyms: F: female; RD: respiratory distress; TBR: Test de Barcelona Revised; WMS: Wechsler Memory Scale; BVRT: Benton Visual Retention Test; TMT: Trail Making Test; TAVEC: Test aprendizaje verbal España-Complutense; WAT-30: Word Accentuation Test-30; DEX-Sp: Spanish version of Disexecutive Questionnaire; DET: Detection; IDN: identification; ONB: One-Back; GMLT: Groton Maze Learning Test; WAIS: Weschler Adult Intelligente Scale; WAIS-R: Weschler Adult Intelligente Scale-Revised; BVRT-C: Benton Visual Retention Test-C; CPT-II: Conner’s Performance Test-II; BTA: Brief Test of Attention; WPPSI: Wechsler Preschool and Primary Scale of Intelligence; WPPSI-R: Wechsler Preschool and Primary Scale of Intelligence-Revised; WISC: Wechsler Intelligence Scale for Children; WISC-R: Wechsler Intelligence Scale for Children-Revised; MMSE: Mini Mental State Examination; WCST: Wisconsin Card Sorting Test; DST: Digit Span Test; SDMT: Symbol Digit Modalities Test; CVLT: California Verbal Learning Test; RCFT: Rey Complex Figure Test; ROCF: Rey-Osterrieth Complex Figure Test; VF: Verbal Fluency; BNT: Boston Naming Test; SCWT: Stroop Color and Word Test; Ruff 2 & 7 Test: Ruff 2 & 7 Selective Attention Test; RVLT: Rey Verbal Learning Test; RAVLT: Rey Auditory Verbal Learning Test; RPM: Raven’s Progressive Matrices; RCPM: Raven’s Coloured Progressive Matrices; CALCAP: California Computerized Assessment Package; c.I.T.: Cerebral Insuffizienztest; HAWIE-R: Hamburg-Wechsler Intelligenztest für Erwachsene-Revised; LS: Leistung-sprüfsystem; TEA: Alertness and Divided attention; SB: Stanford-Binet Intelligence Scale; MFTC: Multiple Features Targets Cancellation test; BSID: Bayley Scales of Infant and Toddler Development; TPV: Visual perception and visuo-motor integration test; TOL: Tower of London; FTT: Finger-Tapping Test.
